# Supplementary material for: Programming Escherichia coli to function as a digital display
Source: Mol Syst Biol. 2020 Mar 5;16(3):e9401. doi: 10.15252/msb.20199401 (PMC7058928; doi:10.15252/msb.20199401)
Supplement: Supplementary file 1 — Appendix [file MSB-16-e9401-s001.pdf]

Table of contents

Appendix Information I: Mathematical model of tandem promoters
Appendix Information II: Model of gate dynamics
Appendix Information III: Model of circuit dynamics
Appendix Figure S1: Biophysical model of tandem promoters
Appendix Figure S2: Gate modifications to improve performance
Appendix Figure S3: Modification of the OC6 sensor
Appendix Figure S4: Evaluation of tandem promoters: experiments versus the predictions
Appendix Figure S5: Circuit prediction using UCF Eco1C1G1T1 (first UCF)
Appendix Figure S6: Roadblocking assays for the gate output promoters
Appendix Figure S7: Activity of sensor output promoters in tandem
Appendix Figure S8: Characterization of gates
Appendix Figure S9: Characterization of genetic sensors
Appendix Figure S10: Detailed designs and data for the 7-segment circuits
Appendix Figure S11: Response and growth impact of the 7-segment circuits
Appendix Figure S12: Fit of promoter models to response data
Appendix Figure S13: Analysis of Segment F (low copy plasmid) after the 88-hour continuous switching experiment
Appendix Figure S14: Characterization and stability of the first set of 7-segment designs carried on a higher-copy plasmid
Appendix Figure S15: Analysis of Segment F and G (on high copy plasmids) after the 88-hour continuous switching experiment.
Appendix Figure S16: 3D printed device and seven segment display
Appendix Figure S17: Plasmid backbones
Appendix Figure S18: High copy plasmid backbone
Appendix Figure S19: Plasmid maps for the sensor characterization
Appendix Figure S20: Plasmid maps for the sensor output tandem promoter characterization
Appendix Figure S21: Plasmid maps for the gate characterization
Appendix Figure S22: Plasmid maps for the gate output tandem promoter characterization
Appendix Figure S23: Plasmid maps for the tandem promoter evaluation
Appendix Figure S24: Plasmid maps of the 7-segment circuits
Appendix Figure S25: Plasmid maps for the YFP expression from a constitutive promoter in two p15A backbones
Appendix Table S1: Parameterization of sensors
Appendix Table S2: List of plasmids used in each figure
Appendix Table S3: Complete annotated sequences of gates and sensors
Appendix Table S4: Genetic part sequences used in this work
Appendix Table S5: Sequences of the 7-segment circuits
Appendix Reference

Appendix Information I. **Mathematical model of tandem promoters**

Non-additive model

Consider two promoters oriented in series whose RNAP fluxes are y_1 (upstream) and y_2 (downstream), respectively. If there is no interaction between the RNAP fluxes originating from the two promoters, they can additively contribute to the total RNAP flux,

$$y_{tot} = y_1 + y_2 \quad . \tag{S1}$$

In this case, the empirical response functions (Equation 1) for each promoter could be substituted for y_1 and y_2.

To demonstrate how non-additivity impacts Appendix Equation S1, we derive the promoter activities using a Shea-Ackers formalism (Ackers et al, 1982) and a simple biophysical model where a single repressor binds non-cooperatively to each promoter. All of the possible promoter states are enumerated in Appendix Figure S1 when repressor R_1 binds to input promoter 1 with affinity $K_{R,1}$, repressor R_2 binds to input promoter 2 with affinity $K_{R,2}$, and RNAP can bind to either promoter with affinities $K_{RNAP,1}$ and $K_{RNAP,2}$. First, for the additive case, the ensemble of all possible states is given by

$$Z = 1 + K_{RNAP,1}[RNAP] + K_{RNAP,2}[RNAP] + K_{R,1}[R_1] + K_{R,2}[R_2] + K_{R,1}K_{R,2}[R_1][R_2] + K_{R,1}K_{RNAP,2}[R_1][RNAP] + K_{RNAP,1}K_{R,2}[RNAP][R_2] + K_{RNAP,1}K_{RNAP,2}[RNAP]^2 \quad , \tag{S2}$$

which can be simply factored into the sum of the individual contributions from each promoter. The probability P that either promoter is in a state where RNAP is binding and initiating transcription is

$$P = Z^{-1}\left(K_{RNAP,1}[RNAP] + K_{RNAP,2}[RNAP] + K_{RNAP,1}K_{R,2}[RNAP][R_2] + K_{RNAP,2}K_{R,1}[RNAP][R_1] + K_{RNAP,1}K_{RNAP,2}[RNAP]^2\right) . \tag{S3}$$

This can be factored and the total transcription rate from the two promoters calculated as

$$\frac{x_{Tot}}{[DNA]} = \frac{x_1}{[DNA]}\frac{K_{RNAP,1}[RNAP]}{1+K_{RNAP,1}[RNAP]+K_{R,1}[R_1]} + \frac{x_2}{[DNA]}\frac{K_{RNAP,2}[RNAP]}{1+K_{RNAP,2}[RNAP]+K_{R,2}[R_2]} \quad , \tag{S4}$$

where x_1/[DNA] and x_2/[DNA] are the RNA fluxes from the two promoters.

Appendix Equations S3 and S4 are based on the complete binding polynomial that results from the consideration of all binding states to the promoters. The gate response function (Equation 1) represents an empirical form of this equation. This empirical form does not account for all of the binding states of the promoter, but allows for cooperativity (not necessarily due to multimer formation), and the maximum and minimum promoter activities are provided by sets of experiments (measured in RPU). When these equations are substituted into Equation 1, this yields

$$y = \left[y_{min,1} + \left(y_{max,1} - y_{min,1}\right)\frac{{K_1}^{n_1}}{{K_1}^{n_1} + {x_1}^{n_1}}\right] + \left[y_{min,2} + \left(y_{max,2} - y_{min,2}\right)\frac{{K_2}^{n_2}}{{K_2}^{n_2} + {x_2}^{n_2}}\right] \quad , \tag{S5}$$

where the parameters are defined in the main text. When compared to Appendix Equation S4, the RNAP binding, unbound DNA state, and transcription rates are captured by y_{min} and y_{max}. The fractions are related to the binding of each repressor to its cognate promoter.

Non-additivity due to roadblocking only impacts the upstream promoter (first term of Appendix

Equation S4). Within this term, roadblocking only affects the promoter state when RNAP is bound to the upstream promoter and R_2 is bound to the downstream promoter (Appendix Figure S1). This does not have the effect of completely blocking transcription. Instead, there is a probability that R_2 blocks transcription, thus reducing the contribution of transcription from the upstream promoter to the total transcription rate by a term β. To calculate the rate from the first promoter, the corresponding portion of Appendix Equation S3 can be re-written as

$$P_1 = Z^{-1}\left(K_{RNAP,1}[RNAP] + \beta K_{RNAP,1}K_{R,2}[RNAP][R_2] + K_{RNAP,1}K_{RNAP,2}[RNAP]^2\right) \quad . \tag{S6}$$

With this modified form, the first term of Appendix Equation S4 can be simplified to

$$\frac{x_1}{[DNA]}\left(\frac{1+K_{RNAP,2}[RNAP]+\beta K_{R,2}[R_2]}{1+K_{RNAP,2}[RNAP]+K_{R,2}[R_2]}\right)\left(\frac{K_{RNAP,1}[RNAP]}{1+K_{RNAP,1}[RNAP]+K_{R,1}[R_1]}\right) \quad , \tag{S7}$$

where the second term is simply the probability of transcription occurring from promoter 1 and the first term is a correction due to R_2 blocking transcription.

Finally, an additional term α is included to capture all of the non-additive effects due to the existence of a downstream promoter. This includes interference due to pause sites, antisense transcription, binding of other proteins, and other mechanisms (Brophy & Voigt, 2016; Dahirel et al, 2009; Roberts, 2014). The addition of this parameter to Equation S7 yields

$$\frac{x_1}{[DNA]}\alpha\left(\frac{1+K_{RNAP,2}[RNAP]+\beta K_{R,2}[R_2]}{1+K_{RNAP,2}[RNAP]+K_{R,2}[R_2]}\right)\left(\frac{K_{RNAP,1}[RNAP]}{1+K_{RNAP,1}[RNAP]+K_{R,1}[R_1]}\right) \quad . \tag{S8}$$

These effects are assumed to uniformly reduce transcription from the upstream promoter. In other words, it neither depends on the state of the downstream promoter nor the level of transcription from the upstream one.

As above, the equivalent of Appendix Equation S8 can be derived for the empirical response functions. This results in the form

$$y = y_{min,2} + \left(y_{max,2} - y_{min,2}\right)\frac{K_2{}^{n_2}}{K_2{}^{n_2} + x_2{}^{n_2}} + \alpha\left(\frac{K_2{}^{n_2} + \beta\, x_2{}^{n_2}}{K_2{}^{n_2} + x_2{}^{n_2}}\right)\left[y_{min,1} + \left(y_{max,1} - y_{min,1}\right)\frac{K_1{}^{n_1}}{K_1{}^{n_1} + x_1{}^{n_1}}\right] \quad , \tag{S9}$$

where α and β are parameters associated with promoter 2 and capture its non-additive impact on an upstream promoter. Smaller values of both indicate more interference and as α and β approach unity, the interference effects go to zero.

Simplified model for characterizing sensors

A sensor's output promoter can also cause roadblocking. The response function of a sensor captures how the output promoter changes as a function of the stimulus (*e.g.*, the concentration of inducer). If the response function is known, then the equations from the previous section can be applied with some modifications, described in this section. The input of a sensor's response function (x-axis) is the concentration of the inducer *c*, as opposed to a promoter activity in RPU. Also, the response function turns on as a function of *c*, which is the opposite of a gate where the output promoter turns off as a function of the input promoter. Thus, the form of the response follows that of an activator (regardless of whether the inducer activates an activator or derepresses a repressor), written by

$$y = y_{min} + (y_{max} - y_{min})\frac{c^n}{K^n + c^n} \quad . \tag{S10}$$

where *c* is the concentration of inducers. With these modifications, the equation describing roadblocking by an input promoter at position 2 is

$$y = y_{min,2} + (y_{max,2} - y_{min,2})\frac{{c_2}^{n_2}}{{K_2}^{n_2} + {c_2}^{n_2}} + \alpha\left(\frac{\beta\, {K_2}^{n_2} + {c_2}^{n_2}}{{K_2}^{n_2} + {c_2}^{n_2}}\right)\left[y_{min,1} + (y_{max,1} - y_{min,1})\frac{{K_1}^{n_1}}{{K_1}^{n_1} + {x_1}^{n_1}}\right] . \tag{S11}$$

A simpler form can be used because the inputs to Cello are only the RNAP flux of the output promoter for two states, for example the absence or presence of a defined concentration of inducer (OFF or ON). Considering this, the interference term of Appendix Equation S11 can be rearranged as follows

$$\frac{\beta {K_2}^{n_2} + {c_2}^{n_2}}{{K_2}^{n_2} + {c_2}^{n_2}} = \beta + (1-\beta)\left(\frac{{c_2}^{n_2}}{{K_2}^{n_2} + {c_2}^{n_2}}\right) \quad . \tag{S12}$$

Note that the fraction is the same in the input term (position 2) in Appendix Equation S11. When the sensor output is H, this fraction goes to 1 and $y_2 = y_{max,2.}$ When the sensor output is OFF, this fraction term goes to 0 and $y_2 = y_{min,2}$. Here, $y_{min,2}$ and $y_{max,2}$ represent the OFF and ON values of the sensor in Cello. When the sensor is ON, Appendix Equation S12 goes to 1 and when it is OFF, it goes to β. This simplifies the impact of the interference parameters and Appendix Equation S11 becomes

$$y = \alpha\beta^{(1-q)}\left[y_{min,1} + (y_{max,1} - y_{min,1})\frac{{K_1}^{n_1}}{{K_1}^{n_1} + {x_1}^{n_1}}\right] + \delta(1-q)(y_{max,2} - y_{min,2}) + y_{min,2}, \tag{S13}$$

where q is 0 when the sensor is OFF or 1 when the sensor is ON. Here, the delta function $\delta(x)$ is defined as 1 when x is zero, and zero otherwise.

Appendix Information II. Model of gate dynamics

A gate model is developed that captures the speed by which the gate reaches its steady-state value after a change in the input. This model does not require the multitude of hard-to-measure kinetic parameters required by a detailed biophysical model. Rather, we simply consider characteristic times for the circuit to adjust after the perturbation to the input. We consider two parameters: one that captures the response to go to a steady-state that is higher than the current output τ_y^{ON} and one to go to a steady-state that is lower τ_y^{OFF}. The values of these parameters are expected to be different because the underlying biophysics determining the timescale differs. For each gate, the corresponding conditional ordinary differential equation is

$$\frac{dy}{dt} = \begin{cases} \tau_y^{ON}(y_{ss} - y) & if\ y < y_{ss} \\ \tau_y^{OFF}(y_{ss} - y) & otherwise \end{cases} , \tag{S14}$$

where y_{ss} is the steady state of the gate given its input sequences, as calculated using the response function (Equation 1).

Experiments were then designed to extract the two characteristic times for each gate. To measure the response of each NOT gate, a sensor is connected to serve as the input and the output is measured by connecting the output promoter to the transcription of *yfp*. Cells carrying this circuit are grown in the presence of inducer until reaching steady-state, then switched into media lacking inducer and fluorescence is measured as the gate turns on. The same is done in reverse, where cells are grown in the absence of inducer, then switched into media containing it, and the loss of fluorescence is measured over time until steady-state is reached. However, using only these data is problematic as the sensor (addition/removal of inducer to the turning on of the sensor output promoter) and the expression/degradation of YFP both have induction and relaxation timescales that need to be separated from those of the gate.

We use data from the sensor to estimate these parameters. First, the sensor is grown under conditions with inducer and then moved to conditions lacking inducer and the loss of YFP fluorescence is measured over time (Appendix Figure S9). The time for the sensor to turn off (the binding of the repressor without inducer to the promoter) is fast with respect to YFP degradation, whose half-life is expected to be about the cell doubling time. This simple exponential decay can be modeled by

$$\frac{d[YFP]}{dt} = -\tau_{YFP}^{OFF}\ ([YFP] - [YFP]_{min}), \tag{S15}$$

where [YFP] is the fluorescence as measured by cytometry and min refers to the steady-state value in the absence of inducer. This is fit to the data and τ_{YFP}^{OFF} = 1.06 hr^{-1}. The timescale of the off rate of the sensor is selected to be as slow as possible without impacting the above fit. This leads to τ_x^{OFF} = 4.0 hr^{-1}, which is held constant for all of the sensors.

The next step is to obtain the YFP production rate. This is calculated by considering the equation for the production and degradation of YFP

$$\frac{d[YFP]}{dt} = \tau_{YFP}^{ON}\, y - \tau_{YFP}^{OFF}\ [YFP] , \tag{S16}$$

where *y* is the RNAP flux from gate's and sensor's output promoter reported in RPU (the units of [YFP] are the fluorescence in au). At steady-state, Appendix Equation S16 becomes

$$\tau_{YFP}^{ON} = \tau_{YFP}^{OFF} \frac{[YFP]_{max}}{y_{max}} \quad , \tag{S17}$$

where $[YFP]_{max}$ is the highest fluorescence measured when uninduced (for gates) or fully induced (for sensors), and y_{max} is the maximum RPU from the gate and sensor output promoter. This leads to τ_{YFP}^{ON}, which differs for each gate and sensor (1163 au-RPU^{-1}hr^{-1}). The following equations were used to extract τ_x^{ON} from data from turning the sensor on (OFF to ON),

$$\frac{dx}{dt} = \tau_x^{ON}(x_{SS} - x) \quad \text{and} \tag{S18}$$

$$\frac{d[YFP]}{dt} = \tau_{YFP}^{ON}\, x - \tau_{YFP}^{OFF}\ [YFP] \quad , \tag{S19}$$

where x is the RNAP flux from sensor's output promoter reported in RPU, and τ_x^{OFF}, τ_{YFP}^{ON}, and τ_{YFP}^{OFF} were determined above.

Using these parameters, equations can be written to extract on- and off- rates for a gate. For the case when cells are grown in the presence of inducer and then switched to media lacking inducer (the gate output goes from OFF to ON),

$$\frac{dx}{dt} = \tau_x^{OFF}\ (x_{SS} - x) \quad , \tag{S20}$$

$$\frac{dy}{dt} = \tau_y^{ON}(y_{ss} - y) \quad , \quad \text{and} \tag{S21}$$

$$\frac{d[YFP]}{dt} = \tau_{YFP}^{ON}\, y - \tau_{YFP}^{OFF}\ [YFP] \quad , \tag{S22}$$

where the only unknown parameter is τ_y^{ON}. These equations are solved and fit to the data (Appendix Figure S8) to obtain the characteristic on time for each gate (Table 1).

Finally, the off-times of the gates can be determined by growing the cells in the absence of inducer and then switching them into media containing the inducer (Methods). Modeling this change results in the following equations

$$\frac{dx}{dt} = \tau_x^{ON}(x_{SS} - x) \quad , \tag{S23}$$

$$\frac{dy}{dt} = \tau_y^{OFF}(y_{ss} - y) \quad , \quad \text{and} \tag{S24}$$

$$\frac{d[YFP]}{dt} = \tau_{YFP}^{ON}\, y - \tau_{YFP}^{OFF}\ [YFP] \quad , \tag{S25}$$

where τ_y^{OFF} is the only unknown parameter. These equations are solved and fit to the data for each gate (Appendix Figure S8) and the resulting parameters are shown in Table 1.

Appendix Information III. Model of circuit dynamics

The prediction of the dynamic behavior of circuits requires the time-dependent response that is accumulated from all gates. The following is the equation set for Segment G.

Segment G

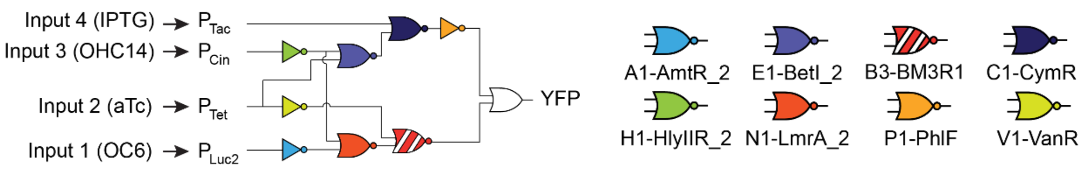

For the sensors:

$$\frac{dx_{Lux2}}{dt} = \begin{cases} \tau_{Lux2}^{ON}\left(x_{Lux2,SS} - x_{Lux2}\right) & \text{if } x_{Lux2} < x_{Lux2,SS} \\ \tau_{Lux2}^{OFF}\left(x_{Lux2,SS} - x_{Lux2}\right) & \text{otherwise} \end{cases}, \qquad \text{(S26)}$$

$$\frac{dx_{Tet}}{dt} = \begin{cases} \tau_{Tet}^{ON}\left(x_{Tet,SS} - x_{Tet}\right) & \text{if } x_{Tet} < x_{Tet,SS} \\ \tau_{Tet}^{OFF}\left(x_{Tet,SS} - x_{Tet}\right) & \text{otherwise} \end{cases}, \qquad \text{(S27)}$$

$$\frac{dx_{Cin}}{dt} = \begin{cases} \tau_{Cin}^{ON}\left(x_{Cin,SS} - x_{Cin}\right) & \text{if } x_{Cin} < x_{Cin,SS} \\ \tau_{Cin}^{OFF}\left(x_{Cin,SS} - x_{Cin}\right) & \text{otherwise} \end{cases}, \qquad \text{(S28)}$$

$$\frac{dx_{Tac}}{dt} = \begin{cases} \tau_{Tac}^{ON}\left(x_{Tac,SS} - x_{Tac}\right) & \text{if } x_{Tac} < x_{Tac,SS} \\ \tau_{Tac}^{OFF}\left(x_{Tac,SS} - x_{Tac}\right) & \text{otherwise} \end{cases}, \qquad \text{(S29)}$$

For the gates:

$$\frac{dy_{VanR}}{dt} = \begin{cases} \tau_{VanR}^{ON}\left(y_{VanR,min} + \left(y_{VanR,max} - y_{VanR,min}\right)\frac{K_{VanR}{}^{n_{VanR}}}{K_{VanR}{}^{n_{VanR}} + x_{Tet}{}^{n_{VanR}}} - y_{VanR}\right) & \text{if } y_{VanR} < y_{VanR,SS} \\ \tau_{VanR}^{OFF}\left(y_{VanR,min} + \left(y_{VanR,max} - y_{VanR,min}\right)\frac{K_{VanR}{}^{n_{VanR}}}{K_{VanR}{}^{n_{VanR}} + x_{Tet}{}^{n_{VanR}}} - y_{VanR}\right) & \text{otherwise} \end{cases}, \qquad \text{(S30)}$$

$$\frac{dy_{PhlF}}{dt} = \begin{cases} \tau_{PhlF}^{ON}\left(y_{PhlF,min} + \left(y_{PhlF,max} - y_{PhlF,min}\right)\frac{K_{PhlF}{}^{n_{PhlF}}}{K_{PhlF}{}^{n_{PhlF}} + y_{CymR}{}^{n_{PhlF}}} - y_{PhlF}\right) & \text{if } y_{PhlF} < y_{PhlF,SS} \\ \tau_{PhlF}^{OFF}\left(y_{PhlF,min} + \left(y_{PhlF,max} - y_{PhlF,min}\right)\frac{K_{PhlF}{}^{n_{PhlF}}}{K_{PhlF}{}^{n_{PhlF}} + y_{CymR}{}^{n_{PhlF}}} - y_{PhlF}\right) & \text{otherwise} \end{cases}, \qquad \text{(S31)}$$

$$\frac{dy_{AmtR2}}{dt} = \begin{cases} \tau_{AmtR2}^{ON}\left(y_{AmtR2,min} + \left(y_{AmtR2,max} - y_{AmtR2,min}\right)\frac{K_{AmtR2}{}^{n_{AmtR2}}}{K_{AmtR2}{}^{n_{AmtR2}} + x_{Lux2}{}^{n_{AmtR2}}} - y_{AmtR2}\right) & \text{if } y_{AmtR2} < y_{AmtR2,SS} \\ \tau_{AmtR2}^{OFF}\left(y_{AmtR2,min} + \left(y_{AmtR2,max} - y_{AmtR2,min}\right)\frac{K_{AmtR2}{}^{n_{AmtR2}}}{K_{AmtR2}{}^{n_{AmtR2}} + x_{Lux2}{}^{n_{AmtR2}}} - y_{AmtR2}\right) & \text{otherwise} \end{cases}, \qquad \text{(S32)}$$

$$\frac{dy_{BM3R1}}{dt} = \begin{cases} \tau_{BM3R1}^{ON}\left(y_{BM3R1,min} + \left(y_{BM3R1,max} - y_{BM3R1,min}\right)\frac{K_{BM3R1}{}^{n_{BM3R1}}}{K_{BM3R1}{}^{n_{BM3R1}} + f\left(y_{VanR}, y_{LmrA2}\right)^{n_{BM3R1}}} - y_{BM3R1}\right) & \text{if } y_{BM3R1} < y_{BM3R1,SS} \\ \tau_{BM3R1}^{OFF}\left(y_{BM3R1,min} + \left(y_{BM3R1,max} - y_{BM3R1,min}\right)\frac{K_{BM3R1}{}^{n_{BM3R1}}}{K_{BM3R1}{}^{n_{BM3R1}} + f\left(y_{VanR}, y_{LmrA2}\right)^{n_{BM3R1}}} - y_{BM3R1}\right) & \text{otherwise} \end{cases}, \qquad \text{(S33)}$$

$$\frac{dy_{LmrA2}}{dt} = \begin{cases} \tau_{LmrA2}^{ON}\left(y_{LmrA2,min} + \left(y_{LmrA2,max} - y_{LmrA2,min}\right)\frac{K_{LmrA2}{}^{n_{LmrA2}}}{K_{LmrA2}{}^{n_{LmrA2}} + f\left(y_{HlyIIR2}, y_{AmtR2}\right)^{n_{LmrA2}}} - y_{LmrA2}\right) & \text{if } y_{LmrA2} < y_{LmrA2,SS} \\ \tau_{LmrA2}^{OFF}\left(y_{LmrA2,min} + \left(y_{LmrA2,max} - y_{LmrA2,min}\right)\frac{K_{LmrA2}{}^{n_{LmrA2}}}{K_{LmrA2}{}^{n_{LmrA2}} + f\left(y_{HlyIIR2}, y_{AmtR2}\right)^{n_{LmrA2}}} - y_{LmrA2}\right) & \text{otherwise} \end{cases}, \qquad \text{(S34)}$$

$$\frac{dy_{HlyIIR2}}{dt} = \begin{cases} \tau_{HlyIIR2}^{ON}\left(y_{HlyIIR2,min} + \left(y_{HlyIIR2,max} - y_{HlyIIR2,min}\right)\frac{K_{HlyIIR2}{}^{n_{HlyIIR2}}}{K_{HlyIIR2}{}^{n_{HlyIIR2}} + x_{Cin}{}^{n_{HlyIIR2}}} - y_{HlyIIR2}\right) & if\ y_{HlyIIR2} < y_{HlyIIR2,SS} \\ \tau_{HlyIIR2}^{OFF}\left(y_{HlyIIR2,min} + \left(y_{HlyIIR2,max} - y_{HlyIIR2,min}\right)\frac{K_{HlyIIR2}{}^{n_{HlyIIR2}}}{K_{HlyIIR2}{}^{n_{HlyIIR2}} + x_{Cin}{}^{n_{HlyIIR2}}} - y_{HlyIIR2}\right) & otherwise \end{cases} , \quad (S35)$$

$$\frac{dy_{BetI2}}{dt} = \begin{cases} \tau_{BetI2}^{ON}\left(y_{BetI2,min} + \left(y_{BetI2,max} - y_{BetI2,min}\right)\frac{K_{BetI2}{}^{n_{BetI2}}}{K_{BetI2}{}^{n_{BetI2}} + f(y_{HlyIIR2}, x_{Tet})^{n_{BetI2}}} - y_{BetI2}\right) & if\ y_{BetI2} < y_{BetI2,SS} \\ \tau_{BetI2}^{OFF}\left(y_{BetI2,min} + \left(y_{BetI2,max} - y_{BetI2,min}\right)\frac{K_{BetI2}{}^{n_{BetI2}}}{K_{BetI2}{}^{n_{BetI2}} + f(y_{HlyIIR2}, x_{Tet})^{n_{BetI2}}} - y_{BetI2}\right) & otherwise \end{cases} , \quad (S36)$$

$$\frac{dy_{CymR}}{dt} = \begin{cases} \tau_{CymR}^{ON}\left(y_{CymR,min} + \left(y_{CymR,max} - y_{CymR,min}\right)\frac{K_{CymR}{}^{n_{CymR}}}{K_{CymR}{}^{n_{CymR}} + f(x_{Tac}, y_{BetI2})^{n_{CymR}}} - y_{CymR}\right) & if\ y_{CymR} < y_{CymR,SS} \\ \tau_{CymR}^{OFF}\left(y_{CymR,min} + \left(y_{CymR,max} - y_{CymR,min}\right)\frac{K_{CymR}{}^{n_{CymR}}}{K_{CymR}{}^{n_{CymR}} + f(x_{Tac}, y_{BetI2})^{n_{CymR}}} - y_{CymR}\right) & otherwise \end{cases} , \quad (S37)$$

$$\frac{dYFP}{dt} = \tau_{YFP}^{ON}\, f(y_{PhlF}, y_{BM3R1}) - \tau_{YFP}^{OFF}\, YFP \quad . \quad (S38)$$

In the above equations, roadblocking is accounted for by the function

$$f(u,d) = y_u\, \alpha\left(\frac{y_d - y_{d,min} + \beta(y_{d,max} - y_d)}{y_{d,max} - y_{d,min}}\right) + y_d \quad , \quad (S39)$$

where *u* and *d* in *f*(*u*,*d*) capture the upstream and downstream promoter in a tandem promoter (Appendix Equation S9 and S13).

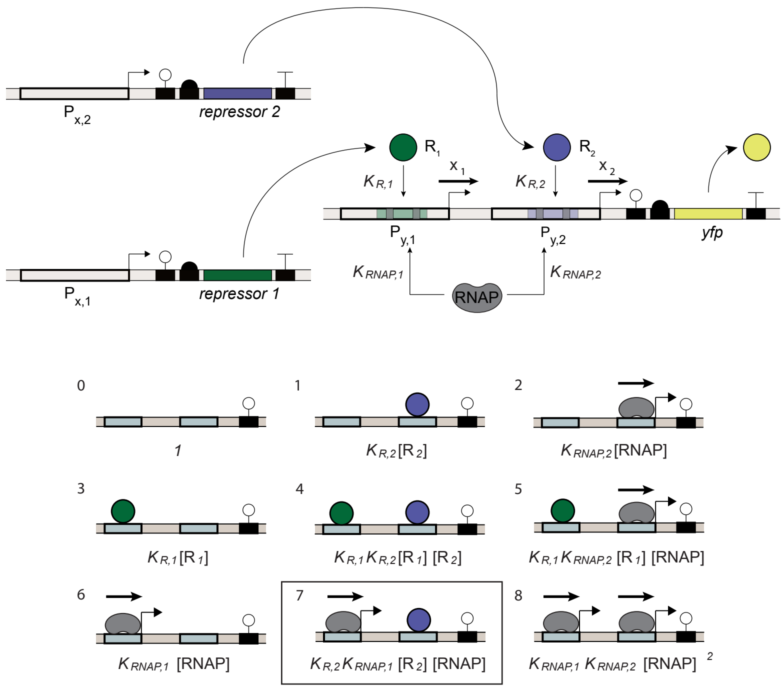

__Appendix Figure S1__: **Biophysical model of tandem promoter.** This diagram corresponds to the derivation of the non-additive promoter model in Appendix Information I (the parameters are defined in that section). The schematic at the top shows two tandem promoters that are the output promoters from two upstream gates. Roadblocking can occur when the repressor bound to the downstream promoter (R2) interferes with transcription of the upstream promoter. The binding of the repressors and RNAP to the promoters are assumed to be mutually exclusive, as indicated with overlapping operators. The enumeration of binding states to the tandem promoters is shown, following a Shea-Ackers formalism (Ackers et al, 1982). The arrows above the promoters demarcate the states where transcriptoin is active. The boxed state is when roadblocking potentially occurs.

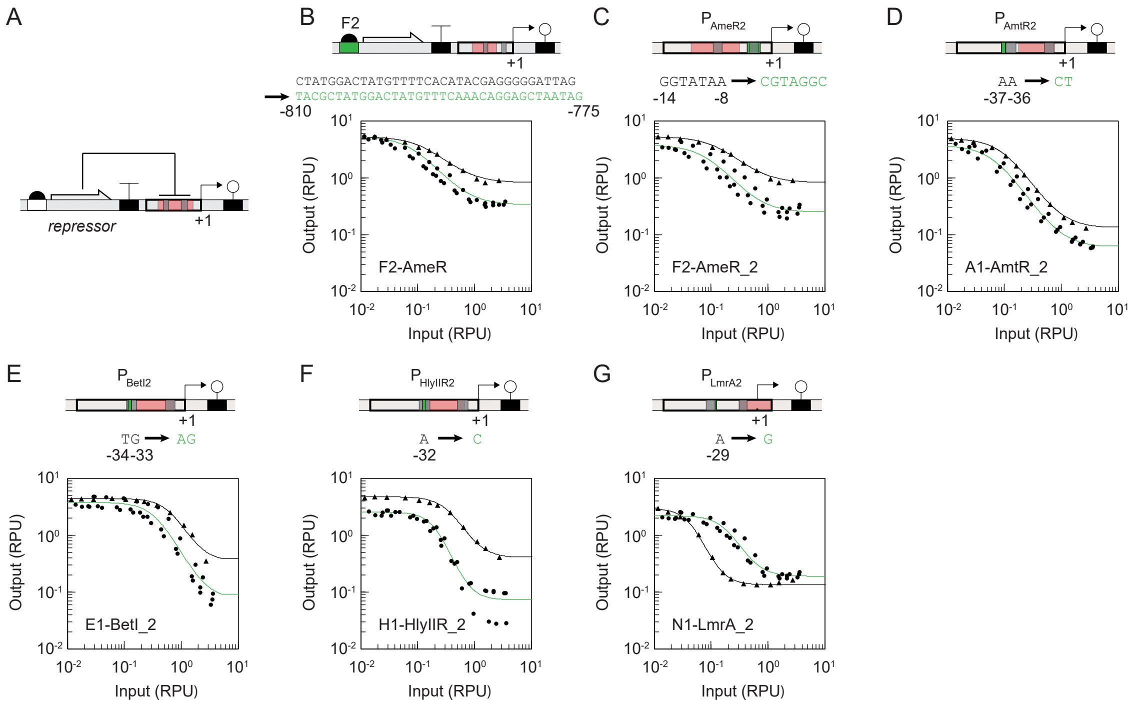

Appendix Figure S2: **Gate modifications to improve performance. (A)** Schematic diagram of a NOT gate. **(B - G)** Comparison of parent gates and modified gates. The black solid line shows the response function of parent gate and the green lines are the response functions of the improved gates. The mutations to either the RBS or promoter are shown, corresponding to the line color. Dark grey indicates -10 and -35 boxes and red represents the operator. The gate in (B) involved a replacement of the RBS driving repressor expression and (C to G) are modifications to the output promoter. The data correspond to three experiments performed on different days. The lines are the best fit to Equation 1 (Table 1).

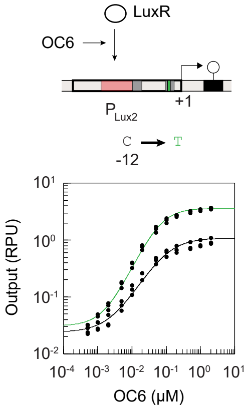

<u>Appendix Figure S3</u>: **Modification of the OC6 sensor.** A single mutation was made at the -10 region of the P_{Lux}* promoter (black) to make P_{Lux2} (green). The solid lines show the response functions before (black) and after (green) the mutation. Dark grey indicates -10 and -35 boxes and red represents the operator. The fit lines are the best fit to Appendix Equation S10 (Appendix Table S1). The data points correspond to three experiments performed on different days.

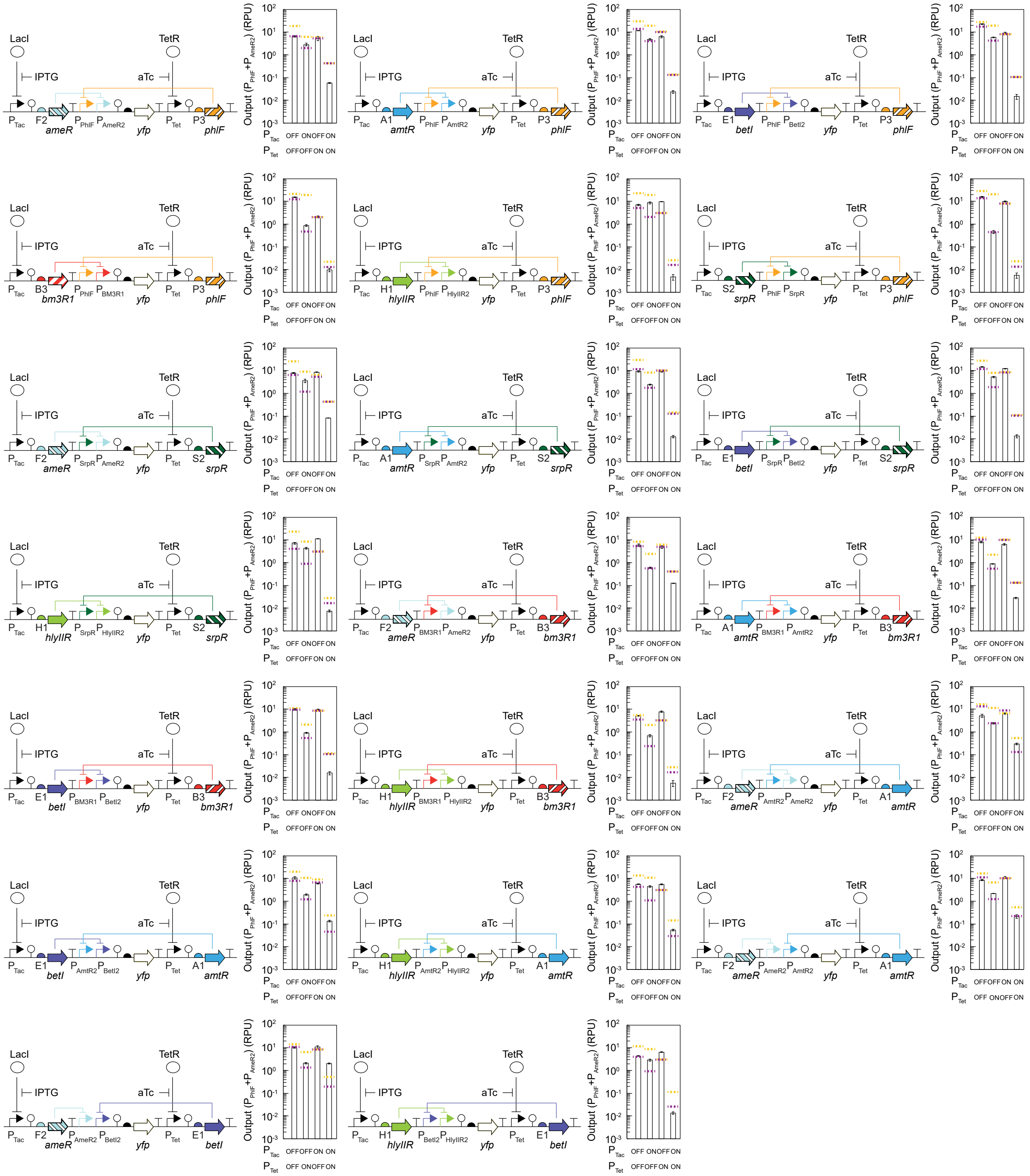

<u>Appendix Figure S4</u>: **Evaluation of tandem promoters, experiments versus the predictions.** These data correspond to the dot plots shown in Figure 1D, E. Each schematic shows the circuits diagram for a NAND gate, designed to have the output of two NOT gates serve as the input to an OR gate (two promoters in series). The bar graphs show the steady-state response for combinations of inducers (5 ng/mL aTc, 1 mM IPTG); error bars are the standard deviation from three experiments performed on different days. The colored lines on the bar graphs are the two models for promoters in series: additive (yellow) and non-additive (purple). Note that the high-copy mutant of the p15a plasmids were used for this analysis, including the parameterization of the model for combining the NOT gate response functions to determine the predicted output (plasmid maps in Appendix Fig S23).

A

```
=========== Assigned circuits ================
assigned lcs: 0
Total elapsed time for assignment algorithm: 115818 milliseconds


////////////////////////////////////////////////////////////
////////   No assignments found. Exiting Cello.   /////////
////////////////////////////////////////////////////////////
```

B

```
=========== Assigned circuits ================
assigned lcs: 0
Total elapsed time for assignment algorithm: 115818 milliseconds


////////////////////////////////////////////////////////////
////////   No assignments found. Exiting Cello.   /////////
////////////////////////////////////////////////////////////
```

C

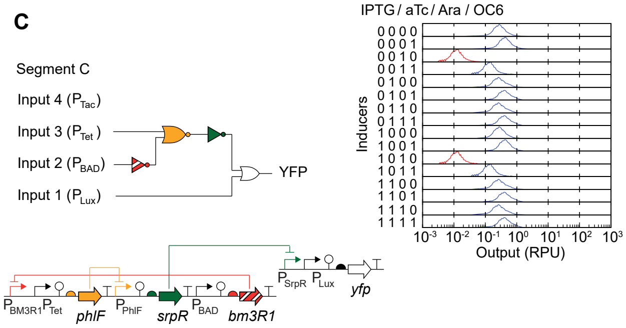

D

```
=========== Assigned circuits ================
assigned lcs: 0
Total elapsed time for assignment algorithm: 115818 milliseconds


////////////////////////////////////////////////////////////
////////   No assignments found. Exiting Cello.   /////////
////////////////////////////////////////////////////////////
```

E

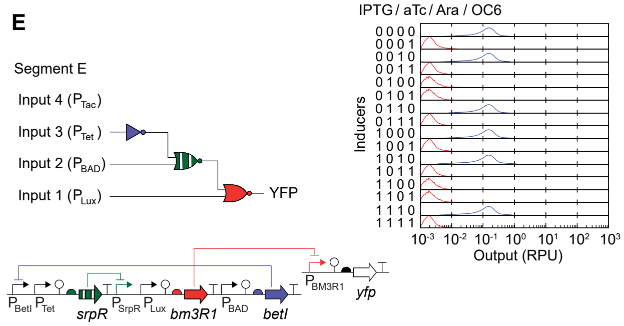

F

```
=========== Assigned circuits ================
assigned lcs: 0
Total elapsed time for assignment algorithm: 115818 milliseconds


////////////////////////////////////////////////////////////
////////   No assignments found. Exiting Cello.   /////////
////////////////////////////////////////////////////////////
```

G

```
=========== Assigned circuits ================
assigned lcs: 0
Total elapsed time for assignment algorithm: 115818 milliseconds


////////////////////////////////////////////////////////////
////////   No assignments found. Exiting Cello.   /////////
////////////////////////////////////////////////////////////
```

Appendix Figure S5: **Circuit prediction using UCF Eco1C1G1T1 (first UCF).** Cello predictions are shown using the previously published version of the UCF (cellocad.org using UCF, Eco1C1G1T1), which excludes promoters that roadblock (according to a threshold) from the downstream position (Nielsen et al, 2016). Only the circuits for Segment C and E, notably the smallest circuits (containing three gates), could be designed. No solutions for the other circuits were found (the error message is shown). Blue and red contour lines indicate the predicted ON and OFF, respectively (C and E).

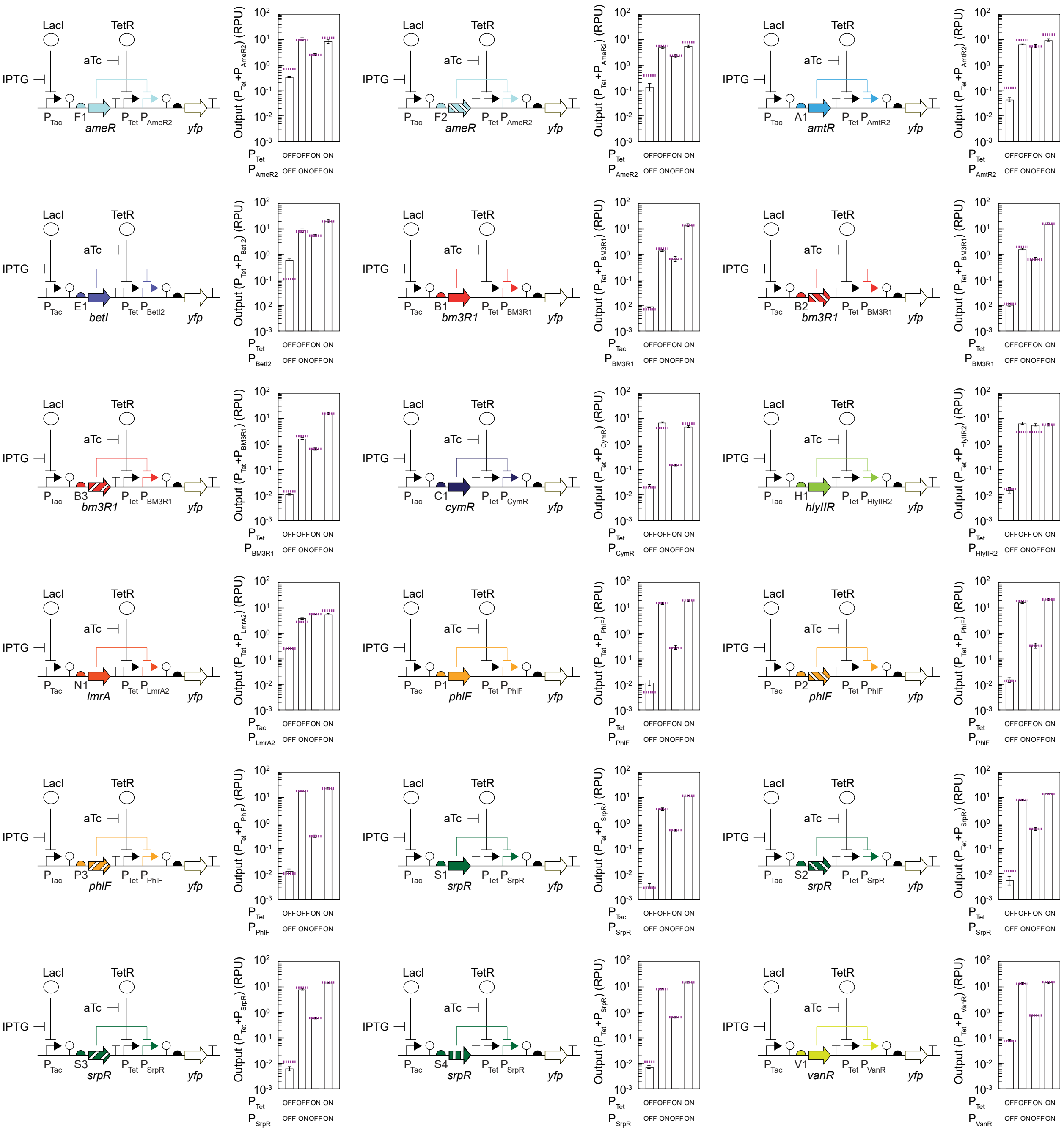

Appendix Figure S6: **Roadblocking assays for the gate output promoters.** The schematics show the gate diagrams, with the output promoter in the downstream position in front of *yfp*. By fitting these data to Appendix Equation S9, the parameters for roadblocking were extracted (Table 1). The non-additive model fit is shown as the purple lines. The OFF states are always the absence of inducer and the ON states are the presence of either 20 ng/mL or 1 mM IPTG. The error bars represent the standard deviation (SD) from three experiments performed on different days.

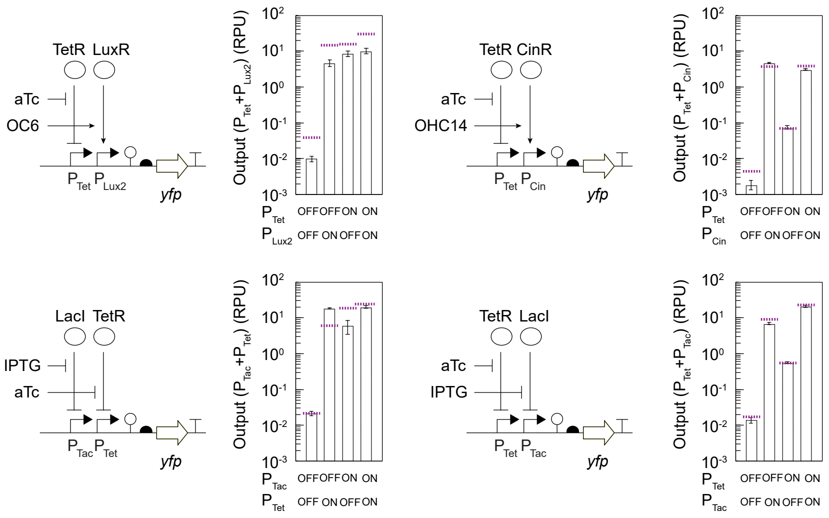

<u>Appendix Figure S7</u>: **Activity of sensor output promoters in tandem.** The schematics show the dual promoters evaluated in series (complete plasmid maps are shown in Appendix Fig S20). The combined output from the promoters is shown as measured in RPU. The purple lines show the predictions from the non-additive model (Appendix Equation S13). The OFF state for the sensors is always the absence of inducer. The ON states are 20 ng/ml aTc (P_{Tet}), 2 µM OC6 (P_{Lux2}), 2 µM OHC14 (P_{Cin}), 1 mM IPTG (P_{Tac}). The error bars represent the standard deviation (SD) from three experiments performed on different days.

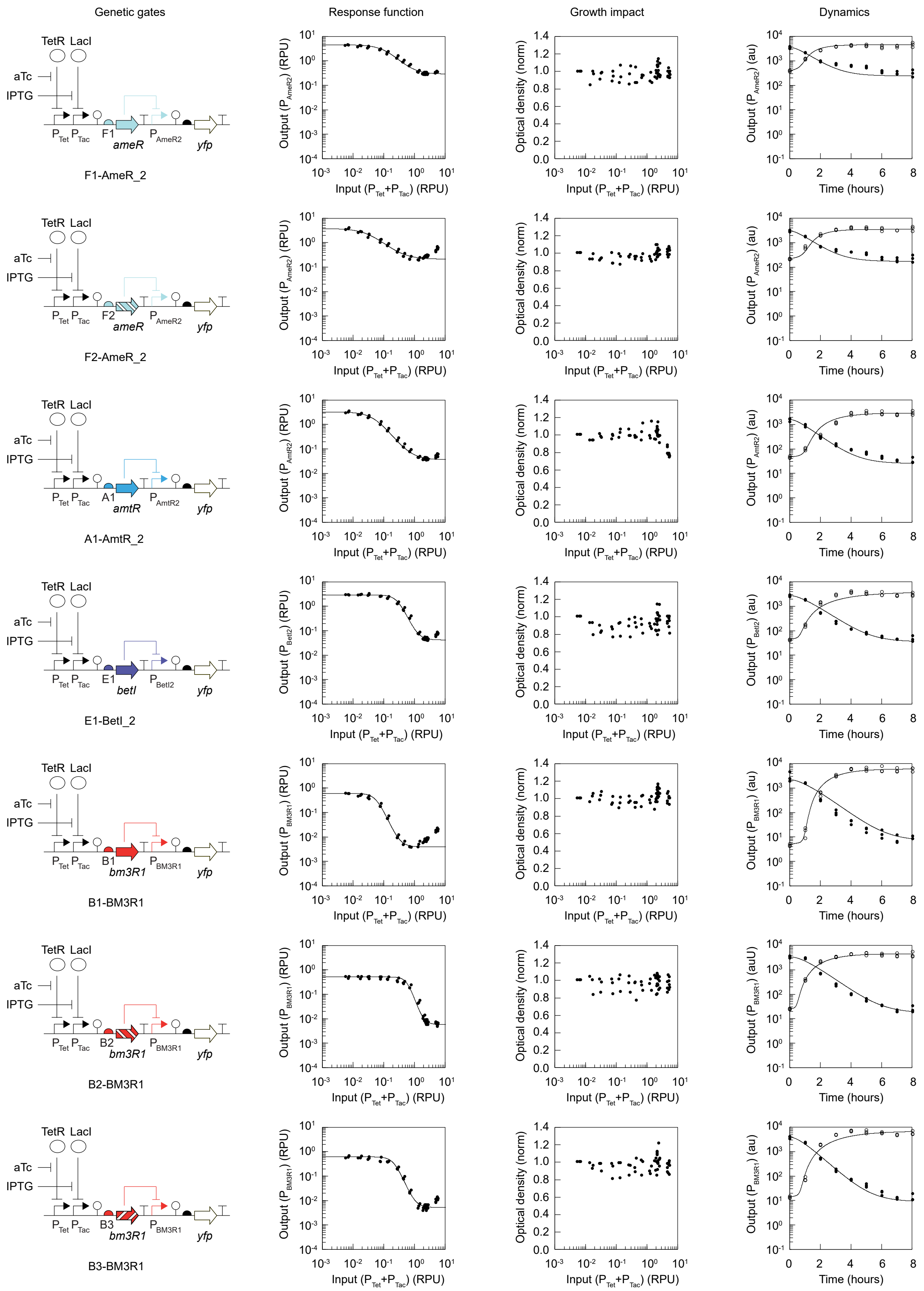

Genetic gates
Response function
Growth impact
Dynamics
TetR LacI
aTc
IPTG
PTet PTac F1 ameR PAmeR2 yfp
F1-AmeR_2
PTet PTac F2 ameR PAmeR2 yfp
F2-AmeR_2
PTet PTac A1 amtR PAmtR2 yfp
A1-AmtR_2
PTet PTac E1 betI PBetI2 yfp
E1-BetI_2
PTet PTac B1 bm3R1 PBM3R1 yfp
B1-BM3R1
PTet PTac B2 bm3R1 PBM3R1 yfp
B2-BM3R1
PTet PTac B3 bm3R1 PBM3R1 yfp
B3-BM3R1
Output (PAmeR2) (RPU)
Output (PAmtR2) (RPU)
Output (PBetI2) (RPU)
Output (PBM3R1) (RPU)
Input (PTet+PTac) (RPU)
Optical density (norm)
Output (PAmeR2) (au)
Output (PAmtR2) (au)
Output (PBetI2) (au)
Output (PBM3R1) (au)
Output (PBM3R1) (auU)
Time (hours)

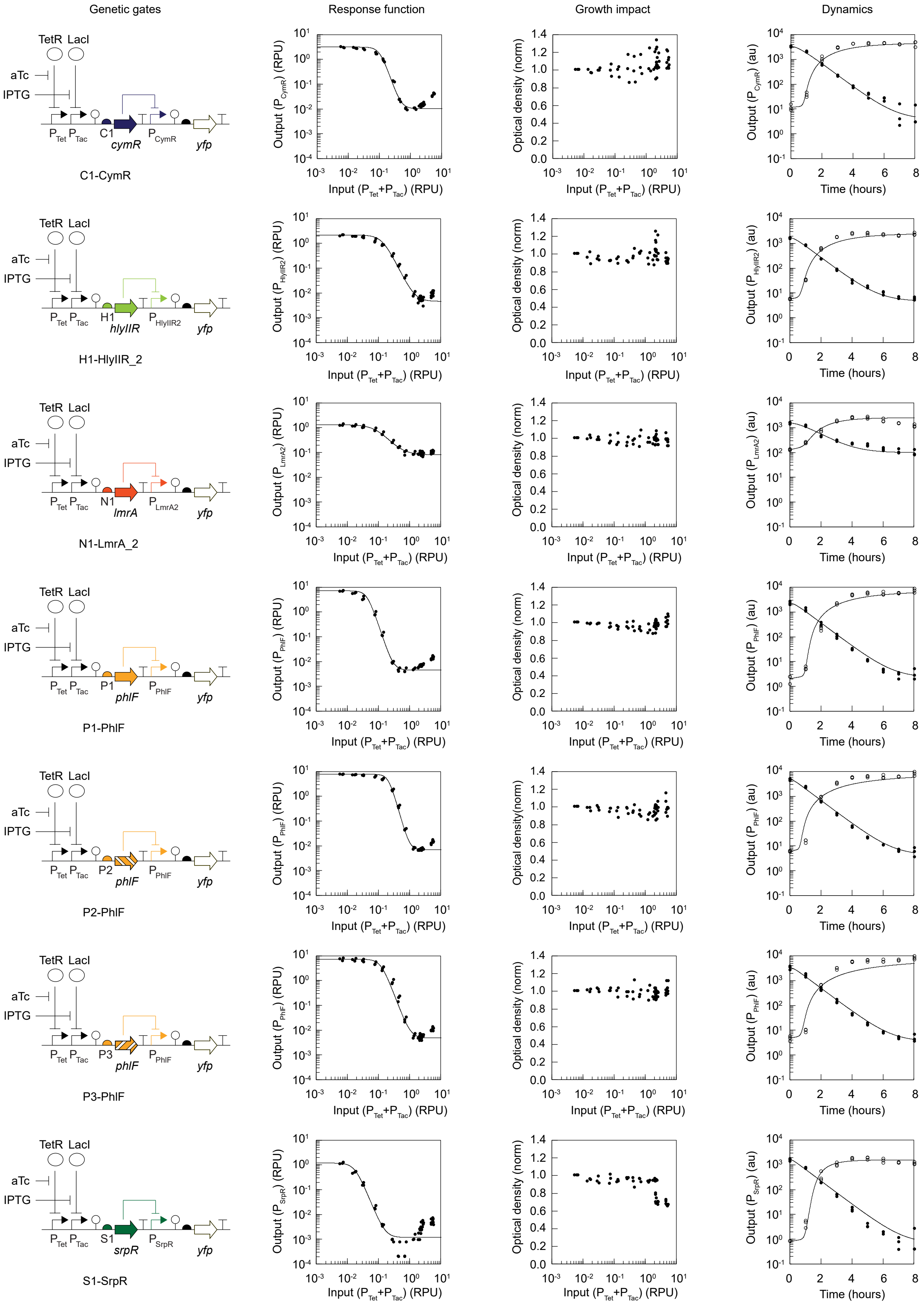
Genetic gates
Response function
Growth impact
Dynamics
TetR LacI
aTc
IPTG
C1-CymR
H1-HlyIIR_2
N1-LmrA_2
P1-PhlF
P2-PhlF
P3-PhlF
S1-SrpR
Input (P_Tet+P_Tac) (RPU)
Optical density (norm)
Time (hours)

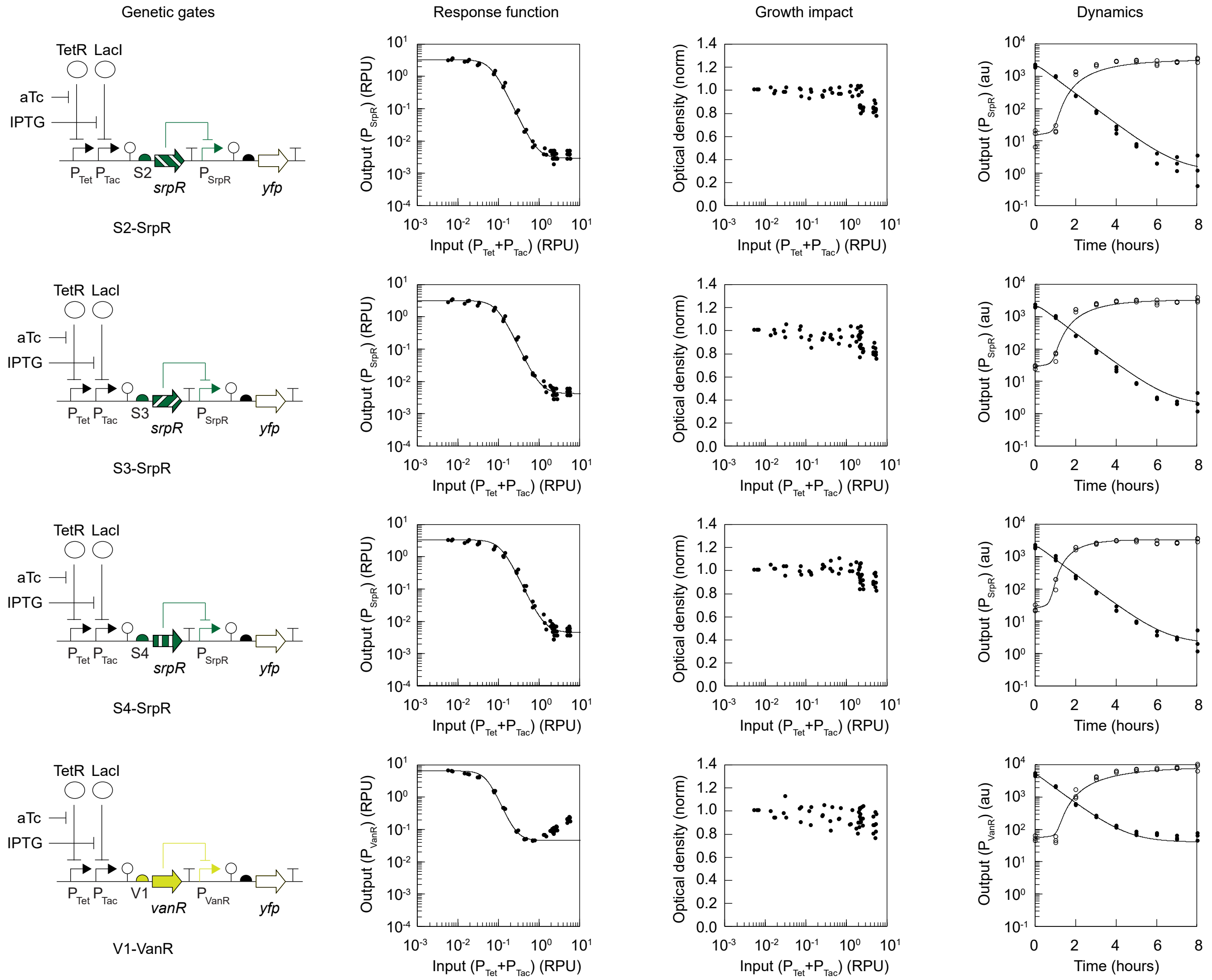

Appendix Figure S8: **Characterization of gates.** Schematics for the gate are shown to the left; the full sequences, parts, and plasmids are provided in Appendix Fig S21, Appendix Table S3 and S4. Data for the response functions are shown. The solid lines are fits to Equation 1 and the resulting fit parameters are shown in Table 1. The growth impact is the OD_{600} normalized by the uninduced sample. The dynamic experiments are fit to numerical solutions of the ODEs in Appendix Equations S20 to S25. The fit parameters are provided in Table 1. Experimental details, including the combinations of inducers used, are provided in the Materials and Methods. The data represent three replicates performed on different days.

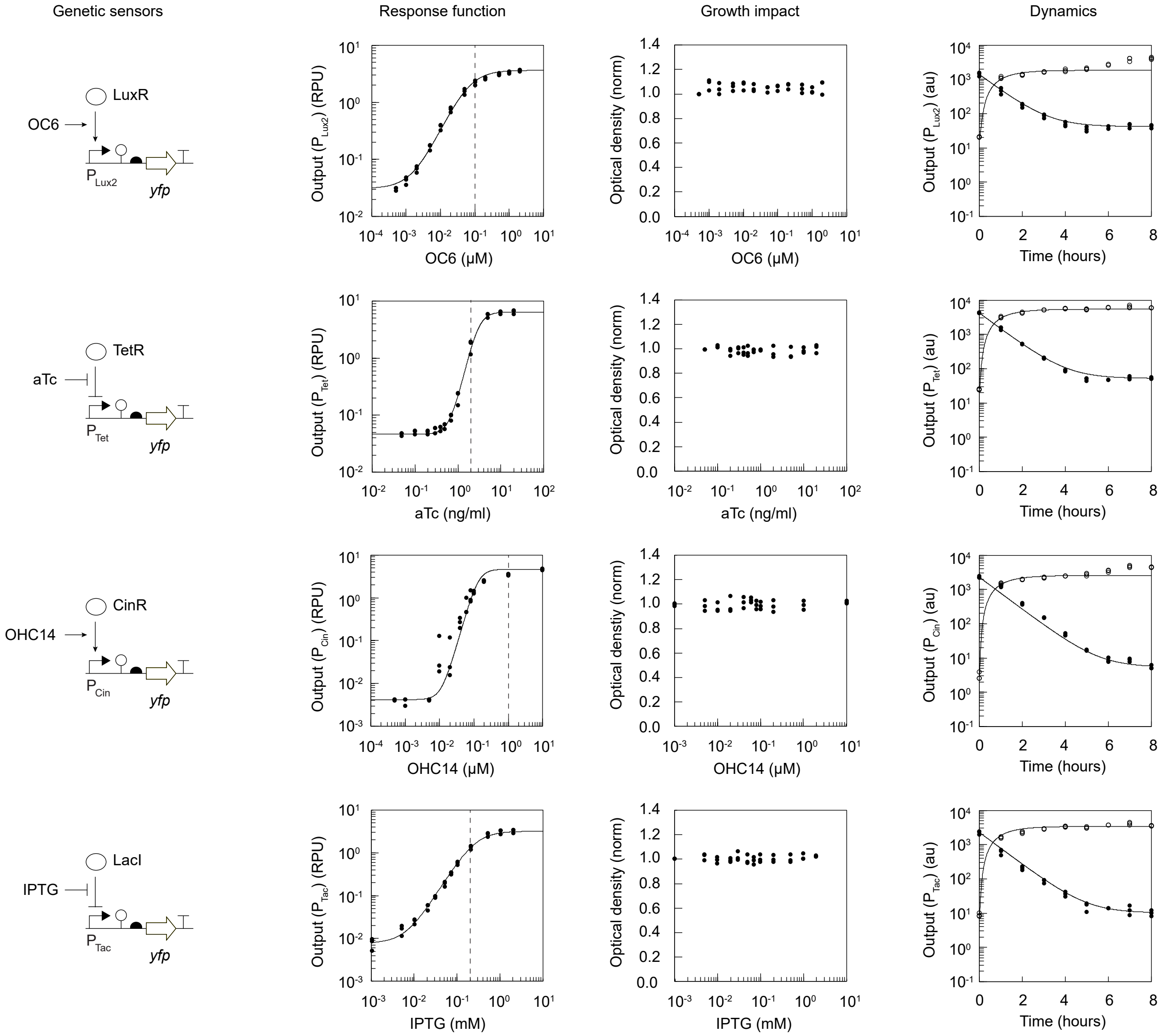

<u>Appendix Figure S9</u>: **Characterization of genetic sensors.** Schematics for the genetic sensors are shown on the left. The complete sensor sequences are provided in Appendix Table S3, genetic parts are in Appendix Table S4, and plasmid maps in Appendix Fig S19. The response functions for the sensors are shown. The data were collected over three days and the line is the fit to Appendix Equation S10. The dashed line indicates the concentration of inducer used for the ON state in Cello (the OFF state is for no inducer). The impact on growth is shown (the data are normalized by the OD_{600} of uninduced sample). The dynamic experiments are fit to numerical solutions of the ODEs in Appendix Equations S18 and S19. The fit parameters are provided in Appendix Table S1. Experiments are described in the Materials and Methods.

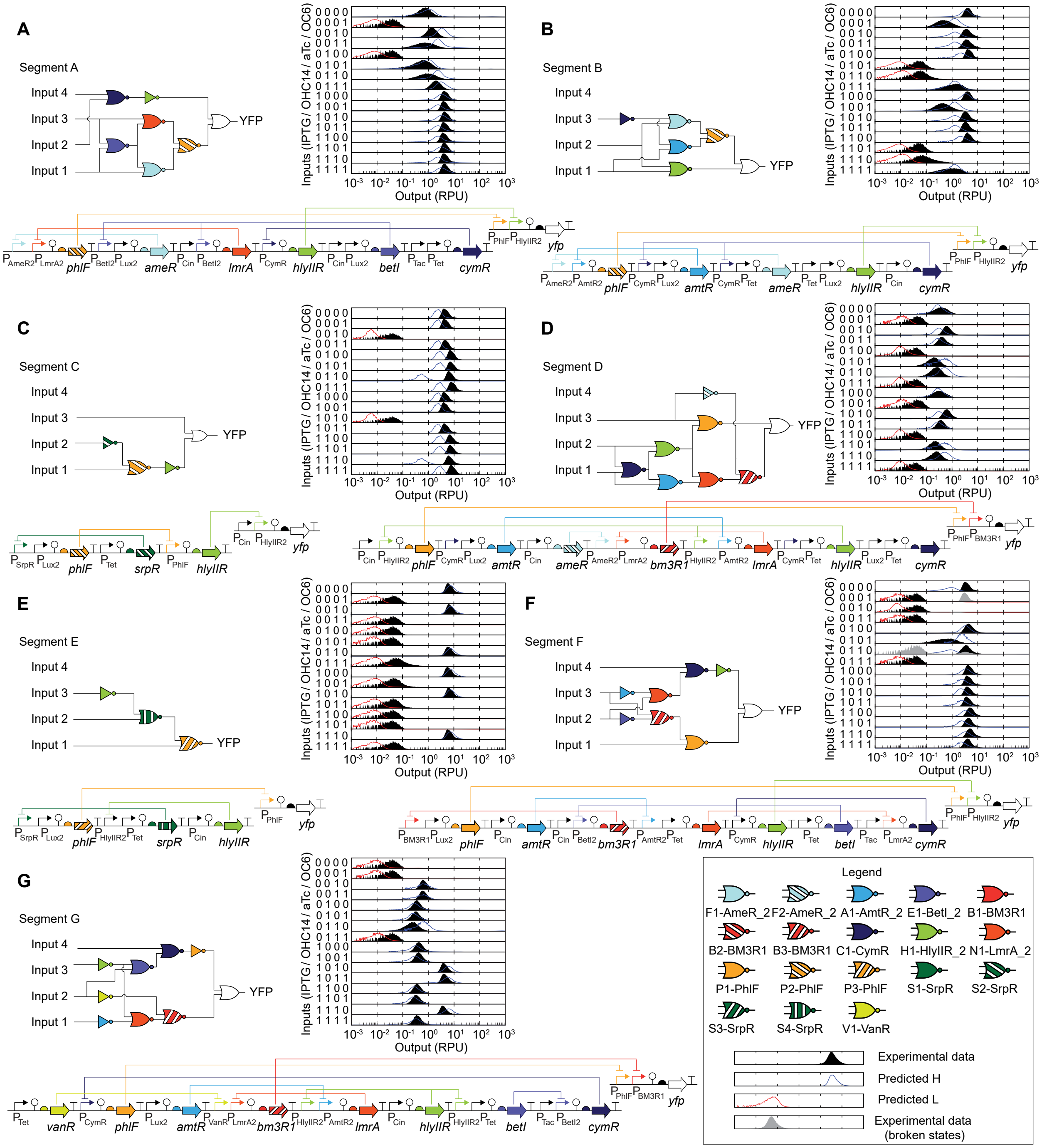

Appendix Figure S10: **Detailed designs and data for the 7-segment circuits.** The complete genetic designs for each circuit are shown. The full sequences are provided in Appendix Table S5 and the plasmid maps are in Appendix Fig S24. The predicted and measured responses are shown for each combination of inputs (0 is absence of inducer and 1 is its presence). The order is (from left to right): 0.2 mM IPTG, 1 µM OHC14, 2 ng/ml aTc, 0.1 µM OC6. The grey distributions for Segment F show the two failed states for circuits recovered after the 88-hour time course (Appendix Fig S13). The median values from the black distributions corresponds to one replicate of three. Additional replicates are shown in Appendix Fig S11 (Input 1: OC6, Input 2: aTc, Input 3: OHC14, Input 4: IPTG).

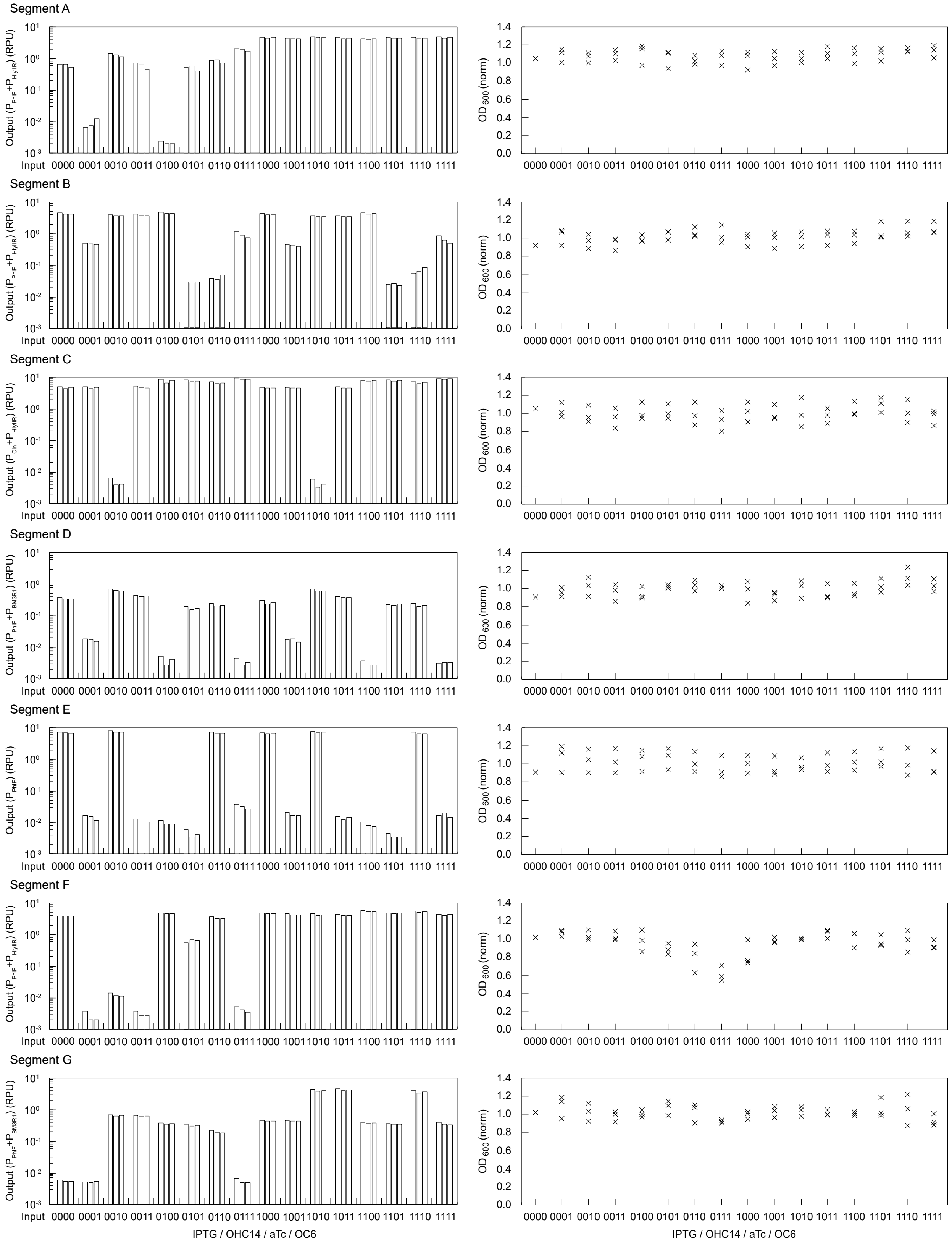
Segment A
Output (P_{PhlF}+P_{HlyIIR}) (RPU)
OD 600 (norm)
Segment B
Output (P_{PhlF}+P_{HlyIIR}) (RPU)
OD 600 (norm)
Segment C
Output (P_{Cin}+P_{HlyIIR}) (RPU)
OD 600 (norm)
Segment D
Output (P_{PhlF}+P_{BM3R1}) (RPU)
OD 600 (norm)
Segment E
Output (P_{PhlF}) (RPU)
OD 600 (norm)
Segment F
Output (P_{PhlF}+P_{HlyIIR}) (RPU)
OD 600 (norm)
Segment G
Output (P_{PhlF}+P_{BM3R1}) (RPU)
OD 600 (norm)
Input 0000 0001 0010 0011 0100 0101 0110 0111 1000 1001 1010 1011 1100 1101 1110 1111
IPTG / OHC14 / aTc / OC6
IPTG / OHC14 / aTc / OC6

Appendix Figure S11: **Response and growth impact of the 7-segment circuits.** The intent of these graphs is to show the reproducibility of the experiments for the cytometry plots in Appendix Fig S10. Inducer concentrations of inputs (P_{Tac} P_{Cin} P_{Tet} P_{Lux2}) were 0.2 mM IPTG, 1 µM OHC14, 2 ng/ml aTc, and 0.1 µM OC6. The bars and data points show the results of three experiments performed on different days. The optical densities are normalized by the uninduced sample. The bars show the median of the fluorescence distribution measured via cytometry, scaled by the RPU standard plasmid (Materials and Methods).

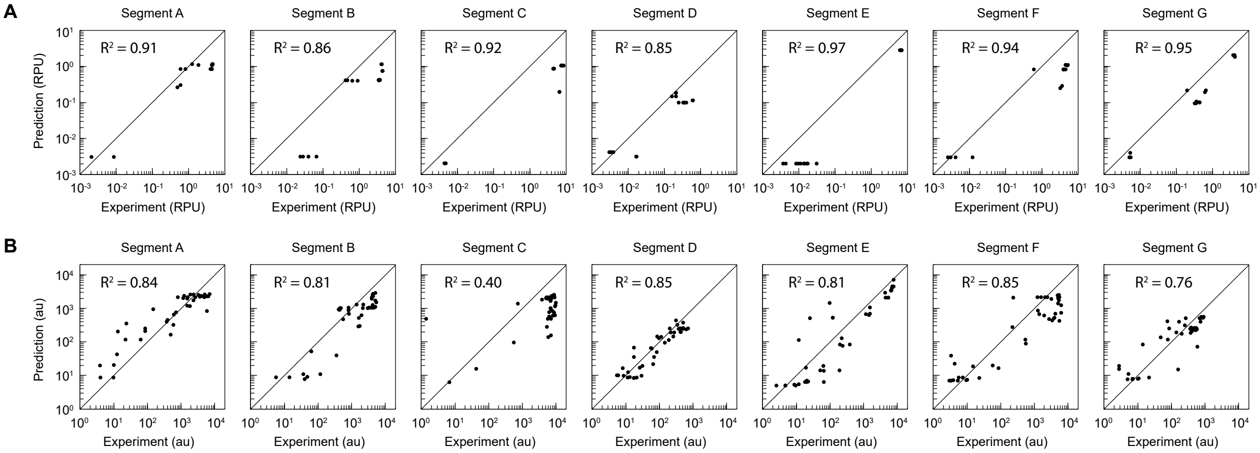

<u>Appendix Figure S12</u>: **Fit of promoter models to response data. (A)** Response of seven-segment circuits shown in Appendix Fig S11 is compared to Cello prediction. **(B)** Response of seven-segment circuits measured every 2 hours (Figure 3B) is compared to model prediction.

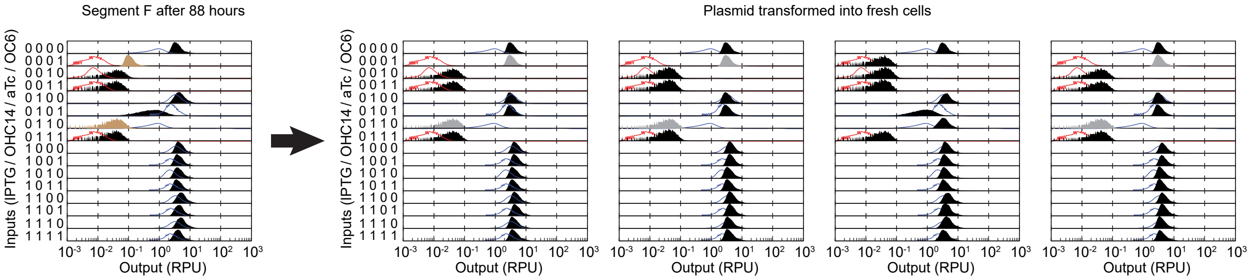

<u>Appendix Figure S13</u>: **Analysis of Segment F (low copy plasmid) after the 88-hour continuous switching experiment.** While the Segment F circuit worked for the states required to display the digit, it failed in some of the 16 possible states (brown distributions, far left). Plasmids for Segment F were recovered after 88-hour time course and retransformed into fresh cells. Four colonies were picked and tested. The predicted and measured responses are shown for each combination of inputs (0 is absence of inducer and 1 is its presence). The order is (from left to right): 0.2 mM IPTG, 1µM OHC14, 2 ng/ml aTc, 0.1 µM OC6. The correct circuit response as predicted by Cello (blue and red) is compared to the measured distributions (black). The grey distributions for Segment F show the two failed states for circuits recovered after the 88-hour time course.

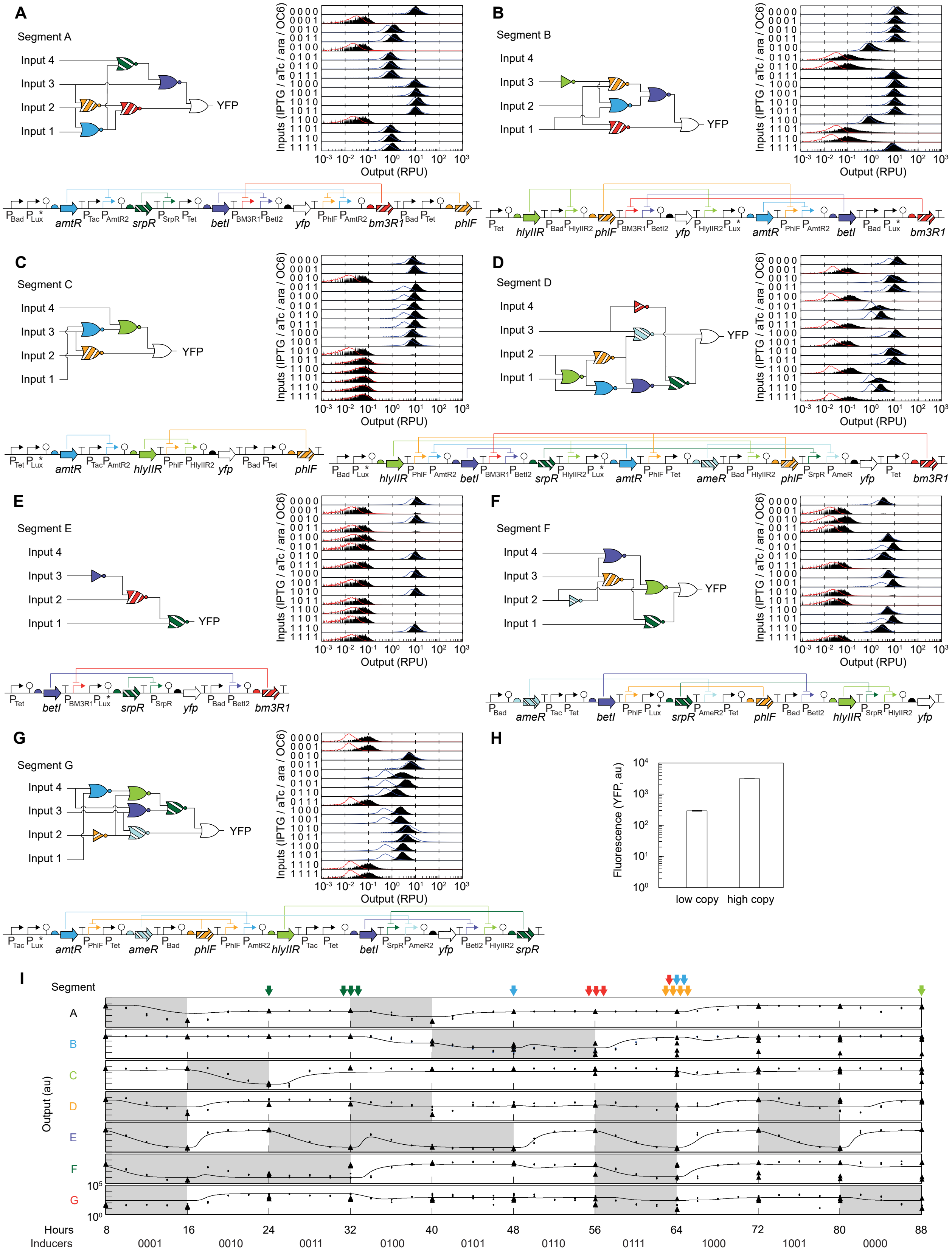
A
Segment A
Input 4
Input 3
Input 2
Input 1
YFP
Inputs (IPTG / aTc / ara / OC6)
Output (RPU)
amtR
srpR
betI
yfp
bm3R1
phlF
B
Segment B
hlyIIR
C
Segment C
D
Segment D
ameR
E
Segment E
F
Segment F
G
Segment G
H
Fluorescence (YFP, au)
low copy
high copy
I
Segment
Output (au)
Hours
Inducers
8
16
24
32
40
48
56
64
72
80
88
0001
0010
0011
0100
0101
0110
0111
1000
1001
0000

__Appendix Figure S14__: **Characterization and stability of the first set of 7-segment designs carried on a higher-copy plasmid.** The gates were originally designed on a higher copy p15a backbone (see text and Materials and Methods for details). These response functions were then used to build a UCF for Cello and the 7-segment circuits were designed. Their DNA was constructed and carried on the same higher copy plasmid. **(A - G)** These circuits performed as expected for all states. The notation, symbols and experimental conditions are identical to those described for Appendix Fig S10. **(H)** The expression of *yfp* from a constitutive promoter is compared for the lower and higher copy plasmids (1 and 2, respectively). Plasmid maps are shown in Appendix Fig S25. **(I)** The time trajectories for the circuits are shown from experiments performed identically to those used to make Fig 3B (Materials and Methods). These experiments were repeated four times on different days and each colored arrow indicates where a circuit failed (defined as producing the wrong output after that time point). The solid line shows the predicted behavior obtained from the set of ODEs described in Appendix Information II, but with parameters that were obtained by fitting equivalent dynamic experiments performed for each gate on the higher-copy plasmid (Materials and Methods).

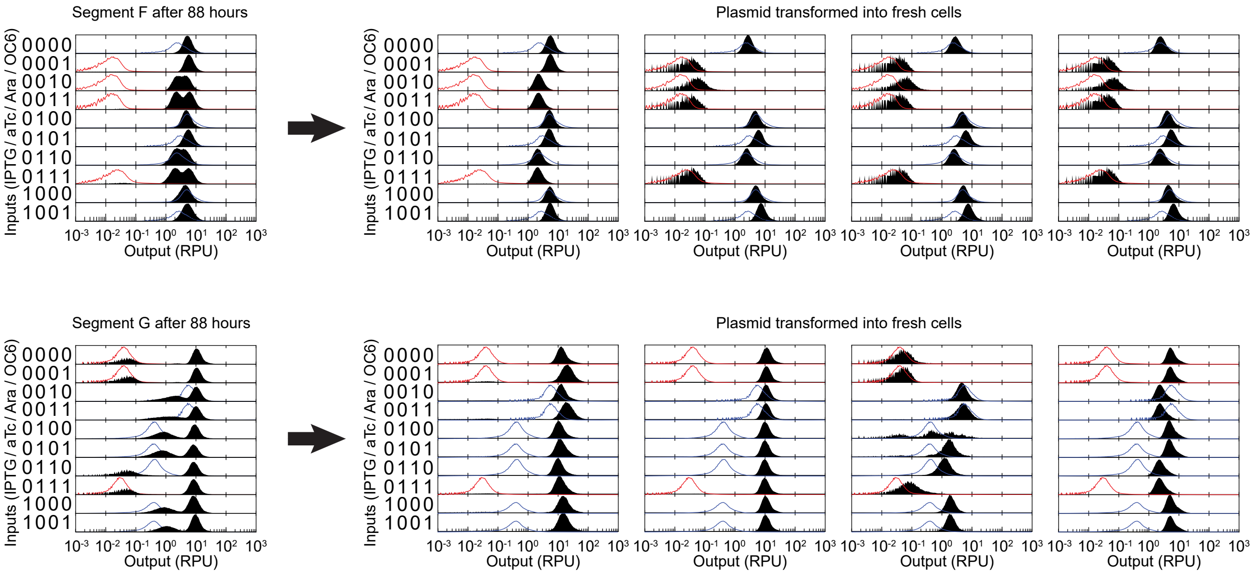

Supplementary Figure S15: **Analysis of Segment F and G (on high copy plasmids) after the 88-hour continuous switching experiment.** Plasmids were recovered from the cells and then transformed into fresh cells. Four colonies were picked and were tested for the circuit response. The correct circuit response as predicted by Cello (blue and red) is compared to the measured distributions (black).

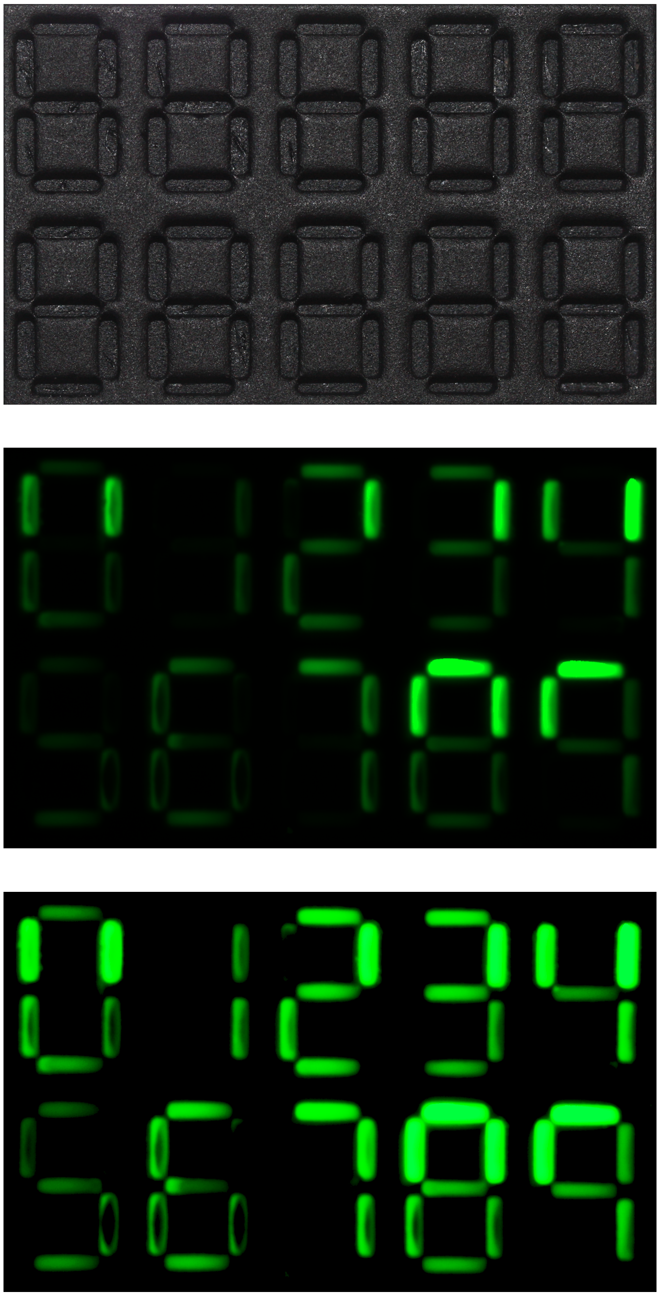

<u>Appendix Figure S16</u>: **3D printed device and seven segment display.** Decimal numbers are shown in Chemidoc using CCD camera with 530/28 filter. Figures taken from Chemidoc under white light (top) and with 530/28 filter (middle) were shown. The image was adjusted using Photoshop CC 2019 (bottom).

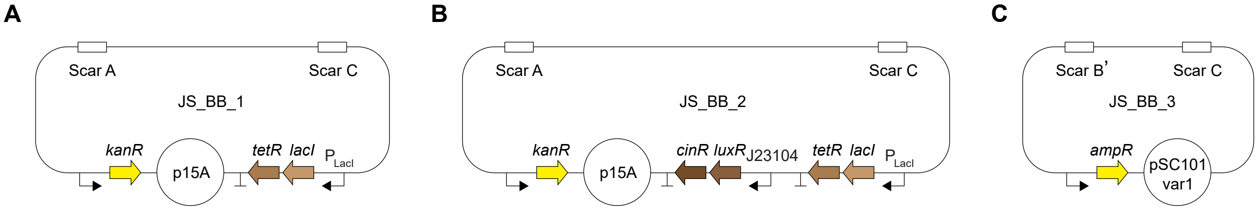

Appendix Figure S17: **Plasmid backbones. (A)** Plasmid backbone used for gate characterization. **(B)** Plasmid backbone used for sensor characterization and seven segment circuits for the second design (low copy). **(C)** Plasmid backbone used for reporters of seven segment circuits for the second design (low copy).

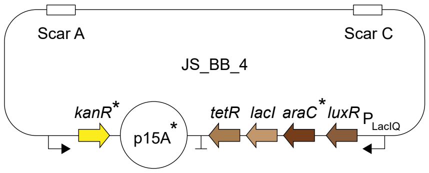

<u>Appendix Figure S18</u>: **High copy plasmid backbone.** This plasmid backbone used for the tandem promoter characterization and the seven segment circuits for the first design (high copy).

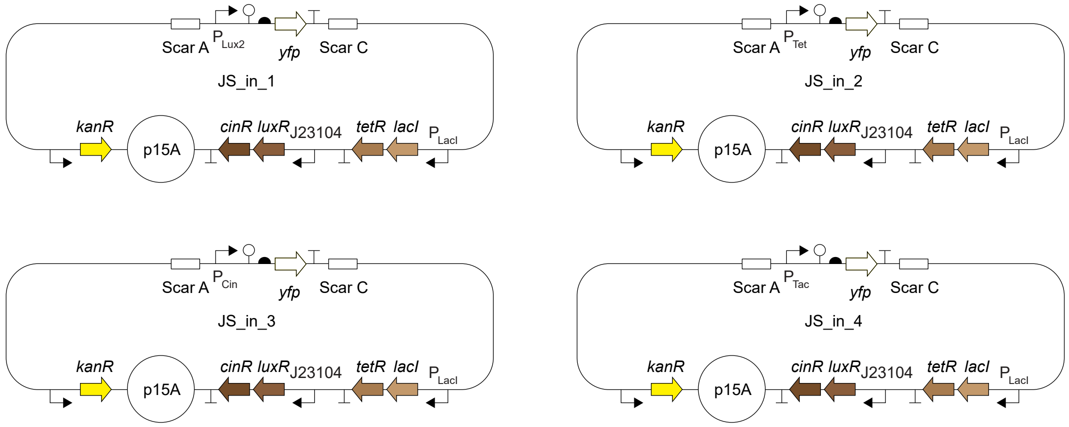

Appendix Figure S19: **Plasmid maps for the sensor characterization.** These plasmids were used for Appendix Fig S9.

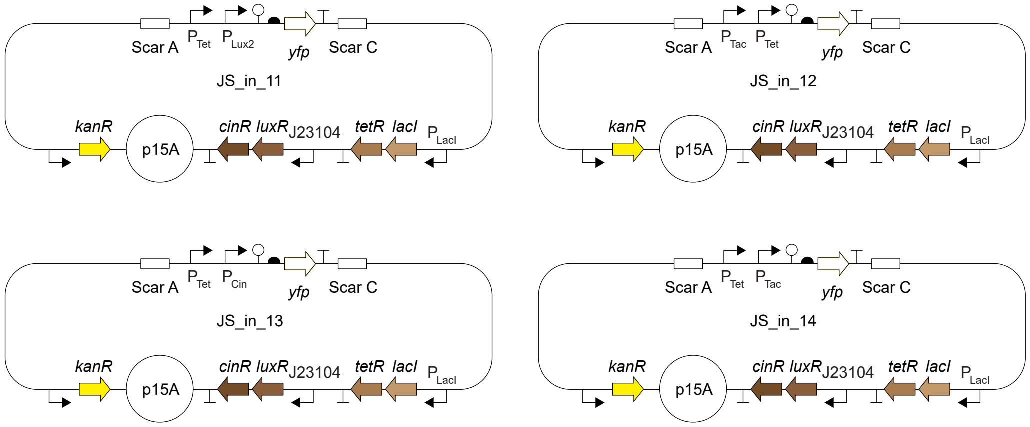

__Appendix Figure S20__: **Plasmid maps for the sensor output tandem promoter characterization.** These plasmids were used for Appendix Fig S7.

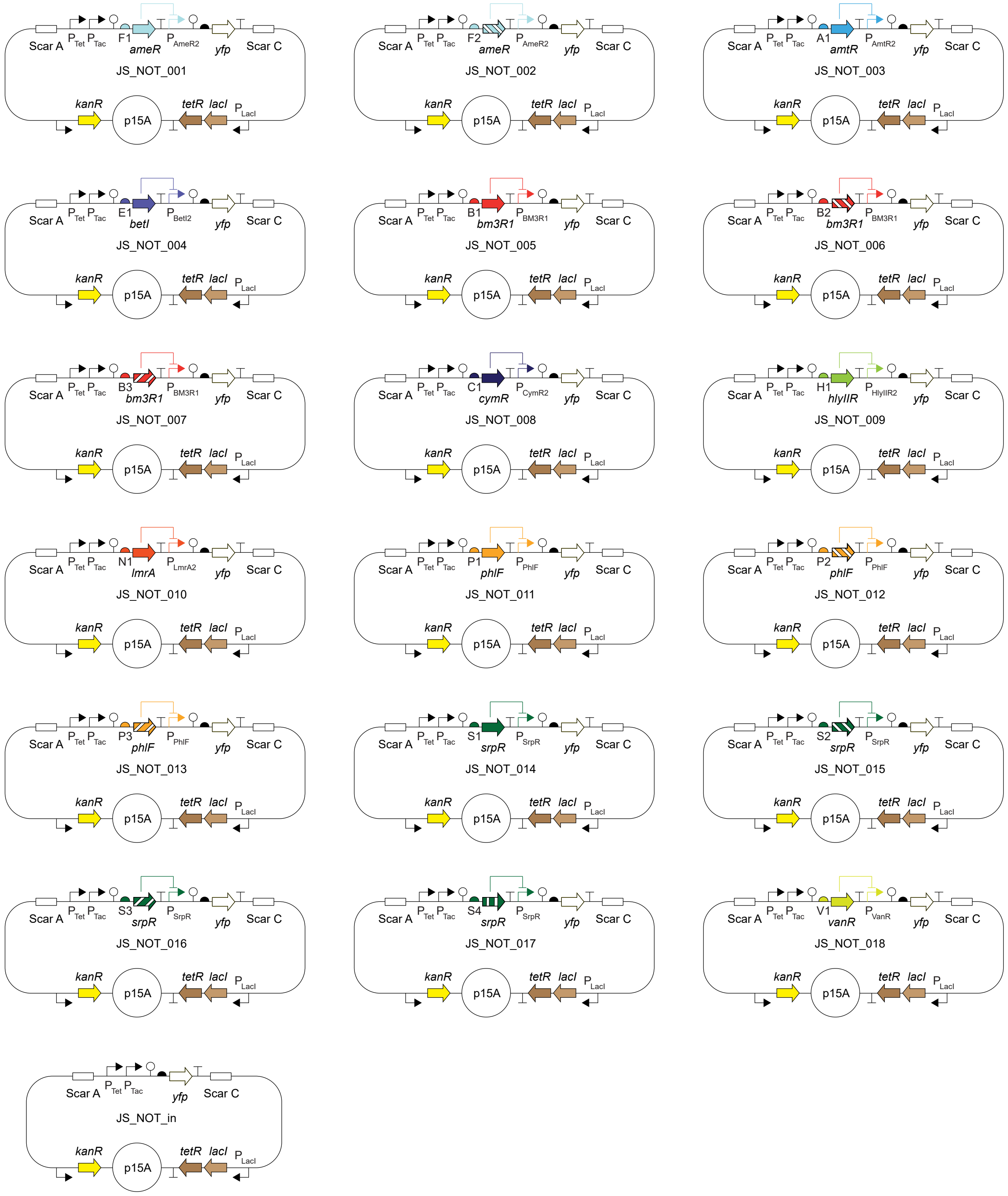

<u>**Appendix Figure S21**</u>: **Plasmid maps for the gate characterization.** These plasmids were used for Appendix Fig S8.

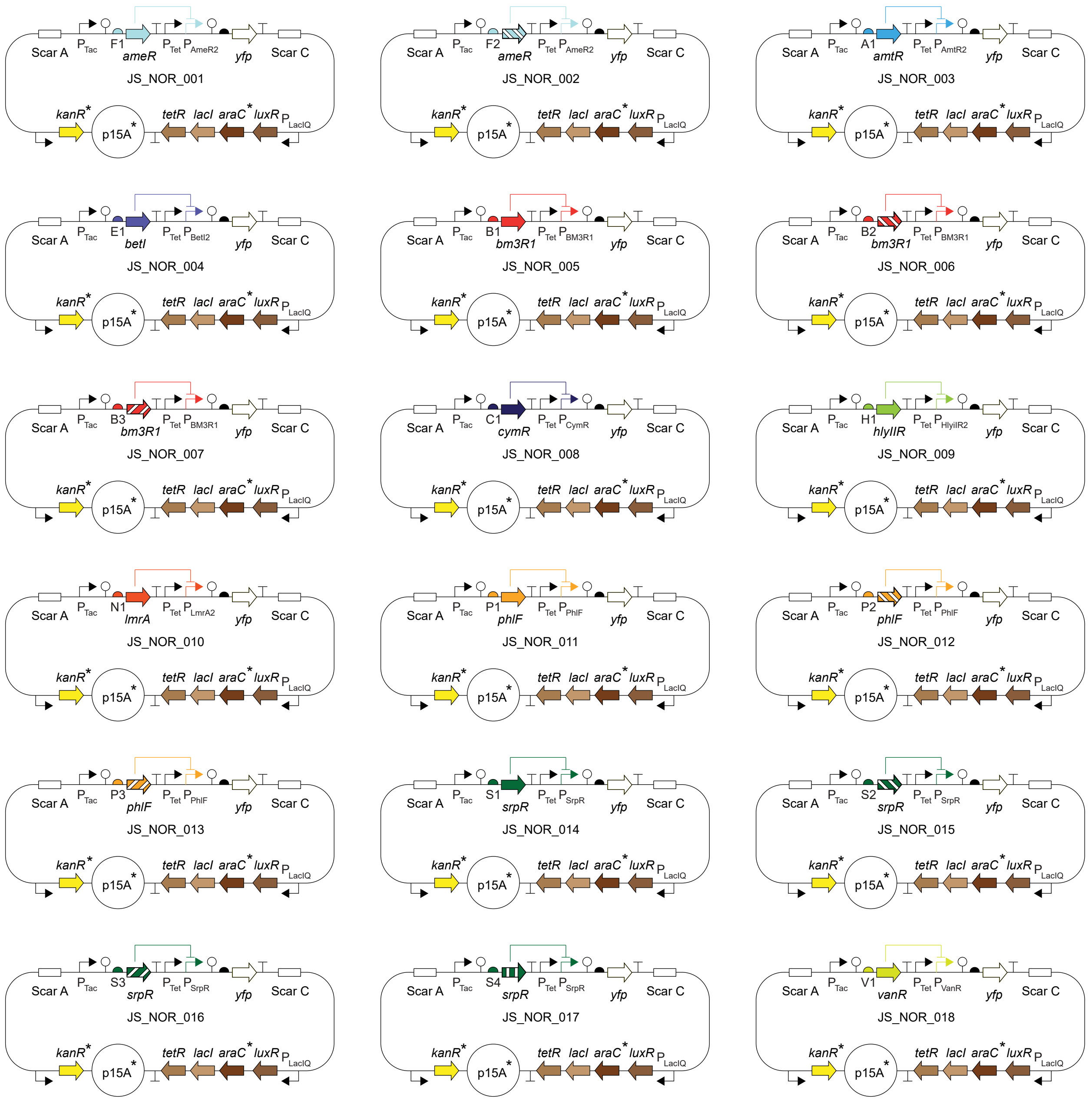

<u>**Appendix Figure S22**</u>: **Plasmid maps for the gate output tandem promoter characterization.** These plasmids were used for Appendix Fig S6.

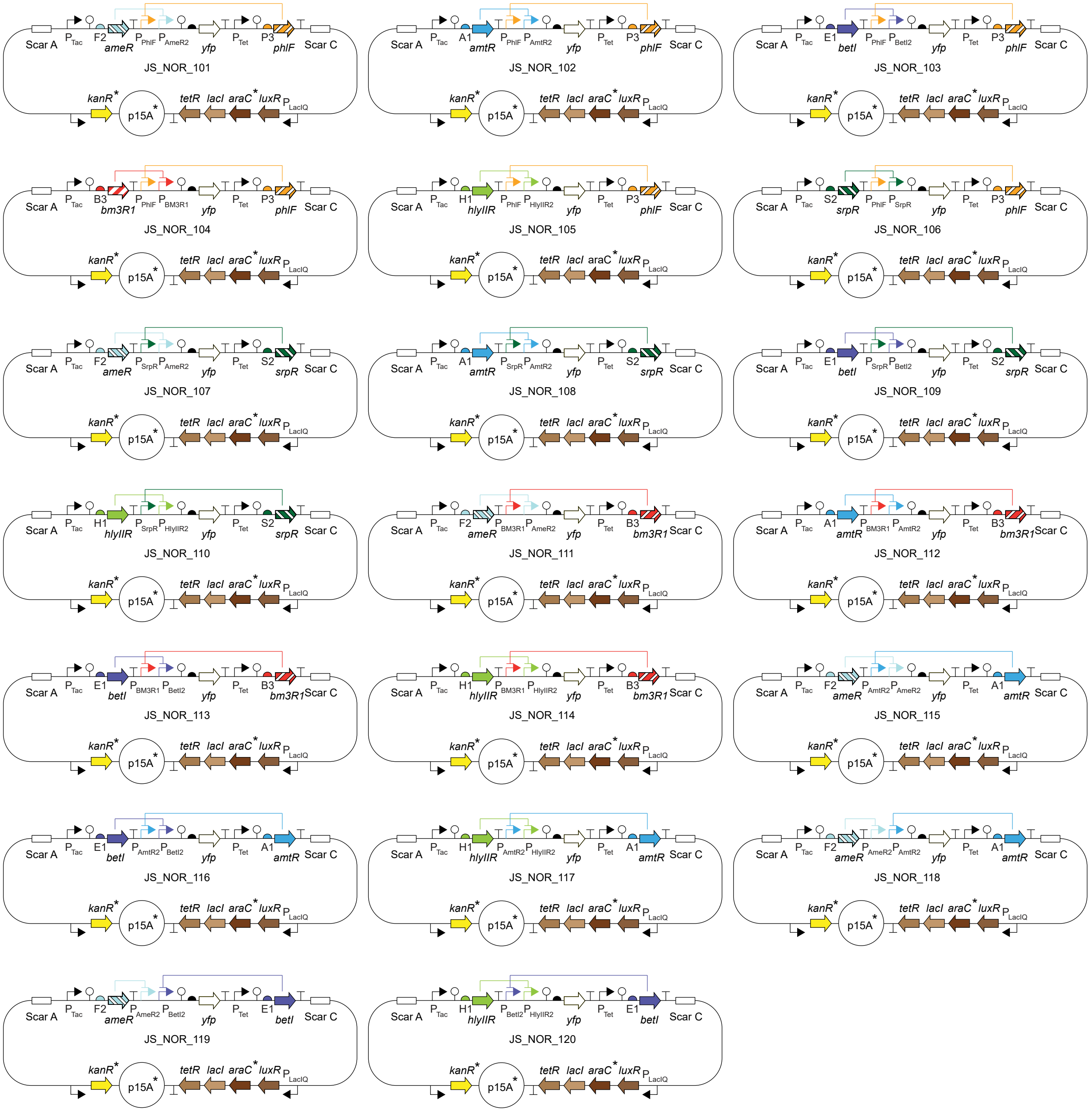

Appendix Figure S23: **Plasmid maps for the tandem promoter evaluation.** These plasmids were used for Fig 1C, 1D and 1E.

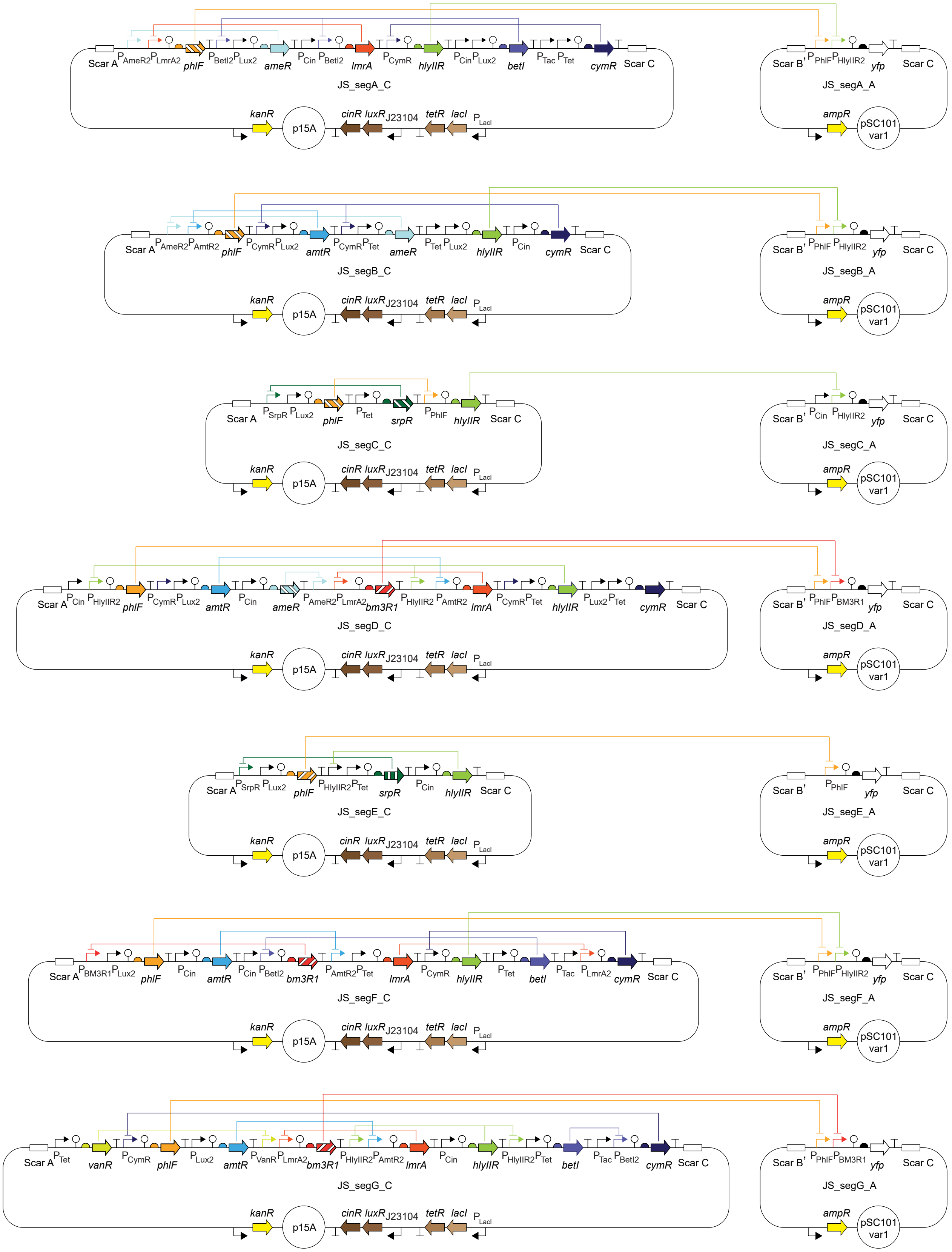

Appendix Figure S24: **Plasmid maps of the 7-segment circuits.** Circuit and reporter plasmids are shown.

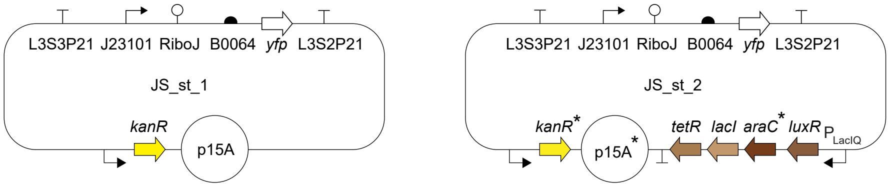

<u>Appendix Figure S25</u>: **Plasmid maps for the YFP expression from a constitutive promoter in two p15A backbones.** Two plasmids maps used for Appendix Fig S14H are shown. Sequences are provided in Appendix Table S4.

Appendix Table S1: Parametrization of sensors

	Response Function				Promoter Interference		Kinetics	
	y_{max}	y_{min}[a]	K	n	Non-specific (α)	Roadblocking (β)[b]	Induction (τ_y^{ON})	Relaxation (τ_y^{OFF})
OHC14(P_{Cin})	4.652	0.004			0.01	0.99	2.70	4.00
OC6 (P_{Lux2})	3.630	0.031			0.80	1.00	2.30	4.00
aTc (P_{Tet})[c]	6.406	0.046			0.69	1.00	3.50	4.00
IPTG (P_{Tac})	3.194	0.008			0.73	0.04	3.50	4.00

a. The sensor output promoter (in RPU) in the presence and absence of the inducer. The ON state was measured for the following concentrations of inducer: 2 µM OHC14, 2 µM OC6, 20 ng/ml aTc, and 1 mM IPTG.
b. The maximum allowed value is one during fitting.
c. To evaluate the P_{Tet} promoter for promoter interference, this promoter in an upstream position from the circuit in Appendix Fig S6 is replaced with P_{Tac}.

Appendix Table S2: List of plasmids used in each figure

Figures	Plasmids
Figure 1 C	JS_NOR_102
Figure 1 D and E	JS_NOR_101 to JS_NOR_120
Figure 1 F and G	JS_NOT_001 to JS_NOT_018
Figure 2	JS_segA to JS_segG
Figure 3	JS_segA to JS_segG
Appendix Figure S2	JS_NOT_002, 003, 004, 009, and 010
Appendix Figure S3	JS_in_1
Appendix Figure S4	JS_NOR_101 to JS_NOR_120
Appendix Figure S6	JS_NOR_001 to JS_NOR_018
Appendix Figure S7	JS_in_11 to JS_in_14
Appendix Figure S8	JS_NOT_001 to JS_NOT_018
Appendix Figure S9	JS_in_1 to JS_in_4
Appendix Figure S10	JS_segA to JS_segG
Appendix Figure S11	JS_segA to JS_segG
Appendix Figure S12	JS_segA to JS_segG
Appendix Figure S13	JS_segF
Appendix Figure S16	JS_segA to JS_segG

Appendix Table S3: Complete annotated sequences of gates and sensors

Part name	Type	DNA sequence[a]
YFP	Reporter	AGCTGTCACCGGATGTGCTTTCCGGTCTGATGAGTCCGTGAGGACGAAACAGCCTCTACAAATAATTTTGTTTAATACTAGAGAAAGAGGGGAAAT<br>ACTAGATGGTGAGCAAGGGCGAGGAGCTGTTCACCGGGGTGGTGCCCATCCTGGTCGAGCTGGACGGCGACGTAAACGGCCACAAGTTCAGCGTGT<br>CCGGCGAGGGCGAGGGCGATGCCACCTACGGCAAGCTGACCCTGAAGTTCATCTGCACCACAGGCAAGCTGCCCGTGCCCTGGCCCACCCTCGTGA<br>CCACCTTCGGCTACGGCCTGCAATGCTTCGCCCGCTACCCCGACCACATGAAGCTGCACGACTTCTTCAAGTCCGCCATGCCCGAAGGCTACGTCC<br>AGGAGCGCACCATCTTCTTCAAGGACGACGGCAACTACAAGACCCGCGCCGAGGTGAAGTTCGAGGGCGACACCCTGGTGAACCGCATCGAGCTGA<br>AGGGCATCGACTTCAAGGAGGACGGCAACATCCTGGGGCACAAGCTGGAGTACAACTACAACAGCCACAACGTCTATATCATGGCCGACAAGCAGA<br>AGAACGGCATCAAGGTGAACTTCAAGATCCGCCACAACATCGAGGACGGCAGCGTGCAGCTCGCCGACCACTACCAGCAGAACACCCCAATCGGCG<br>ACGGCCCCGTGCTGCTGCCCGACAACCACTACCTTAGCTACCAGTCCGCCCTGAGCAAAGACCCCAACGAGAAGCGCGATCACATGGTCCTGCTGG<br>AGTTCGTGACCGCCGCCGGGATCACTCTCGGCATGGACGAGCTGTACAAGTAACTCGGTACCAAATTCCAGAAAAGAGGCCTCCCGAAAGGGGGGC<br>CTTTTTTCGTTTTGGTCC
F1-AmeR_2	Gate	AGGGGTCAGTTGATGTGCTTTCAACTCTGATGAGTCAGTGATGACGAAACCCCCTCTACAAATAATTTTGTTTAACTATGGACTATGTTTTCACAT<br>ACGAGGGGGATTAGATGAACAAAACCATTGATCAGGTGCGTAAAGGTGATCGTAAAAGCGATCTGCCGGTTCGTCGTCGTCCGCGTCGTAGTGCCG<br>AAGAAACCCGTCGTGATATTCTGGCAAAAGCCGAAGAACTGTTTCGTGAACGTGGTTTTAATGCAGTTGCCATTGCAGATATTGCAAGCGCACTGA<br>ATATGAGTCCGGCAAATGTGTTTAAACATTTTAGCAGCAAAAACGCACTGGTTGATGCAATTGGTTTTGGTCAGATTGGTGTTTTTGAACGTCAGA<br>TTTGTCCGCTGGATAAAAGCCATGCACCGCTGGATCGTCTGCGTCATCTGGCACGTAATCTGATGGAACAGCATCATCAGGATCATTTCAAACACA<br>TACGGGTTTTTATTCAGATCCTGATGACCGCCAAACAGGATATGAAATGTGGCGATTATTACAAAAGCGTGATTGCAAAACTGCTGGCCGAAATTA<br>TTCGTGATGGTGTTGAAGCAGGTCTGTATATTGCAACCGATATTCCGGTTCTGGCAGAAACCGTTCTGCATGCACTGACCAGCGTTATTCATCCGG<br>TTCTGATTGCACAAGAAGATATTGGTAATCTGGCAACCCGTTGTGATCAGCTGGTTGATCTGATTGATGCAGGTCTGCGTAATCCGCTGGCAAAAT<br>AACCAATTATTGAACACCCTAACGGGTGTTTTTTTTTTTTTGGTCTCCCTCGTCACTAGAGGGCGATAGTGACAAACTTGACAACTCATCACTTCC<br>TACGTAGGCTGCTAGC
F2-AmeR_2	Gate	AGGGGTCAGTTGATGTGCTTTCAACTCTGATGAGTCAGTGATGACGAAACCCCCTCTACAAATAATTTTGTTTAATACGCTATGGACTATGTTTCA<br>AACAGGAGCTAATAGATGAACAAAACCATTGATCAGGTGCGTAAAGGTGATCGTAAAAGCGATCTGCCGGTTCGTCGTCGTCCGCGTCGTAGTGCC<br>GAAGAAACCCGTCGTGATATTCTGGCAAAAGCCGAAGAACTGTTTCGTGAACGTGGTTTTAATGCAGTTGCCATTGCAGATATTGCAAGCGCACTG<br>AATATGAGTCCGGCAAATGTGTTTAAACATTTTAGCAGCAAAAACGCACTGGTTGATGCAATTGGTTTTGGTCAGATTGGTGTTTTTGAACGTCAG<br>ATTTGTCCGCTGGATAAAAGCCATGCACCGCTGGATCGTCTGCGTCATCTGGCACGTAATCTGATGGAACAGCATCATCAGGATCATTTCAAACAC<br>ATACGGGTTTTTATTCAGATCCTGATGACCGCCAAACAGGATATGAAATGTGGCGATTATTACAAAAGCGTGATTGCAAAACTGCTGGCCGAAATT<br>ATTCGTGATGGTGTTGAAGCAGGTCTGTATATTGCAACCGATATTCCGGTTCTGGCAGAAACCGTTCTGCATGCACTGACCAGCGTTATTCATCCG<br>GTTCTGATTGCACAAGAAGATATTGGTAATCTGGCAACCCGTTGTGATCAGCTGGTTGATCTGATTGATGCAGGTCTGCGTAATCCGCTGGCAAAA<br>TAACCAATTATTGAACACCCTAACGGGTGTTTTTTTTTTTTTGGTCTCCCTCGTCACTAGAGGGCGATAGTGACAAACTTGACAACTCATCACTTC<br>CTACGTAGGCTGCTAGC
A1-AmtR_2	Gate	AGGGTGTCTCAAGGTGCGTACCTTGACTGATGAGTCCGAAAGGACGAAACACCCCTCTACAAATAATTTTGTTTAAAATGTTCCCTAATAATCAGC<br>AAAGAGGTTACTAGATGGCAGGCGCAGTTGGTCGTCCGCGTCGTAGTGCACCGCGTCGTGCAGGTAAAAATCCGCGTGAAGAAATTCTGGATGCAA<br>GCGCAGAACTGTTTACCCGTCAGGGTTTTGCAACCACCAGTACCCATCAGATTGCAGATGCAGTTGGTATTCGTCAGGCAAGCCTGTATTATCATT<br>TTCCGAGCAAAACCGAAATCTTTCTGACCCTGCTGAAAAGCACCGTTGAACCGAGCACCGTTCTGGCAGAAGATCTGAGCACCCTGGATGCAGGTC<br>CGGAAATGCGTCTGTGGGCAATTGTTGCAAGCGAAGTTCGTCTGCTGCTGAGCACCAAATGGAATGTTGGTCGTCTGTATCAGCTGCCGATTGTTG<br>GTAGCGAAGAATTTGCAGAATATCATAGCCAGCGTGAAGCACTGACCAATGTTTTTCGTGATCTGGCAACCGAAATTGTTGGTGATGATCCGCGTG<br>CAGAACTGCCGTTTCATATTACCATGAGCGTTATTGAAATGCGTCGCAATGATGGTAAAATTCCGAGTCCGCTGAGCGCAGATAGCCTGCCGGAAA<br>CCGCAATTATGCTGGCAGATGCAAGCCTGGCAGTTCTGGGTGCACCGCTGCCTGCAGATCGTGTTGAAAAAACCCTGGAACTGATTAAACAGGCAG<br>ATGCAAAATAACTCGGTACCAAAGACGAACAATAAGACGCTGAAAAGCGTCTTTTTTCGTTTTGGTCCCTTGTCCAACCAAATGATTCGTTACCCT<br>TTGACAGTTTCTATCGATCTATAGATAATGCTAGC
E1-BetI_2	Gate	AGAAGTCAATTAATGTGCTTTTAATTCTGATGAGTCGGTGACGACGAAACTTCCTCTACAAATAATTTTGTTTAACCCCCCGAGGAGTAGCACATG<br>CCGAAACTGGGTATGCAGAGCATTCGTCGTCGTCAGCTGATTGATGCAACCCTGGAAGCAATTAATGAAGTTGGTATGCATGATGCAACCATTGCA<br>CAGATTGCACGTCGTGCCGGTGTTAGCACCGGTATTATTAGCCATTATTTCCGCGATAAAAACGGTCTGCTGGAAGCAACCATGCGTGATATTACC<br>AGCCAGCTGCGTGATGCAGTTCTGAATCGTCTGCATGCACTGCCGCAGGGTAGCGCAGAACAGCGTCTGCAGGCAATTGTTGGTGGTAATTTTGAT<br>GAAACCCAGGTTAGCAGCGCAGCAATGAAAGCATGGCTGGCATTTTGGGCAAGCAGCATGCATCAGCCGATGCTGTATCGTCTGCAGCAGGTTAGC<br>AGTCGTCGTCTGCTGAGCAATCTGGTTAGCGAATTTCGTCGTGAACTGCCTCGTGAACAGGCACAAGAGGCAGGTTATGGTCTGGCAGCACTGATT<br>GATGGTCTGTGGCTGCGTGCAGCACTGAGCGGTAAACCGCTGGATAAAACCCGTGCAAATAGCCTGACCCGTCATTTTATCACCCAGCATCTGCCG<br>ACCGATTAACCAATTATTGAACACCCTTCGGGGTGTTTTTTTGTTTCTGGTCTCCCAGCGCGGGTGAGAGGGATTCGTTACCAATAGACAATTGAT<br>TGGACGTTCAATATAATGCTAGC
B1-BM3R1	Gate	AGACTGTCGCCGGATGTGTATCCGACCTGACGATGGCCCAAAAGGGCCGAAACAGTCCTCTACAAATAATTTTGTTTAACTATGGACTATGTTTTA<br>ACTACTAGATGGAAAGCACCCCGACCAAACAGAAAGCAATTTTTAGCGCAAGCCTGCTGCTGTTTGCAGAACGTGGTTTTGATGCAACCACCATGC<br>CGATGATTGCAGAAAATGCAAAAGTTGGTGCAGGCACCATTTATCGCTATTTCAAAAACAAAGAAAGCCTGGTGAACGAACTGTTTCAGCAGCATG<br>TTAATGAATTTCTGCAGTGTATTGAAAGCGGTCTGGCAAATGAACGTGATGGTTATCGTGATGGCTTTCATCACATTTTTGAAGGTATGGTGACCT<br>TTACCAAAAATCATCCGCGTGCACTGGGTTTTATCAAAACCCATAGCCAGGGCACCTTTCTGACCGAAGAAAGCCGTCTGGCATATCAGAAACTGG<br>TTGAATTTGTGTGCACCTTTTTTCGTGAAGGTCAGAAACAGGGTGTGATTCGTAATCTGCCGGAAAATGCACTGATTGCAATTCTGTTTGGCAGCT<br>TTATGGAAGTGTATGAAATGATCGAGAACGATTATCTGAGCCTGACCGATGAACTGCTGACCGGTGTTGAAGAAAGCCTGTGGGCAGCACTGAGCC

GTCAGAGCTAACTCGGTACCAAATTCCAGAAAAGAGACGCTTTCGAGCGTCTTTTTTCGTTTTGGTCCAATCCGCGTGATAGGTCTGATTCGTTAC
CAATTGACGGAATGAACGTTCATTCCGATAATGCTAGC

B2-BM3R1 Gate

AGACTGTCGCCGGATGTGTATCCGACCTGACGATGGCCCAAAAGGGCCGAAACAGTCCTCTACAAATAATTTTGTTTAACTATGGACTATGTTTTT
CAAAGACGAAAAACTACTAGATGGAAAGCACCCCGACCAAACAGAAAGCAATTTTTAGCGCAAGCCTGCTGCTGTTTGCAGAACGTGGTTTTGATG
CAACCACCATGCCGATGATTGCAGAAAATGCAAAAGTTGGTGCAGGCACCATTTATCGCTATTTCAAAAACAAAGAAAGCCTGGTGAACGAACTGT
TTCAGCAGCATGTTAATGAATTTCTGCAGTGTATTGAAAGCGGTCTGGCAAATGAACGTGATGGTTATCGTGATGGCTTTCATCACATTTTTGAAG
GTATGGTGACCTTTACCAAAAATCATCCGCGTGCACTGGGTTTTATCAAAACCCATAGCCAGGGCACCTTTCTGACCGAAGAAAGCCGTCTGGCAT
ATCAGAAACTGGTTGAATTTGTGTGCACCTTTTTTCGTGAAGGTCAGAAACAGGGTGTGATTCGTAATCTGCCGGAAAATGCACTGATTGCAATTC
TGTTTGGCAGCTTTATGGAAGTGTATGAAATGATCGAGAACGATTATCTGAGCCTGACCGATGAACTGCTGACCGGTGTTGAAGAAAGCCTGTGGG
CAGCACTGAGCCGTCAGAGCTAACTCGGTACCAAATTCCAGAAAAGAGACGCTTTCGAGCGTCTTTTTTCGTTTTGGTCCAATCCGCGTGATAGGT
CTGATTCGTTACCAATTGACGGAATGAACGTTCATTCCGATAATGCTAGC

B3-BM3R1 Gate

AGACTGTCGCCGGATGTGTATCCGACCTGACGATGGCCCAAAAGGGCCGAAACAGTCCTCTACAAATAATTTTGTTTAACCAAACGAGGCCGGGAG
GATGGAAAGCACCCCGACCAAACAGAAAGCAATTTTTAGCGCAAGCCTGCTGCTGTTTGCAGAACGTGGTTTTGATGCAACCACCATGCCGATGAT
TGCAGAAAATGCAAAAGTTGGTGCAGGCACCATTTATCGCTATTTCAAAAACAAAGAAAGCCTGGTGAACGAACTGTTTCAGCAGCATGTTAATGA
ATTTCTGCAGTGTATTGAAAGCGGTCTGGCAAATGAACGTGATGGTTATCGTGATGGCTTTCATCACATTTTTGAAGGTATGGTGACCTTTACCAA
AAATCATCCGCGTGCACTGGGTTTTATCAAAACCCATAGCCAGGGCACCTTTCTGACCGAAGAAAGCCGTCTGGCATATCAGAAACTGGTTGAATT
TGTGTGCACCTTTTTTCGTGAAGGTCAGAAACAGGGTGTGATTCGTAATCTGCCGGAAAATGCACTGATTGCAATTCTGTTTGGCAGCTTTATGGA
AGTGTATGAAATGATCGAGAACGATTATCTGAGCCTGACCGATGAACTGCTGACCGGTGTTGAAGAAAGCCTGTGGGCAGCACTGAGCCGTCAGAG
CTAACTCGGTACCAAATTCCAGAAAAGAGACGCTTTCGAGCGTCTTTTTTCGTTTTGGTCCAATCCGCGTGATAGGTCTGATTCGTTACCAATTGA
CGGAATGAACGTTCATTCCGATAATGCTAGC

C1-CymR Gate

AGTACGTCTGAGCGTGATACCCGCTCACTGAAGATGGCCCGGTAGGGCCGAAACGTACCTCTACAAATAATTTTGTTTAAGACTTAAACGATTGGC
CGACCGCATGAGCCCGAAACGTCGTACCCAGGCAGAACGTGCAATGGAAACCCAGGGTAAACTGATTGCAGCAGCACTGGGTGTTCTGCGTGAAAA
AGGTTATGCAGGTTTTCGTATTGCAGATGTTCCGGGTGCAGCCGGTGTTAGCCGTGGTGCACAGAGCCATCATTTTCCGACCAAACTGGAACTGCT
GCTGGCAACCTTTGAATGGCTGTATGAGCAGATTACCGAACGTAGCCGTGCACGTCTGGCAAAACTGAAACCGGAAGATGATGTTATTCAGCAGAT
GCTGGATGATGCAGCAGAATTTTTTCTGGATGATGATTTTAGCATCAGCCTGGATCTGATTGTTGCAGCAGATCGTGATCCGGCACTGCGTGAAGG
TATTCAGCGTACCGTTGAACGTAATCGTTTTGTTGTTGAAGATATGTGGCTGGGTGTGCTGGTGAGCCGTGGTCTGAGCCGTGATGATGCCGAAGA
TATTCTGTGGCTGATTTTTAACAGCGTTCGTGGTCTGGCAGTTCGTAGCCTGTGGCAGAAAGATAAAGAACGTTTTGAACGTGTGCGTAATAGCAC
CCTGGAAATTGCACGTGAACGTTATGCAAAATTCAAACGTTGATAACTCGGTACCAAAGAAAAATAAAAAGACGCTGAAAAGCGTCTTTTTATTTT
TCGGTCCTCGTGTAAGTAGCGTAACAAACAGACAATCTGGTCTGTTTGTATTATGGAAAATTTTTCTGTATAATAGATTCAACAAACAGACAATCT
GGTCTGTTTGTATTAT

H1-HlyIIR_2 Gate

AGTAGTCACCGGCTGTGCTTGCCGGTCTGATGAGCCTGTGAAGGCGAAACTACCTCTACAAATAATTTTGTTTAAACCCCCGAGATGAAATACATC
CTGTTTGAGGTGTGCGAAATGGGTAAAAGCCGTGAACAGACCATGGAAAATATTCTGAAAGCAGCCAAAAAGAAATTCGGCGAACGTGGTTATGAA
GGCACCAGCATTCAAGAAATTACCAAAGAAGCCAAAGTTAACGTTGCAATGGCCAGCTATTACTTTAATGGCAAAGAGAACCTGTACTACGAGGTG
TTCAAAAAATACGGTCTGGCAAATGAACTGCCGAACTTTCTGGAAAAAAACCAGTTTAATCCGATTAATGCCCTGCGTGAATATCTGACCGTTTTT
ACCACCCACATTAAAGAAAATCCGGAAATTGGCACCCTGGCCTATGAAGAAATTATCAAAGAAAGCGCACGCCTGGAAAAAATCAAACCGTATTTT
ATCGGCAGCTTCGAACAGCTGAAAGAAATTCTGCAAGAGGGTGAAAAACAGGGTGTGTTTCACTTTTTTAGCATCAACCATACCATCCATTGGATT
ACCAGCATTGTTCTGTTTCCGAAATTCAAAAAATTCATCGATAGCCTGGGTCCGAATGAAACCAATGATACCAATCATGAATGGATGCCGGAAGAT
CTGGTTAGCCGTATTATTAGCGCACTGACCGATAAACCGAACATTTAAGTTATGAGTCAGGAAAAAAGGCGACAGAGTAATCTGTCGCCTTTTTTC
TTTGCTTGCTTTACCAGGAATCTGAACGATTCGTTACCAATTGCCATATTTAAAATTCTTGTTTAAAATGCTAGC

N1-LmrA_2 Gate

AGGAGTCAATTAATGTGCTTTTAATTCTGATGAGACGGTGACGTCGAAACTCCCTCTACAAATAATTTTGTTTAATACGCTATGGACTATGTTTTC
TGCTATGGACTATGTTTTCACACACGAGATGCCTCGATGAGCTATGGTGATAGCCGTGAAAAAATTCTGAGCGCAGCAACCCGTCTGTTTCAGCTG
CAGGGTTATTATGGCACCGGTCTGAATCAGATTATCAAAGAAAGCGGTGCACCGAAAGGTAGCCTGTATTATCATTTTCCGGGTGGTAAAGAACAG
CTGGCAATTGAAGCAGTGAACGAAATGAAAGAATATATCCGCCAGAAAATCGCCGATTGTATGGAAGCATGTACCGATCCGGCAGAAGGTATTCAG
GCATTTCTGAAAGAACTGAGCTGTCAGTTTAGCTGTACCGAAGATATTGAAGGTCTGCCGGTTGGTCTGCTGGCAGCAGAAACCAGCCTGAAAAGC
GAACCGCTGCGTGAAGCATGTCATGAAGCATATAAAGAATGGGCCAGCGTGTATGAAGAAAAACTGCGTCAGACCGGTTGTAGCGAAAGCCGTGCA
AAAGAAGCAAGCACCGTTGTTAATGCAATGATTGAAGGTGGTATTCTGCTGAGCCTGACCGCAAAAAATAGCACACCGCTGCTGCATATTAGCAGC
TGTATTCCGGATCTGCTGAAACGTTAATAAGGTTGAAAAATAAAAACGGCGCTAAAAAGCGCCGTTTTTTTTGACGGTGGTACGCTCATTCACTAG
GTCTGATTCGTTACCAATTGACAGCTGGTGGTCGAATCAAGATAATAGACCAGTCACTATATTT

P1-PhlF Gate

AGCGGTCAACGCATGTGCTTTGCGTTCTGATGAGACAGTGATGTCGAAACCGCCTCTACAAATAATTTTGTTTAACTATGGACTATGTTTGAAAGG
GAGAAATACTAGATGGCACGTACCCCGAGCCGTAGCAGCATTGGTAGCCTGCGTAGTCCGCATACCCATAAAGCAATTCTGACCAGCACCATTGAA
ATCCTGAAAGAATGTGGTTATAGCGGTCTGAGCATTGAAAGCGTTGCACGTCGTGCCGGTGCAAGCAAACCGACCATTTATCGTTGGTGGACCAAT
AAAGCAGCACTGATTGCCGAAGTGTATGAAAATGAAAGCGAACAGGTGCGTAAATTTCCGGATCTGGGTAGCTTTAAAGCCGATCTGGATTTTCTG
CTGCGTAATCTGTGGAAAGTTTGGCGTGAAACCATTTGTGGTGAAGCATTTCGTTGTGTTATTGCAGAAGCACAGCTGGACCCTGCAACCCTGACC
CAGCTGAAAGATCAGTTTATGGAACGTCGTCGTGAGATGCCGAAAAAACTGGTTGAAAATGCCATTAGCAATGGTGAACTGCCGAAAGATACCAAT
CGTGAACTGCTGCTGGATATGATTTTTGGTTTTTGTTGGTATCGCCTGCTGACCGAACAGCTGACCGTTGAACAGGATATTGAAGAATTTACCTTC
CTGCTGATTAATGGTGTTTGTCCGGGTACACAGCGTTAAGGAAACACAGAAAAAAGCCCGCACCTGACAGTGCGGGCTTTTTTTTTCGACCAAAGG
CGACGTACGGTGGAATCTGATTCGTTACCAATTGACATGATACGAAACGTACCGTATCGTTAAGGT

P2-PhlF	Gate	AGCGGTCAACGCATGTGCTTTGCGTTCTGATGAGACAGTGATGTCGAAACCGCCTCTACAAATAATTTTGTTTAAGGAGCTATGGACTATGTTTGA<br>AAGGCTGAAATACTAGATGGCACGTACCCCGAGCCGTAGCAGCATTGGTAGCCTGCGTAGTCCGCATACCCATAAAGCAATTCTGACCAGCACCAT<br>TGAAATCCTGAAAGAATGTGGTTATAGCGGTCTGAGCATTGAAAGCGTTGCACGTCGTGCCGGTGCAAGCAAACCGACCATTTATCGTTGGTGGAC<br>CAATAAAGCAGCACTGATTGCCGAAGTGTATGAAAATGAAAGCGAACAGGTGCGTAAATTTCCGGATCTGGGTAGCTTTAAAGCCGATCTGGATTT<br>TCTGCTGCGTAATCTGTGGAAAGTTTGGCGTGAAACCATTTGTGGTGAAGCATTTCGTTGTGTTATTGCAGAAGCACAGCTGGACCCTGCAACCCT<br>GACCCAGCTGAAAGATCAGTTTATGGAACGTCGTCGTGAGATGCCGAAAAAACTGGTTGAAAATGCCATTAGCAATGGTGAACTGCCGAAAGATAC<br>CAATCGTGAACTGCTGCTGGATATGATTTTTGGTTTTTGTTGGTATCGCCTGCTGACCGAACAGCTGACCGTTGAACAGGATATTGAAGAATTTAC<br>CTTCCTGCTGATTAATGGTGTTTGTCCGGGTACACAGCGTTAAGGAAACACAGAAAAAAGCCCGCACCTGACAGTGCGGGCTTTTTTTTTCGACCA<br>AAGGCGACGTACGGTGGAATCTGATTCGTTACCAATTGACATGATACGAAACGTACCGTATCGTTAAGGT
P3-PhlF	Gate	AGCGGTCAACGCATGTGCTTTGCGTTCTGATGAGACAGTGATGTCGAAACCGCCTCTACAAATAATTTTGTTTAACTTTACGAGGGCGATCCTATG<br>GCACGTACCCCGAGCCGTAGCAGCATTGGTAGCCTGCGTAGTCCGCATACCCATAAAGCAATTCTGACCAGCACCATTGAAATCCTGAAAGAATGT<br>GGTTATAGCGGTCTGAGCATTGAAAGCGTTGCACGTCGTGCCGGTGCAAGCAAACCGACCATTTATCGTTGGTGGACCAATAAAGCAGCACTGATT<br>GCCGAAGTGTATGAAAATGAAAGCGAACAGGTGCGTAAATTTCCGGATCTGGGTAGCTTTAAAGCCGATCTGGATTTTCTGCTGCGTAATCTGTGG<br>AAAGTTTGGCGTGAAACCATTTGTGGTGAAGCATTTCGTTGTGTTATTGCAGAAGCACAGCTGGACCCTGCAACCCTGACCCAGCTGAAAGATCAG<br>TTTATGGAACGTCGTCGTGAGATGCCGAAAAAACTGGTTGAAAATGCCATTAGCAATGGTGAACTGCCGAAAGATACCAATCGTGAACTGCTGCTG<br>GATATGATTTTTGGTTTTTGTTGGTATCGCCTGCTGACCGAACAGCTGACCGTTGAACAGGATATTGAAGAATTTACCTTCCTGCTGATTAATGGT<br>GTTTGTCCGGGTACACAGCGTTAAGGAAACACAGAAAAAAGCCCGCACCTGACAGTGCGGGCTTTTTTTTTCGACCAAAGGCGACGTACGGTGGAA<br>TCTGATTCGTTACCAATTGACATGATACGAAACGTACCGTATCGTTAAGGT
S1-SrpR	Gate	AGCGCTCAACGGGTGTGCTTCCCGTTCTGATGAGTCCGTGAGGACGAAAGCGCCTCTACAAATAATTTTGTTTAAGAGTCTATGGACTATGTTTTC<br>ACAGAGGAGGTACCAGGATGGCACGTAAAACCGCAGCAGAAGCAGAAGAAACCCGTCAGCGTATTATTGATGCAGCACTGGAAGTTTTTGTTGCAC<br>AGGGTGTTAGTGATGCAACCCTGGATCAGATTGCACGTAAAGCCGGTGTTACCCGTGGTGCAGTTTATTGGCATTTTAATGGTAAACTGGAAGTTC<br>TGCAGGCAGTTCTGGCAAGCCGTCAGCATCCGCTGGAACTGGATTTTACACCGGATCTGGGTATTGAACGTAGCTGGGAAGCAGTTGTTGTTGCAA<br>TGCTGGATGCAGTTCATAGTCCGCAGAGCAAACAGTTTAGCGAAATTCTGATTTATCAGGGTCTGGATGAAAGCGGTCTGATTCATAATCGTATGG<br>TTCAGGCAAGCGATCGTTTTCTGCAGTATATTCATCAGGTTCTGCGTCATGCAGTTACCCAGGGTGAACTGCCGATTAATCTGGATCTGCAGACCA<br>GCATTGGTGTTTTTAAAGGTCTGATTACCGGTCTGCTGTATGAAGGTCTGCGTAGCAAAGATCAGCAGGCACAGATTATCAAAGTTGCACTGGGTA<br>GCTTTTGGGCACTGCTGCGTGAACCGCCTCGTTTTCTGCTGTGTGAAGAAGCACAGATTAAACAGGTGAAATCCTTCGAATAATTCAGCCAAAAAA<br>CTTAAGACCGCCGGTCTTGTCCACTACCTTGCAGTAATGCGGTGGACAGGATCGGCGGTTTTCTTTTCTCTTCTCAATCTATGATTGGTCCAGATT<br>CGTTACCAATTGACAGCTAGCTCAGTCCTAGGTATATACATACATGCTTGTTTGTTTGTAAAC
S2-SrpR	Gate	AGCGCTCAACGGGTGTGCTTCCCGTTCTGATGAGTCCGTGAGGACGAAAGCGCCTCTACAAATAATTTTGTTTAAGAGTCTATGGACTATGTTTTC<br>ACATATGAGATACCAGGATGGCACGTAAAACCGCAGCAGAAGCAGAAGAAACCCGTCAGCGTATTATTGATGCAGCACTGGAAGTTTTTGTTGCAC<br>AGGGTGTTAGTGATGCAACCCTGGATCAGATTGCACGTAAAGCCGGTGTTACCCGTGGTGCAGTTTATTGGCATTTTAATGGTAAACTGGAAGTTC<br>TGCAGGCAGTTCTGGCAAGCCGTCAGCATCCGCTGGAACTGGATTTTACACCGGATCTGGGTATTGAACGTAGCTGGGAAGCAGTTGTTGTTGCAA<br>TGCTGGATGCAGTTCATAGTCCGCAGAGCAAACAGTTTAGCGAAATTCTGATTTATCAGGGTCTGGATGAAAGCGGTCTGATTCATAATCGTATGG<br>TTCAGGCAAGCGATCGTTTTCTGCAGTATATTCATCAGGTTCTGCGTCATGCAGTTACCCAGGGTGAACTGCCGATTAATCTGGATCTGCAGACCA<br>GCATTGGTGTTTTTAAAGGTCTGATTACCGGTCTGCTGTATGAAGGTCTGCGTAGCAAAGATCAGCAGGCACAGATTATCAAAGTTGCACTGGGTA<br>GCTTTTGGGCACTGCTGCGTGAACCGCCTCGTTTTCTGCTGTGTGAAGAAGCACAGATTAAACAGGTGAAATCCTTCGAATAATTCAGCCAAAAAA<br>CTTAAGACCGCCGGTCTTGTCCACTACCTTGCAGTAATGCGGTGGACAGGATCGGCGGTTTTCTTTTCTCTTCTCAATCTATGATTGGTCCAGATT<br>CGTTACCAATTGACAGCTAGCTCAGTCCTAGGTATATACATACATGCTTGTTTGTTTGTAAAC
S3-SrpR	Gate	AGCGCTCAACGGGTGTGCTTCCCGTTCTGATGAGTCCGTGAGGACGAAAGCGCCTCTACAAATAATTTTGTTTAAGAGTCTATGGACTATGTTTTC<br>ACAAAGGAAGTACCAGGATGGCACGTAAAACCGCAGCAGAAGCAGAAGAAACCCGTCAGCGTATTATTGATGCAGCACTGGAAGTTTTTGTTGCAC<br>AGGGTGTTAGTGATGCAACCCTGGATCAGATTGCACGTAAAGCCGGTGTTACCCGTGGTGCAGTTTATTGGCATTTTAATGGTAAACTGGAAGTTC<br>TGCAGGCAGTTCTGGCAAGCCGTCAGCATCCGCTGGAACTGGATTTTACACCGGATCTGGGTATTGAACGTAGCTGGGAAGCAGTTGTTGTTGCAA<br>TGCTGGATGCAGTTCATAGTCCGCAGAGCAAACAGTTTAGCGAAATTCTGATTTATCAGGGTCTGGATGAAAGCGGTCTGATTCATAATCGTATGG<br>TTCAGGCAAGCGATCGTTTTCTGCAGTATATTCATCAGGTTCTGCGTCATGCAGTTACCCAGGGTGAACTGCCGATTAATCTGGATCTGCAGACCA<br>GCATTGGTGTTTTTAAAGGTCTGATTACCGGTCTGCTGTATGAAGGTCTGCGTAGCAAAGATCAGCAGGCACAGATTATCAAAGTTGCACTGGGTA<br>GCTTTTGGGCACTGCTGCGTGAACCGCCTCGTTTTCTGCTGTGTGAAGAAGCACAGATTAAACAGGTGAAATCCTTCGAATAATTCAGCCAAAAAA<br>CTTAAGACCGCCGGTCTTGTCCACTACCTTGCAGTAATGCGGTGGACAGGATCGGCGGTTTTCTTTTCTCTTCTCAATCTATGATTGGTCCAGATT<br>CGTTACCAATTGACAGCTAGCTCAGTCCTAGGTATATACATACATGCTTGTTTGTTTGTAAAC
S4-SrpR	Gate	AGCGCTCAACGGGTGTGCTTCCCGTTCTGATGAGTCCGTGAGGACGAAAGCGCCTCTACAAATAATTTTGTTTAACTATGGACTATGTTTTCACAC<br>AGGAAATACCAGGATGGCACGTAAAACCGCAGCAGAAGCAGAAGAAACCCGTCAGCGTATTATTGATGCAGCACTGGAAGTTTTTGTTGCACAGGG<br>TGTTAGTGATGCAACCCTGGATCAGATTGCACGTAAAGCCGGTGTTACCCGTGGTGCAGTTTATTGGCATTTTAATGGTAAACTGGAAGTTCTGCA<br>GGCAGTTCTGGCAAGCCGTCAGCATCCGCTGGAACTGGATTTTACACCGGATCTGGGTATTGAACGTAGCTGGGAAGCAGTTGTTGTTGCAATGCT<br>GGATGCAGTTCATAGTCCGCAGAGCAAACAGTTTAGCGAAATTCTGATTTATCAGGGTCTGGATGAAAGCGGTCTGATTCATAATCGTATGGTTCA<br>GGCAAGCGATCGTTTTCTGCAGTATATTCATCAGGTTCTGCGTCATGCAGTTACCCAGGGTGAACTGCCGATTAATCTGGATCTGCAGACCAGCAT<br>TGGTGTTTTTAAAGGTCTGATTACCGGTCTGCTGTATGAAGGTCTGCGTAGCAAAGATCAGCAGGCACAGATTATCAAAGTTGCACTGGGTAGCTT<br>TTGGGCACTGCTGCGTGAACCGCCTCGTTTTCTGCTGTGTGAAGAAGCACAGATTAAACAGGTGAAATCCTTCGAATAATTCAGCCAAAAAACTTA<br>AGACCGCCGGTCTTGTCCACTACCTTGCAGTAATGCGGTGGACAGGATCGGCGGTTTTCTTTTCTCTTCTCAATCTATGATTGGTCCAGATTCGTT<br>ACCAATTGACAGCTAGCTCAGTCCTAGGTATATACATACATGCTTGTTTGTTTGTAAAC
V1-VanR	Gate	AGTGGTCGTGATCTGAAACTCGATCACCTGATGAGCTCAAGGCAGAGCGAAACCACCTCTACAAATAATTTTGTTTAAAAGACCATTATAAGGTTT<br>GAACTATGGACATGCCTCGTATTAAACCGGGTCAGCGTGTTATGATGGCACTGCGTAAAATGATTGCAAGCGGTGAAATCAAAAGTGGTGAACGTA<br>TTGCAGAAATTCCGACCGCAGCAGCACTGGGTGTTAGCCGTATGCCGGTTCGTATCGCACTGCGTTCACTGGAACAAGAAGGTCTGGTTGTTCGTC

		TGGGTGCACGTGGTTATGCAGCCCGTGGTGTTAGCAGCGATCAGATTCGTGATGCAATTGAAGTTCGTGGTGTTCTGGAAGGTTTTGCAGCACGTC<br>GTCTGGCAGAACGTGGTATGACCGCAGAAACCCATGCACGTTTTGTTGTACTGATTGCAGAAGGTGAAGCACTGTTTGCAGCCGGTCGCCTGAATG<br>GTGAAGATCTGGATCGTTATGCCGCATATAATCAGGCATTTCATGATACCCTGGTTAGCGCAGCAGGTAATGGTGCAGTTGAAAGCGCACTGGCAC<br>GTAATGGTTTTGAACCGTTTGCAGCAGCCGGTGCACTGGCCCTGGATCTGATGGACCTGTCTGCCGAATATGAACATCTGCTGGCAGCACATCGTC<br>AGCATCAGGCAGTTCTGGATGCAGTTAGCTGTGGTGATGCCGAAGGTGCAGAACGTATTATGCGTGATCATGCACTGGCAGCAATTCGTAATGCAA<br>AAGTTTTTGAAGCAGCAGCAAGCGCAGGCGCACCGCTGGGTGCAGCATGGTCAATTCGTGCAGATTGATAAAACGCATGAGAAAGCCCCCGGAAGA<br>TCACCTTCCGGGGGCTTTTTTATTGCGCTGTATAAAGTCCGCCATTGGATCCAATTGACAGCTAGCTCAGTCCTAGGTACCATTGGATCCAAT
Regulator operon for backbone JS_BB_1 [b]	Sensors	GCGGCGCGCCATCGAATGGCGCAAAACCTTTCGCGGTATGGCATGATAGCGCCCGGAAGAGAGTCAATTCAGGGTGGTGAATATGAAACCAGTAAC<br>GTTATACGATGTCGCAGAGTATGCCGGTGTCTCTTATCAGACCGTTTCCCGCGTGGTGAACCAGGCCAGCCACGTTTCTGCGAAAACGCGGGAAAA<br>AGTGGAAGCGGCGATGGCGGAGCTGAATTACATTCCCAACCGCGTGGCACAACAACTGGCGGGCAAACAGTCGTTGCTGATTGGCGTTGCCACCTC<br>CAGTCTGGCCCTGCACGCGCCGTCGCAAATTGTCGCGGCGATTAAATCTCGCGCCGATCAACTGGGTGCCAGCGTGGTGGTGTCGATGGTAGAACG<br>AAGCGGCGTCGAAGCCTGTAAAGCGGCGGTGCACAATCTTCTCGCGCAACGCGTCAGTGGGCTGATCATTAACTATCCGCTGGATGACCAGGATGC<br>CATTGCTGTGGAAGCTGCCTGCACTAATGTTCCGGCGTTATTTCTTGATGTCTCTGACCAGACACCCATCAACAGTATTATTTTCTCCCATGAGGA<br>CGGTACGCGACTGGGCGTGGAGCATCTGGTCGCATTGGGTCACCAGCAAATCGCGCTGTTAGCGGGCCCATTAAGTTCTGTCTCGGCGCGTCTGCG<br>TCTGGCTGGCTGGCATAAATATCTCACTCGCAATCAAATTCAGCCGATAGCGGAACGGGAAGGCGACTGGAGTGCCATGTCCGGTTTTCAACAAAC<br>CATGCAAATGCTGAATGAGGGCATCGTTCCCACTGCGATGCTGGTTGCCAACGATCAGATGGCGCTGGGCGCAATGCGCGCCATTACCGAGTCCGG<br>GCTGCGCGTTGGTGCGGATATCTCGGTAGTGGGATACGACGATACCGAAGATAGCTCATGTTATATCCCGCCGTTAACCACCATCAAACAGGATTT<br>TCGCCTGCTGGGGCAAACCAGCGTGGACCGCTTGCTGCAACTCTCTCAGGGCCAGGCGGTGAAGGGCAATCAGCTGTTGCCAGTCTCACTGGTGAA<br>AAGAAAAACCACCCTGGCGCCCAATACGCAAACCGCCTCTCCCCGCGCGTTGGCCGATTCATTAATGCAGCTGGCACGACAGGTTTCCCGACTGGA<br>AAGCGGGCAGTGATAATCCAGGAGGAAAAAAATGTCCAGATTAGATAAAAGTAAAGTGATTAACAGCGCATTAGAGCTGCTTAATGAGGTCGGAAT<br>CGAAGGTTTAACAACCCGTAAACTCGCCCAGAAGCTAGGTGTAGAGCAGCCTACATTGTATTGGCATGTAAAAAATAAGCGGGCTTTGCTCGACGC<br>CTTAGCCATTGAGATGTTAGATAGGCACCATACTCACTTTTGCCCTTTAGAAGGGGAAAGCTGGCAAGATTTTTTACGTAATAACGCTAAAAGTTT<br>TAGATGTGCTTTACTAAGTCATCGCGATGGAGCAAAAGTACATTTAGGTACACGGCCTACAGAAAAACAGTATGAAACTCTCGAAAATCAATTAGC<br>CTTTTTATGCCAACAAGGTTTTTCACTAGAGAATGCATTATATGCACTCAGCGCTGTGGGGCATTTTACTTTAGGTTGCGTATTGGAAGATCAAGA<br>GCATCAAGTCGCTAAAGAAGAAAGGGAAACACCTACTACTGATAGTATGCCGCCATTATTACGACAAGCTATCGAATTATTTGATCACCAAGGTGC<br>AGAGCCAGCCTTCTTATTCGGCCTTGAATTGATCATATGCGGATTAGAAAAACAACTTAAATGTGAAAGTGGGTCCTAATAATTGGTAACGAATCA<br>GACAATTGACGGCTCGAGGGAGTAGCATAGGGTTTGCAGAATCCCTGCTTCGTCCATTTGACAGGCACATTATGCATCGATGATAAGCTGTCAAAC<br>ATGAGCAGATCCTCTACGCCGGACGCATCGTGGCCGGCATCACCGGCGCCACAGGTGCGGTTGCTGGCGCCTATATCGCCGACATCACCGATGGGG<br>AAGATCGGGCTCGCCACTTCGGGCTCATGAGCAAATATTTTATCTG
Regulator operon for backbone JS_BB_2 [c]	Sensors	GCGGCGCGCCATCGAATGGCGCAAAACCTTTCGCGGTATGGCATGATAGCGCCCGGAAGAGAGTCAATTCAGGGTGGTGAATATGAAACCAGTAAC<br>GTTATACGATGTCGCAGAGTATGCCGGTGTCTCTTATCAGACCGTTTCCCGCGTGGTGAACCAGGCCAGCCACGTTTCTGCGAAAACGCGGGAAAA<br>AGTGGAAGCGGCGATGGCGGAGCTGAATTACATTCCCAACCGCGTGGCACAACAACTGGCGGGCAAACAGTCGTTGCTGATTGGCGTTGCCACCTC<br>CAGTCTGGCCCTGCACGCGCCGTCGCAAATTGTCGCGGCGATTAAATCTCGCGCCGATCAACTGGGTGCCAGCGTGGTGGTGTCGATGGTAGAACG<br>AAGCGGCGTCGAAGCCTGTAAAGCGGCGGTGCACAATCTTCTCGCGCAACGCGTCAGTGGGCTGATCATTAACTATCCGCTGGATGACCAGGATGC<br>CATTGCTGTGGAAGCTGCCTGCACTAATGTTCCGGCGTTATTTCTTGATGTCTCTGACCAGACACCCATCAACAGTATTATTTTCTCCCATGAGGA<br>CGGTACGCGACTGGGCGTGGAGCATCTGGTCGCATTGGGTCACCAGCAAATCGCGCTGTTAGCGGGCCCATTAAGTTCTGTCTCGGCGCGTCTGCG<br>TCTGGCTGGCTGGCATAAATATCTCACTCGCAATCAAATTCAGCCGATAGCGGAACGGGAAGGCGACTGGAGTGCCATGTCCGGTTTTCAACAAAC<br>CATGCAAATGCTGAATGAGGGCATCGTTCCCACTGCGATGCTGGTTGCCAACGATCAGATGGCGCTGGGCGCAATGCGCGCCATTACCGAGTCCGG<br>GCTGCGCGTTGGTGCGGATATCTCGGTAGTGGGATACGACGATACCGAAGATAGCTCATGTTATATCCCGCCGTTAACCACCATCAAACAGGATTT<br>TCGCCTGCTGGGGCAAACCAGCGTGGACCGCTTGCTGCAACTCTCTCAGGGCCAGGCGGTGAAGGGCAATCAGCTGTTGCCAGTCTCACTGGTGAA<br>AAGAAAAACCACCCTGGCGCCCAATACGCAAACCGCCTCTCCCCGCGCGTTGGCCGATTCATTAATGCAGCTGGCACGACAGGTTTCCCGACTGGA<br>AAGCGGGCAGTGATAATCCAGGAGGAAAAAAATGTCCAGATTAGATAAAAGTAAAGTGATTAACAGCGCATTAGAGCTGCTTAATGAGGTCGGAAT<br>CGAAGGTTTAACAACCCGTAAACTCGCCCAGAAGCTAGGTGTAGAGCAGCCTACATTGTATTGGCATGTAAAAAATAAGCGGGCTTTGCTCGACGC<br>CTTAGCCATTGAGATGTTAGATAGGCACCATACTCACTTTTGCCCTTTAGAAGGGGAAAGCTGGCAAGATTTTTTACGTAATAACGCTAAAAGTTT<br>TAGATGTGCTTTACTAAGTCATCGCGATGGAGCAAAAGTACATTTAGGTACACGGCCTACAGAAAAACAGTATGAAACTCTCGAAAATCAATTAGC<br>CTTTTTATGCCAACAAGGTTTTTCACTAGAGAATGCATTATATGCACTCAGCGCTGTGGGGCATTTTACTTTAGGTTGCGTATTGGAAGATCAAGA<br>GCATCAAGTCGCTAAAGAAGAAAGGGAAACACCTACTACTGATAGTATGCCGCCATTATTACGACAAGCTATCGAATTATTTGATCACCAAGGTGC<br>AGAGCCAGCCTTCTTATTCGGCCTTGAATTGATCATATGCGGATTAGAAAAACAACTTAAATGTGAAAGTGGGTCCTAATAATTGGTAACGAATCA<br>GACAATTGACGGCTCGAGGGAGTAGCATAGGGTTTGCAGAATCCCTGCTTCGTCCATTTGACAGGCACATTATGCATCGATGATAAGCTGTCAAAC<br>ATGAGCAGATCCTCTACGCCGGACGCATCGTGGCCGGCATCACCGGCGCCACAGGTGCGGTTGCTGGCGCCTATATCGCCGACATCACCGATGGGG<br>AAGATCGGGCTCGCCACTTCGGGCTCATGAGCAAATATTTTATCTGAGGTTTGACAGCTAGCTCAGTCCTAGGTATTGTGCTAGCTCTAGAGATTA<br>AAGAGGAGAAATACTAGATGAAAAACATAAATGCCGACGACACATACAGAATAATTAATAAAATTAAAGCTTGTAGAAGCAATAATGATATTAATC<br>AATGCTTATCTGATATGACTAAAATGGTACATTGTGAATATTATTTACTCGCAATCATTTATCCTCATTATATGGTTAAATCCGATATTTCAATCC<br>TAGATAATTACCCTAAAAAATGGAGGCAATATTATGATGACGCTAATTTAATAAAATATGATCCTATAGTAGATTATTCTAACTCCAATCATTCAC<br>CAATTAATTGGAATATATTTGAAAACAATGCTGTAAATAAAAAATCTCCAAATGTAATTAAAGAAGCGAAAACATCAGGTCTTATCACTGGGTTTA<br>GTTTCCCTATTCATACGGCTAACAATGGCTTCGGAATGCTTAGTTTTGCACATTCAGAAAAAGACAACTATATAGATAGTTTATTTTTACATGCGT<br>GTATGAACATACCATTAATTGTTCCTTCTCTAGTTGATAATTATCGAAAAATAAATATAGCAAATAATAAATCAAACAACGATTTAACCAAAAGAG<br>AAAAAGAATGTTTAGCGTGGGCATGCGAAGGAAAAAGCTCTTGGGATATTTCAAAAATATTAGGTTGCAGTGAGCGTACTGTCACTTTCCATTTAA<br>CCAATGCGCAAATGAAACTCAATACAACAAACCGCTGCCAAAGTATTTCTAAAGCAATTTTAACAGGAGCAATTGATTGCCCATACTTTAAAAATT<br>AACCCAAACAAGTACCCTCAGGACGAGATAATGATTGAGAATACCTATAGCGAAAAGTTCGAGTCCGCGTTCGAACAGATCAAAGCGGCGGCCAAC<br>GTGGATGCCGCCATCCGTATTCTCCAGGCGGAATATAACCTCGATTTCGTCACCTACCATCTCGCCCAGACAATCGCGAGCAAGATCGATTCGCCC<br>TTCGTGCGCACCACCTATCCGGATGCCTGGGTTTCCCGTTACCTCCTCAACTGCTATGTGAAGGTCGATCCGATCATCAAGCAGGGCTTCGAACGC<br>CAGCTGCCCTTCGACTGGAGCGAGGTCGAACCGACGCCGGAGGCCTATGCCATGCTGGTCGACGCCCAGAAACACGGCATCGATGACAATGGCTAC<br>TCCATCCCCGTCGCCGACAAGGCGCAGCGCCGCGCCCTGCTGTCGCTGAATGCCCATATACCGGCCGACGAATGGACCGAGCTCGTGCGCCGCTGC<br>CGCAATGAGTGGATCGAGATCGCCCATCTGATCCACCGCAAGGCCGTATATGAGCTGCATGGCGAAAACGATCCGGTGCCGGCATTGTCGCCGCGC<br>GAGATCGAGTGTCTGCACTGGACCGCCCTCGGCAAGGATTACAAGGATATTTCGGTCATCCTGGGCATATCAGAGCATACCACACGCGATTACCTG<br>AAAACCGCCCGCTTCAGGCTCGGCTGCACCACGATCTCGGCCGCCGCGTCGCGGGCTGTTCAATTGCGCATCATCAATCCCTATAGGATCCGCATG<br>ACGCGACGTAATTGGTAATAGCCAGGCATCAAATAAAACGAAAGGCTCAGTCGAAAGACTGGGCCTTTCGTTTTATCTGTTGTTTGTCGGTGAACG<br>CTCTCTACTAGAGTCACACTGGCTCACCTTCGGGTGGGCCTTTCTGCGTTTATA

Regulator operon for backbone JS_BB_4 [d]	Sensors	GCGGCGCGCCATCGAATGGTGCAAAACCTTTCGCGGTATGGCATGATAGCGCCCGGAAGAGAGTCAATTCAGGGTGGTGAATATGAAAAACATAAA<br>TGCCGACGACACATACAGAATAATTAATAAAATTAAAGCTTGTAGAAGCAATAATGATATTAATCAATGCTTATCTGATATGACTAAAATGGTACA<br>TTGTGAATATTATTTACTCGCAATCATTTATCCTCATTATATGGTTAAATCCGATATTTCAATCCTAGATAATTACCCTAAAAAATGGAGGCAATA<br>TTATGATGACGCTAATTTAATAAAATATGATCCTATAGTAGATTATTCTAACTCCAATCATTCACCAATTAATTGGAATATATTTGAAAACAATGC<br>TGTAAATAAAAAATCTCCAAATGTAATTAAAGAAGCGAAAACATCAGGTCTTATCACTGGGTTTAGTTTCCCTATTCATACGGCTAACAATGGCTT<br>CGGAATGCTTAGTTTTGCACATTCAGAAAAAGACAACTATATAGATAGTTTATTTTTACATGCGTGTATGAACATACCATTAATTGTTCCTTCTCT<br>AGTTGATAATTATCGAAAAATAAATATAGCAAATAATAAATCAAACAACGATTTAACCAAAAGAGAAAAAGAATGTTTAGCGTGGGCATGCGAAGG<br>AAAAAGCTCTTGGGATATTTCAAAAATATTAGGTTGCAGTGAGCGTACTGTCACTTTCCATTTAACCAATGCGCAAATGAAACTCAATACAACAAA<br>CCGCTGCCAAAGTATTTCTAAAGCAATTTTAACAGGAGCAATTGATTGCCCATACTTTAAAAATTAACAATTCGAGGAGACGTACATGGCTGAAGC<br>GCAAAATGATCCCCTGCTGCCGGGATACTCGTTTAATGCCCATCTGGTGGCGGGTTTAACGCCGATTGAGGCCAACGGTTATCTCGATTTTTTTAT<br>CGACCGACCGCTGGGAATGAAAGGTTATATTCTCAATCTCACCATTCGCGGTCAGGGGGTGGTGAAAAATCAGGGACGAGAATTTGTTTGCCGACC<br>GGGTGATATTTTGCTGTTCCCGCCAGGAGAGATTCATCACTACGGTCGTCATCCGGAGGCTCGCGAATGGTATCACCAGTGGGTTTACTTTCGTCC<br>GCGCGCCTACTGGCATGAATGGCTTAACTGGCCGTCAATATTTGCCAATACGGGGTTCTTTCGCCCGGATGAAGCGCACCAGCCGCATTTCAGCGA<br>CCTGTTTGGGCAAATCATTAACGCCGGGCAAGGGGAAGGGCGCTATTCGGAGCTGCTGGCGATAAATCTGCTTGAGCAATTGTTACTGCGGCGCAT<br>GGAAGCGATTAACGAGTCGCTCCATCCACCGATGGATAATCGGGTACGCGAGGCTTGTCAGTACATCAGCGATCACCTGGCAGACAGCAATTTTGA<br>TATCGCCAGCGTCGCACAGCATGTTTGCTTGTCGCCGTCGCGTCTGTCACATCTTTTCCGCCAGCAGTTAGGGATTAGCGTCTTAAGCTGGCGCGA<br>GGACCAACGTATCAGCCAGGCGAAGCTGCTTTTGAGCACCACCCGGATGCCTATCGCCACCGTCGGTCGCAATGTTGGTTTTGACGATCAACTCTA<br>TTTCTCGCGGGTATTTAAAAAATGCACCGGGGCCAGCCCGAGCGAGTTCCGTGCCGGTTAAGGAAGAGAGTCAATTCAGGGTGGTGAATATGAAAC<br>CAGTAACGTTATACGATGTCGCAGAGTATGCCGGTGTCTCTTATCAGACCGTTTCCCGCGTGGTGAACCAGGCCAGCCACGTTTCTGCGAAAACGC<br>GGGAAAAAGTGGAAGCGGCGATGGCGGAGCTGAATTACATTCCCAACCGCGTGGCACAACAACTGGCGGGCAAACAGTCGTTGCTGATTGGCGTTG<br>CCACCTCCAGTCTGGCCCTGCACGCGCCGTCGCAAATTGTCGCGGCGATTAAATCTCGCGCCGATCAACTGGGTGCCAGCGTGGTGGTGTCGATGG<br>TAGAACGAAGCGGCGTCGAAGCCTGTAAAGCGGCGGTGCACAATCTTCTCGCGCAACGCGTCAGTGGGCTGATCATTAACTATCCGCTGGATGACC<br>AGGATGCCATTGCTGTGGAAGCTGCCTGCACTAATGTTCCGGCGTTATTTCTTGATGTCTCTGACCAGACACCCATCAACAGTATTATTTTCTCCC<br>ATGAGGACGGTACGCGACTGGGCGTGGAGCATCTGGTCGCATTGGGTCACCAGCAAATCGCGCTGTTAGCGGGCCCATTAAGTTCTGTCTCGGCGC<br>GTCTGCGTCTGGCTGGCTGGCATAAATATCTCACTCGCAATCAAATTCAGCCGATAGCGGAACGGGAAGGCGACTGGAGTGCCATGTCCGGTTTTC<br>AACAAACCATGCAAATGCTGAATGAGGGCATCGTTCCCACTGCGATGCTGGTTGCCAACGATCAGATGGCGCTGGGCGCAATGCGCGCCATTACCG<br>AGTCCGGGCTGCGCGTTGGTGCGGATATCTCGGTAGTGGGATACGACGATACCGAAGATAGCTCATGTTATATCCCGCCGTTAACCACCATCAAAC<br>AGGATTTTCGCCTGCTGGGGCAAACCAGCGTGGACCGCTTGCTGCAACTCTCTCAGGGCCAGGCGGTGAAGGGCAATCAGCTGTTGCCAGTCTCAC<br>TGGTTAAAAGAAAAACCACCCTGGCGCCCAATACGCAAACCGCCTCTCCCCGCGCGTTGGCCGATTCATTAATGCAGCTGGCACGACAGGTTTCCC<br>GACTGGAAAGCGGGCAGTGATAAATTCCAACCTACCCCCGCGAGTAAGCTCAGTTCCAATGTCCAGATTAGATAAAAGTAAAGTGATTAACAGCGC<br>ATTAGAGCTGCTTAATGAGGTCGGAATCGAAGGTTTAACAACCCGTAAACTCGCCCAGAAGCTAGGTGTAGAGCAGCCTACATTGTATTGGCATGT<br>AAAAAATAAGCGGGCTTTGCTCGACGCCTTAGCCATTGAGATGTTAGATAGGCACCATACTCACTTTTGCCCTTTAGAAGGGGAAAGCTGGCAAGA<br>TTTTTTACGTAATAACGCTAAAAGTTTTAGATGTGCTTTACTAAGTCATCGCGATGGAGCAAAAGTACATTTAGGTACACGGCCTACAGAAAAACA<br>GTATGAAACTCTCGAAAATCAATTAGCCTTTTTATGCCAACAAGGTTTTTCACTAGAGAATGCATTATATGCACTCAGCGCTGTGGGGCATTTTAC<br>TTTAGGTTGCGTATTGGAAGATCAAGAGCATCAAGTCGCTAAAGAAGAAAGGGAAACACCTACTACTGATAGTATGCCGCCATTATTACGACAAGC<br>TATCGAATTATTTGATCACCAAGGTGCAGAGCCAGCCTTCTTATTCGGCCTTGAATTGATCATATGCGGATTAGAAAAACAACTTAAATGTGAAAG<br>TGGGTCCTAATAATTGGTAACGAATCAGACAATTGACGGCTCGAGGGAGTAGCATAGGGTTTGCAGAATCCCTGCTTCGTCCATTTGACAGGCACA<br>TTATGCATCGATGATAAGCTGTCAAACATGAGCAGATCCTCTACGCCGGACGCATCGTGGCCGGCATCACCGGCGCCACAGGTGCGGTTGCTGGCG<br>CCTATATCGCCGACATCACCGATGGGGAAGATCGGGCTCGCCACTTCGGGCTCATGAGCAAATATTTTATCTG

a. DNA sequence colors: promoters (orange), ribozyme insulator (blue), RBS (green), open reading frames (red), terminators (black).
b. LacI and TetR expression cassette driven by constitutive P_{LacI} promoter. The plasmid map is shown in Appendix Fig S17A.
c. LacI and TetR expression cassette driven by constitute P_{LacI} promoter and LuxR and CinR expression cassette driven by constitute promoter J23104. The plasmid map is shown in Appendix Fig S17B.
d. LuxR, AraC*, LacI, and TetR expression cassette driven by constitutive P_{LacIQ} promoter. The plasmid map is shown in Appendix Fig S18.

Appendix Table S4: Genetic part sequence used in this work.

Part name	Type	DNA sequence
P_{AmeR2}	Promoter	TCGTCACTAGAGGGCGATAGTGACAAACTTGACAACTCATCACTTCCTACGTAGGCTGCTAGC
P_{AmtR2}	Promoter	CTTGTCCAACCAAATGATTCGTTACCCTTTGACAGTTTCTATCGATCTATAGATAATGCTAGC
P_{BetI2}	Promoter	AGCGCGGGTGAGAGGGATTCGTTACCAATAGACAATTGATTGGACGTTCAATATAATGCTAGC
P_{BM3R1}	Promoter	AATCCGCGTGATAGGTCTGATTCGTTACCAATTGACGGAATGAACGTTCATTCCGATAATGCTAGC
P_{CymR}	Promoter	TCGTGTAAGTAGCGTAACAAACAGACAATCTGGTCTGTTTGTATTATGGAAAATTTTTCTGTATAATAGATTCAACAAACAGACAATC<br>TGGTCTGTTTGTATTAT
$P_{HlyIIR2}$	Promoter	ACCAGGAATCTGAACGATTCGTTACCAATTGCCATATTTAAAATTCTTGTTTAAAATGCTAGC
P_{LmrA2}	Promoter	CGCTCATTCACTAGGTCTGATTCGTTACCAATTGACAGCTGGTGGTCGAATCAAGATAATAGACCAGTCACTATATTT
P_{PhlF}	Promoter	CGACGTACGGTGGAATCTGATTCGTTACCAATTGACATGATACGAAACGTACCGTATCGTTAAGGT
P_{SrpR}	Promoter	TCTATGATTGGTCCAGATTCGTTACCAATTGACAGCTAGCTCAGTCCTAGGTATATACATACATGCTTGTTTGTTTGTAAAC
P_{VanR}	Promoter	TGTATAAAGTCCGCCATTGGATCCAATTGACAGCTAGCTCAGTCCTAGGTACCATTGGATCCAAT
P_{Tac}	Promoter	AACGATCGTTGGCTGTGTTGACAATTAATCATCGGCTCGTATAATGTGTGGAATTGTGAGCGCTCACAATT
P_{Tet}	Promoter	TACTCCACCGTTGGCTTTTTTCCCTATCAGTGATAGAGATTGACATCCCTATCAGTGATAGAGATAATGAGCAC
P_{Lux}^{*}	Promoter	ATAGCTTCTTACCGGACCTGTAGGATCGTACAGGTTTACGCAAGAAAATGGTTTGTTACTTTCGAATAAA
P_{Lux2}	Promoter	ATAGCTTCTTACCGGACCTGTAGGATCGTACAGGTTTACGCAAGAAAATGGTTTGTTATTTTCGAATAAA
P_{Cin}	Promoter	CCCTTTGTGCGTCCAAACGGACGCACGGCGCTCTAAAGCGGGTCGCGATCTTTCAGATTCGCTCCTCGCGCTTTCAGTCTTTGTTTTG<br>GCGCATGTCGTTATCGCAAAACCGCTGCACACTTTTGCGCGACATGCTCTGATCCCCCTCATCTGGGGGGGCCTATCTGAGGGAATTT<br>CCGATCCGGCTCGCCTGAACCATTCTGCTTTCCACGAACTTGAAAACGCT
P_{Bad}	Promoter	ACTTTTCATACTCCCGCCATTCAGAGAAGAAACCAATTGTCCATATTGCATCAGACATTGCCGTCACTGCGTCTTTTACTGGCTCTTC<br>TCGCTAACCAAACCGGTAACCCCGCTTATTAAAAGCATTCTGTAACAAAGCGGGACCAAAGCCATGACAAAAACGCGTAACAAAAGTG<br>TCTATAATCACGGCAGAAAAGTCCACATTGATTATTTGCACGGCGTCACACTTTGCTATGCCATAGCATTTTTATCCATAAGATTAGC<br>GGATCCTACCTGACGCTTTTTATCGCAACTCTCTACTGTTTCTCCATACCCGTTTTTTTGGGCTAGCCTGATGGTCCTAGCGGGTCTA<br>ACTGA
P_{LacI}	Promoter	GCGGCGCGCCATCGAATGGCGCAAAACCTTTCGCGGTATGGCATGATAGCGCCCGGAAGAGAGTCAATTCAGGGTGGTGAAT
J23104	Promoter	TTGACAGCTAGCTCAGTCCTAGGTATTGTGCTAGC
P_{LacIQ}	Promoter	GCGGCGCGCCATCGAATGGTGCAAAACCTTTCGCGGTATGGCATGATAGCGCCCGGAAGAGAGTCAATTCAGGGTGGTGAAT
J23101	Promoter	GATAAGTCCCTAACTTTTACAGCTAGCTCAGTCCTAGGTATTATGCTAGC
RiboJ54	Ribozyme	AGGGGTCAGTTGATGTGCTTTCAACTCTGATGAGTCAGTGATGACGAAACCCCCTCTACAAATAATTTTGTTTAA
BydvJ	Ribozyme	AGGGTGTCTCAAGGTGCGTACCTTGACTGATGAGTCCGAAAGGACGAAACACCCCTCTACAAATAATTTTGTTTAA
RiboJ57	Ribozyme	AGAAGTCAATTAATGTGCTTTTAATTCTGATGAGTCGGTGACGACGAAACTTCCTCTACAAATAATTTTGTTTAA
SarJ	Ribozyme	AGACTGTCGCCGGATGTGTATCCGACCTGACGATGGCCCAAAAGGGCCGAAACAGTCCTCTACAAATAATTTTGTTTAA

LtsvJ	Ribozyme	AGTACGTCTGAGCGTGATACCCGCTCACTGAAGATGGCCCGGTAGGGCCGAAACGTACCTCTACAAATAATTTTGTTTAA
RiboJ51	Ribozyme	AGTAGTCACCGGCTGTGCTTGCCGGTCTGATGAGCCTGTGAAGGCGAAACTACCTCTACAAATAATTTTGTTTAA
RiboJ64	Ribozyme	AGGAGTCAATTAATGTGCTTTTAATTCTGATGAGACGGTGACGTCGAAACTCCCTCTACAAATAATTTTGTTTAA
RiboJ53	Ribozyme	AGCGGTCAACGCATGTGCTTTGCGTTCTGATGAGACAGTGATGTCGAAACCGCCTCTACAAATAATTTTGTTTAA
RiboJ10	Ribozyme	AGCGCTCAACGGGTGTGCTTCCCGTTCTGATGAGTCCGTGAGGACGAAAGCGCCTCTACAAATAATTTTGTTTAA
AraJ	Ribozyme	AGTGGTCGTGATCTGAAACTCGATCACCTGATGAGCTCAAGGCAGAGCGAAACCACCTCTACAAATAATTTTGTTTAA
RiboJ	Ribozyme	AGCTGTCACCGGATGTGCTTTCCGGTCTGATGAGTCCGTGAGGACGAAACAGCCTCTACAAATAATTTTGTTTAA
F1	RBS	CTATGGACTATGTTTTCACATACGAGGGGGATTAG
F2	RBS	TACGCTATGGACTATGTTTCAAACAGGAGCTAATAG
A1	RBS	AATGTTCCCTAATAATCAGCAAAGAGGTTACTAG
E1	RBS	CCCCCCGAGGAGTAGCAC
B1	RBS	CTATGGACTATGTTTTAACTACTAG
B2	RBS	CTATGGACTATGTTTTTCAAAGACGAAAAACTACTAG
B3	RBS	CCAAACGAGGCCGGGAGG
C1	RBS	GACTTAAACGATTGGCCGACCGC
H1	RBS	ACCCCCGAG
N1	RBS	TACGCTATGGACTATGTTTTCTGCTATGGACTATGTTTTCACACACGAGATGCCTCG
P1	RBS	CTATGGACTATGTTTGAAAGGGAGAAATACTAG
P2	RBS	GGAGCTATGGACTATGTTTGAAAGGCTGAAATACTAG
P3	RBS	CTTTACGAGGGCGATCCT
S1	RBS	GAGTCTATGGACTATGTTTTCACAGAGGAGGTACCAGG
S2	RBS	GAGTCTATGGACTATGTTTTCACATATGAGATACCAGG
S3	RBS	GAGTCTATGGACTATGTTTTCACAAAGGAAGTACCAGG
S4	RBS	CTATGGACTATGTTTTCACACAGGAAATACCAGG
V1	RBS	AAGACCATTATAAGGTTTGAACT
B0064	RBS	TACTAGAGAAAGAGGGGAAATACTAG
ameR	Gene	ATGAACAAAACCATTGATCAGGTGCGTAAAGGTGATCGTAAAAGCGATCTGCCGGTTCGTCGTCGTCCGCGTCGTAGTGCCGAAGAAA<br>CCCGTCGTGATATTCTGGCAAAAGCCGAAGAACTGTTTCGTGAACGTGGTTTTAATGCAGTTGCCATTGCAGATATTGCAAGCGCACT<br>GAATATGAGTCCGGCAAATGTGTTTAAACATTTTAGCAGCAAAAACGCACTGGTTGATGCAATTGGTTTTGGTCAGATTGGTGTTTTT<br>GAACGTCAGATTTGTCCGCTGGATAAAAGCCATGCACCGCTGGATCGTCTGCGTCATCTGGCACGTAATCTGATGGAACAGCATCATC<br>AGGATCATTTCAAACACATACGGGTTTTTATTCAGATCCTGATGACCGCCAAACAGGATATGAAATGTGGCGATTATTACAAAAGCGT<br>GATTGCAAAACTGCTGGCCGAAATTATTCGTGATGGTGTTGAAGCAGGTCTGTATATTGCAACCGATATTCCGGTTCTGGCAGAAACC

		GTTCTGCATGCACTGACCAGCGTTATTCATCCGGTTCTGATTGCACAAGAAGATATTGGTAATCTGGCAACCCGTTGTGATCAGCTGG<br>TTGATCTGATTGATGCAGGTCTGCGTAATCCGCTGGCAAAATAA
amtR	Gene	ATGGCAGGCGCAGTTGGTCGTCCGCGTCGTAGTGCACCGCGTCGTGCAGGTAAAAATCCGCGTGAAGAAATTCTGGATGCAAGCGCAG<br>AACTGTTTACCCGTCAGGGTTTTGCAACCACCAGTACCCATCAGATTGCAGATGCAGTTGGTATTCGTCAGGCAAGCCTGTATTATCA<br>TTTTCCGAGCAAAACCGAAATCTTTCTGACCCTGCTGAAAAGCACCGTTGAACCGAGCACCGTTCTGGCAGAAGATCTGAGCACCCTG<br>GATGCAGGTCCGGAAATGCGTCTGTGGGCAATTGTTGCAAGCGAAGTTCGTCTGCTGCTGAGCACCAAATGGAATGTTGGTCGTCTGT<br>ATCAGCTGCCGATTGTTGGTAGCGAAGAATTTGCAGAATATCATAGCCAGCGTGAAGCACTGACCAATGTTTTTCGTGATCTGGCAAC<br>CGAAATTGTTGGTGATGATCCGCGTGCAGAACTGCCGTTTCATATTACCATGAGCGTTATTGAAATGCGTCGCAATGATGGTAAAATT<br>CCGAGTCCGCTGAGCGCAGATAGCCTGCCGGAAACCGCAATTATGCTGGCAGATGCAAGCCTGGCAGTTCTGGGTGCACCGCTGCCTG<br>CAGATCGTGTTGAAAAAACCCTGGAACTGATTAAACAGGCAGATGCAAAATAA
betI	Gene	ATGCCGAAACTGGGTATGCAGAGCATTCGTCGTCGTCAGCTGATTGATGCAACCCTGGAAGCAATTAATGAAGTTGGTATGCATGATG<br>CAACCATTGCACAGATTGCACGTCGTGCCGGTGTTAGCACCGGTATTATTAGCCATTATTTCCGCGATAAAAACGGTCTGCTGGAAGC<br>AACCATGCGTGATATTACCAGCCAGCTGCGTGATGCAGTTCTGAATCGTCTGCATGCACTGCCGCAGGGTAGCGCAGAACAGCGTCTG<br>CAGGCAATTGTTGGTGGTAATTTTGATGAAACCCAGGTTAGCAGCGCAGCAATGAAAGCATGGCTGGCATTTTGGGCAAGCAGCATGC<br>ATCAGCCGATGCTGTATCGTCTGCAGCAGGTTAGCAGTCGTCGTCTGCTGAGCAATCTGGTTAGCGAATTTCGTCGTGAACTGCCTCG<br>TGAACAGGCACAAGAGGCAGGTTATGGTCTGGCAGCACTGATTGATGGTCTGTGGCTGCGTGCAGCACTGAGCGGTAAACCGCTGGAT<br>AAAACCCGTGCAAATAGCCTGACCCGTCATTTTATCACCCAGCATCTGCCGACCGATTAA
bm3R1	Gene	ATGGAAAGCACCCCGACCAAACAGAAAGCAATTTTTAGCGCAAGCCTGCTGCTGTTTGCAGAACGTGGTTTTGATGCAACCACCATGC<br>CGATGATTGCAGAAAATGCAAAAGTTGGTGCAGGCACCATTTATCGCTATTTCAAAAACAAAGAAAGCCTGGTGAACGAACTGTTTCA<br>GCAGCATGTTAATGAATTTCTGCAGTGTATTGAAAGCGGTCTGGCAAATGAACGTGATGGTTATCGTGATGGCTTTCATCACATTTTT<br>GAAGGTATGGTGACCTTTACCAAAAATCATCCGCGTGCACTGGGTTTTATCAAAACCCATAGCCAGGGCACCTTTCTGACCGAAGAAA<br>GCCGTCTGGCATATCAGAAACTGGTTGAATTTGTGTGCACCTTTTTTCGTGAAGGTCAGAAACAGGGTGTGATTCGTAATCTGCCGGA<br>AAATGCACTGATTGCAATTCTGTTTGGCAGCTTTATGGAAGTGTATGAAATGATCGAGAACGATTATCTGAGCCTGACCGATGAACTG<br>CTGACCGGTGTTGAAGAAAGCCTGTGGGCAGCACTGAGCCGTCAGAGCTAA
cymR	Gene	ATGAGCCCGAAACGTCGTACCCAGGCAGAACGTGCAATGGAAACCCAGGGTAAACTGATTGCAGCAGCACTGGGTGTTCTGCGTGAAA<br>AAGGTTATGCAGGTTTTCGTATTGCAGATGTTCCGGGTGCAGCCGGTGTTAGCCGTGGTGCACAGAGCCATCATTTTCCGACCAAACT<br>GGAACTGCTGCTGGCAACCTTTGAATGGCTGTATGAGCAGATTACCGAACGTAGCCGTGCACGTCTGGCAAAACTGAAACCGGAAGAT<br>GATGTTATTCAGCAGATGCTGGATGATGCAGCAGAATTTTTTCTGGATGATGATTTTAGCATCAGCCTGGATCTGATTGTTGCAGCAG<br>ATCGTGATCCGGCACTGCGTGAAGGTATTCAGCGTACCGTTGAACGTAATCGTTTTGTTGTTGAAGATATGTGGCTGGGTGTGCTGGT<br>GAGCCGTGGTCTGAGCCGTGATGATGCCGAAGATATTCTGTGGCTGATTTTTAACAGCGTTCGTGGTCTGGCAGTTCGTAGCCTGTGG<br>CAGAAAGATAAAGAACGTTTTGAACGTGTGCGTAATAGCACCCTGGAAATTGCACGTGAACGTTATGCAAAATTCAAACGTTGATAA
hlyIIR	Gene	ATGAAATACATCCTGTTTGAGGTGTGCGAAATGGGTAAAAGCCGTGAACAGACCATGGAAAATATTCTGAAAGCAGCCAAAAAGAAAT<br>TCGGCGAACGTGGTTATGAAGGCACCAGCATTCAAGAAATTACCAAAGAAGCCAAAGTTAACGTTGCAATGGCCAGCTATTACTTTAA<br>TGGCAAAGAGAACCTGTACTACGAGGTGTTCAAAAAATACGGTCTGGCAAATGAACTGCCGAACTTTCTGGAAAAAAACCAGTTTAAT<br>CCGATTAATGCCCTGCGTGAATATCTGACCGTTTTTACCACCCACATTAAAGAAAATCCGGAAATTGGCACCCTGGCCTATGAAGAAA<br>TTATCAAAGAAAGCGCACGCCTGGAAAAAATCAAACCGTATTTTATCGGCAGCTTCGAACAGCTGAAAGAAATTCTGCAAGAGGGTGA<br>AAAACAGGGTGTGTTTCACTTTTTTAGCATCAACCATACCATCCATTGGATTACCAGCATTGTTCTGTTTCCGAAATTCAAAAAATTC<br>ATCGATAGCCTGGGTCCGAATGAAACCAATGATACCAATCATGAATGGATGCCGGAAGATCTGGTTAGCCGTATTATTAGCGCACTGA<br>CCGATAAACCGAACATTTAA
lmrA	Gene	ATGAGCTATGGTGATAGCCGTGAAAAAATTCTGAGCGCAGCAACCCGTCTGTTTCAGCTGCAGGGTTATTATGGCACCGGTCTGAATC<br>AGATTATCAAAGAAAGCGGTGCACCGAAAGGTAGCCTGTATTATCATTTTCCGGGTGGTAAAGAACAGCTGGCAATTGAAGCAGTGAA<br>CGAAATGAAAGAATATATCCGCCAGAAAATCGCCGATTGTATGGAAGCATGTACCGATCCGGCAGAAGGTATTCAGGCATTTCTGAAA<br>GAACTGAGCTGTCAGTTTAGCTGTACCGAAGATATTGAAGGTCTGCCGGTTGGTCTGCTGGCAGCAGAAACCAGCCTGAAAAGCGAAC<br>CGCTGCGTGAAGCATGTCATGAAGCATATAAAGAATGGGCCAGCGTGTATGAAGAAAAACTGCGTCAGACCGGTTGTAGCGAAAGCCG<br>TGCAAAAGAAGCAAGCACCGTTGTTAATGCAATGATTGAAGGTGGTATTCTGCTGAGCCTGACCGCAAAAAATAGCACACCGCTGCTG<br>CATATTAGCAGCTGTATTCCGGATCTGCTGAAACGTTAA
phlF	Gene	ATGGCACGTACCCCGAGCCGTAGCAGCATTGGTAGCCTGCGTAGTCCGCATACCCATAAAGCAATTCTGACCAGCACCATTGAAATCC<br>TGAAAGAATGTGGTTATAGCGGTCTGAGCATTGAAAGCGTTGCACGTCGTGCCGGTGCAAGCAAACCGACCATTTATCGTTGGTGGAC<br>CAATAAAGCAGCACTGATTGCCGAAGTGTATGAAAATGAAAGCGAACAGGTGCGTAAATTTCCGGATCTGGGTAGCTTTAAAGCCGAT<br>CTGGATTTTCTGCTGCGTAATCTGTGGAAAGTTTGGCGTGAAACCATTTGTGGTGAAGCATTTCGTTGTGTTATTGCAGAAGCACAGC<br>TGGACCCTGCAACCCTGACCCAGCTGAAAGATCAGTTTATGGAACGTCGTCGTGAGATGCCGAAAAAACTGGTTGAAAATGCCATTAG<br>CAATGGTGAACTGCCGAAAGATACCAATCGTGAACTGCTGCTGGATATGATTTTTGGTTTTTGTTGGTATCGCCTGCTGACCGAACAG<br>CTGACCGTTGAACAGGATATTGAAGAATTTACCTTCCTGCTGATTAATGGTGTTTGTCCGGGTACACAGCGTTAA

srpR	Gene	ATGGCACGTAAAACCGCAGCAGAAGCAGAAGAAACCCGTCAGCGTATTATTGATGCAGCACTGGAAGTTTTTGTTGCACAGGGTGTTA<br>GTGATGCAACCCTGGATCAGATTGCACGTAAAGCCGGTGTTACCCGTGGTGCAGTTTATTGGCATTTTAATGGTAAACTGGAAGTTCT<br>GCAGGCAGTTCTGGCAAGCCGTCAGCATCCGCTGGAACTGGATTTTACACCGGATCTGGGTATTGAACGTAGCTGGGAAGCAGTTGTT<br>GTTGCAATGCTGGATGCAGTTCATAGTCCGCAGAGCAAACAGTTTAGCGAAATTCTGATTTATCAGGGTCTGGATGAAAGCGGTCTGA<br>TTCATAATCGTATGGTTCAGGCAAGCGATCGTTTTCTGCAGTATATTCATCAGGTTCTGCGTCATGCAGTTACCCAGGGTGAACTGCC<br>GATTAATCTGGATCTGCAGACCAGCATTGGTGTTTTTAAAGGTCTGATTACCGGTCTGCTGTATGAAGGTCTGCGTAGCAAAGATCAG<br>CAGGCACAGATTATCAAAGTTGCACTGGGTAGCTTTTGGGCACTGCTGCGTGAACCGCCTCGTTTTCTGCTGTGTGAAGAAGCACAGA<br>TTAAACAGGTGAAATCCTTCGAATAA
vanR	Gene	ATGGACATGCCTCGTATTAAACCGGGTCAGCGTGTTATGATGGCACTGCGTAAAATGATTGCAAGCGGTGAAATCAAAAGTGGTGAAC<br>GTATTGCAGAAATTCCGACCGCAGCAGCACTGGGTGTTAGCCGTATGCCGGTTCGTATCGCACTGCGTTCACTGGAACAAGAAGGTCT<br>GGTTGTTCGTCTGGGTGCACGTGGTTATGCAGCCCGTGGTGTTAGCAGCGATCAGATTCGTGATGCAATTGAAGTTCGTGGTGTTCTG<br>GAAGGTTTTGCAGCACGTCGTCTGGCAGAACGTGGTATGACCGCAGAAACCCATGCACGTTTTGTTGTACTGATTGCAGAAGGTGAAG<br>CACTGTTTGCAGCCGGTCGCCTGAATGGTGAAGATCTGGATCGTTATGCCGCATATAATCAGGCATTTCATGATACCCTGGTTAGCGC<br>AGCAGGTAATGGTGCAGTTGAAAGCGCACTGGCACGTAATGGTTTTGAACCGTTTGCAGCAGCCGGTGCACTGGCCCTGGATCTGATG<br>GACCTGTCTGCCGAATATGAACATCTGCTGGCAGCACATCGTCAGCATCAGGCAGTTCTGGATGCAGTTAGCTGTGGTGATGCCGAAG<br>GTGCAGAACGTATTATGCGTGATCATGCACTGGCAGCAATTCGTAATGCAAAAGTTTTTGAAGCAGCAGCAAGCGCAGGCGCACCGCT<br>GGGTGCAGCATGGTCAATTCGTGCAGATTGATAA
luxR	Gene	ATGAAAAACATAAATGCCGACGACACATACAGAATAATTAATAAAATTAAAGCTTGTAGAAGCAATAATGATATTAATCAATGCTTAT<br>CTGATATGACTAAAATGGTACATTGTGAATATTATTTACTCGCAATCATTTATCCTCATTATATGGTTAAATCCGATATTTCAATCCT<br>AGATAATTACCCTAAAAAATGGAGGCAATATTATGATGACGCTAATTTAATAAAATATGATCCTATAGTAGATTATTCTAACTCCAAT<br>CATTCACCAATTAATTGGAATATATTTGAAAACAATGCTGTAAATAAAAAATCTCCAAATGTAATTAAAGAAGCGAAAACATCAGGTC<br>TTATCACTGGGTTTAGTTTCCCTATTCATACGGCTAACAATGGCTTCGGAATGCTTAGTTTTGCACATTCAGAAAAAGACAACTATAT<br>AGATAGTTTATTTTTACATGCGTGTATGAACATACCATTAATTGTTCCTTCTCTAGTTGATAATTATCGAAAAATAAATATAGCAAAT<br>AATAAATCAAACAACGATTTAACCAAAAGAGAAAAAGAATGTTTAGCGTGGGCATGCGAAGGAAAAAGCTCTTGGGATATTTCAAAAA<br>TATTAGGTTGCAGTGAGCGTACTGTCACTTTCCATTTAACCAATGCGCAAATGAAACTCAATACAACAAACCGCTGCCAAAGTATTTC<br>TAAAGCAATTTTAACAGGAGCAATTGATTGCCCATACTTTAAAAATTAA
*araC**	Gene	ATGGCTGAAGCGCAAAATGATCCCCTGCTGCCGGGATACTCGTTTAATGCCCATCTGGTGGCGGGTTTAACGCCGATTGAGGCCAACG<br>GTTATCTCGATTTTTTTATCGACCGACCGCTGGGAATGAAAGGTTATATTCTCAATCTCACCATTCGCGGTCAGGGGGTGGTGAAAAA<br>TCAGGGACGAGAATTTGTTTGCCGACCGGGTGATATTTTGCTGTTCCCGCCAGGAGAGATTCATCACTACGGTCGTCATCCGGAGGCT<br>CGCGAATGGTATCACCAGTGGGTTTACTTTCGTCCGCGCGCCTACTGGCATGAATGGCTTAACTGGCCGTCAATATTTGCCAATACGG<br>GGTTCTTTCGCCCGGATGAAGCGCACCAGCCGCATTTCAGCGACCTGTTTGGGCAAATCATTAACGCCGGGCAAGGGGAAGGGCGCTA<br>TTCGGAGCTGCTGGCGATAAATCTGCTTGAGCAATTGTTACTGCGGCGCATGGAAGCGATTAACGAGTCGCTCCATCCACCGATGGAT<br>AATCGGGTACGCGAGGCTTGTCAGTACATCAGCGATCACCTGGCAGACAGCAATTTTGATATCGCCAGCGTCGCACAGCATGTTTGCT<br>TGTCGCCGTCGCGTCTGTCACATCTTTTCCGCCAGCAGTTAGGGATTAGCGTCTTAAGCTGGCGCGAGGACCAACGTATCAGCCAGGC<br>GAAGCTGCTTTTGAGCACCACCCGGATGCCTATCGCCACCGTCGGTCGCAATGTTGGTTTTGACGATCAACTCTATTTCTCGCGGGTA<br>TTTAAAAAATGCACCGGGGCCAGCCCGAGCGAGTTCCGTGCCGGTTAA
lacI	Gene	ATGAAACCAGTAACGTTATACGATGTCGCAGAGTATGCCGGTGTCTCTTATCAGACCGTTTCCCGCGTGGTGAACCAGGCCAGCCACG<br>TTTCTGCGAAAACGCGGGAAAAAGTGGAAGCGGCGATGGCGGAGCTGAATTACATTCCCAACCGCGTGGCACAACAACTGGCGGGCAA<br>ACAGTCGTTGCTGATTGGCGTTGCCACCTCCAGTCTGGCCCTGCACGCGCCGTCGCAAATTGTCGCGGCGATTAAATCTCGCGCCGAT<br>CAACTGGGTGCCAGCGTGGTGGTGTCGATGGTAGAACGAAGCGGCGTCGAAGCCTGTAAAGCGGCGGTGCACAATCTTCTCGCGCAAC<br>GCGTCAGTGGGCTGATCATTAACTATCCGCTGGATGACCAGGATGCCATTGCTGTGGAAGCTGCCTGCACTAATGTTCCGGCGTTATT<br>TCTTGATGTCTCTGACCAGACACCCATCAACAGTATTATTTTCTCCCATGAGGACGGTACGCGACTGGGCGTGGAGCATCTGGTCGCA<br>TTGGGTCACCAGCAAATCGCGCTGTTAGCGGGCCCATTAAGTTCTGTCTCGGCGCGTCTGCGTCTGGCTGGCTGGCATAAATATCTCA<br>CTCGCAATCAAATTCAGCCGATAGCGGAACGGGAAGGCGACTGGAGTGCCATGTCCGGTTTTCAACAAACCATGCAAATGCTGAATGA<br>GGGCATCGTTCCCACTGCGATGCTGGTTGCCAACGATCAGATGGCGCTGGGCGCAATGCGCGCCATTACCGAGTCCGGGCTGCGCGTT<br>GGTGCGGATATCTCGGTAGTGGGATACGACGATACCGAAGATAGCTCATGTTATATCCCGCCGTTAACCACCATCAAACAGGATTTTC<br>GCCTGCTGGGGCAAACCAGCGTGGACCGCTTGCTGCAACTCTCTCAGGGCCAGGCGGTGAAGGGCAATCAGCTGTTGCCAGTCTCACT<br>GGTTAAAAGAAAAACCACCCTGGCGCCCAATACGCAAACCGCCTCTCCCCGCGCGTTGGCCGATTCATTAATGCAGCTGGCACGACAG<br>GTTTCCCGACTGGAAAGCGGGCAGTGATAA
tetR	Gene	ATGTCCAGATTAGATAAAAGTAAAGTGATTAACAGCGCATTAGAGCTGCTTAATGAGGTCGGAATCGAAGGTTTAACAACCCGTAAAC<br>TCGCCCAGAAGCTAGGTGTAGAGCAGCCTACATTGTATTGGCATGTAAAAAATAAGCGGGCTTTGCTCGACGCCTTAGCCATTGAGAT<br>GTTAGATAGGCACCATACTCACTTTTGCCCTTTAGAAGGGGAAAGCTGGCAAGATTTTTTACGTAATAACGCTAAAAGTTTTAGATGT<br>GCTTTACTAAGTCATCGCGATGGAGCAAAAGTACATTTAGGTACACGGCCTACAGAAAAACAGTATGAAACTCTCGAAAATCAATTAG<br>CCTTTTTATGCCAACAAGGTTTTTCACTAGAGAATGCATTATATGCACTCAGCGCTGTGGGGCATTTTACTTTAGGTTGCGTATTGGA<br>AGATCAAGAGCATCAAGTCGCTAAAGAAGAAAGGGAAACACCTACTACTGATAGTATGCCGCCATTATTACGACAAGCTATCGAATTA<br>TTTGATCACCAAGGTGCAGAGCCAGCCTTCTTATTCGGCCTTGAATTGATCATATGCGGATTAGAAAAACAACTTAAATGTGAAAGTG<br>GGTCCTAA
cinR	Gene	ATGATTGAGAATACCTATAGCGAAAAGTTCGAGTCCGCGTTCGAACAGATCAAAGCGGCGGCCAACGTGGATGCCGCCATCCGTATTC<br>TCCAGGCGGAATATAACCTCGATTTCGTCACCTACCATCTCGCCCAGACAATCGCGAGCAAGATCGATTCGCCCTTCGTGCGCACCAC<br>CTATCCGGATGCCTGGGTTTCCCGTTACCTCCTCAACTGCTATGTGAAGGTCGATCCGATCATCAAGCAGGGCTTCGAACGCCAGCTG<br>CCCTTCGACTGGAGCGAGGTCGAACCGACGCCGGAGGCCTATGCCATGCTGGTCGACGCCCAGAAACACGGCATCGATGACAATGGCT

		ACTCCATCCCCGTCGCCGACAAGGCGCAGCGCCGCGCCCTGCTGTCGCTGAATGCCCATATACCGGCCGACGAATGGACCGAGCTCGT<br>GCGCCGCTGCCGCAATGAGTGGATCGAGATCGCCCATCTGATCCACCGCAAGGCCGTATATGAGCTGCATGGCGAAAACGATCCGGTG<br>CCGGCATTGTCGCCGCGCGAGATCGAGTGTCTGCACTGGACCGCCCTCGGCAAGGATTACAAGGATATTTCGGTCATCCTGGGCATAT<br>CAGAGCATACCACACGCGATTACCTGAAAACCGCCCGCTTCAGGCTCGGCTGCACCACGATCTCGGCCGCCGCGTCGCGGGCTGTTCA<br>ATTGCGCATCATCAATCCCTATAGGATCCGCATGACGCGACGTAATTGGTAATAG
ampR	Gene	ATGAGTATTCAACATTTCCGTGTCGCCCTTATTCCCTTTTTTGCGGCATTTTGCCTTCCTGTTTTTGCTCACCCAGAAACGCTGGTGA<br>AAGTAAAAGATGCTGAAGATCAGTTGGGTGCACGAGTGGGTTATATCGAACTGGATCTCAACAGCGGTAAGATCCTTGAGAGTTTTCG<br>CCCCGAAGAACGTTTTCCAATGATGAGCACTTTTAAAGTTCTGCTATGTGGCGCGGTATTATCCCGTATTGACGCCGGGCAAGAGCAA<br>CTCGGTCGCCGCATACACTATTCTCAGAATGACTTGGTTGAGTACTCACCAGTCACAGAAAAGCATCTTACGGATGGCATGACAGTAA<br>GAGAATTATGCAGTGCTGCCATAACCATGAGTGATAACACTGCGGCCAACTTACTTCTGACAACGATCGGAGGACCGAAGGAGCTAAC<br>CGCTTTTTTGCACAACATGGGGGATCATGTAACTCGCCTTGATCGTTGGGAACCGGAGCTGAATGAAGCCATACCAAACGACGAGCGT<br>GACACCACGATGCCTGTAGCAATGGCAACAACGTTGCGCAAACTATTAACTGGCGAACTACTTACTCTAGCTTCCCGGCAACAATTAA<br>TAGACTGGATGGAGGCGGATAAAGTTGCAGGACCACTTCTGCGCTCGGCCCTTCCGGCTGGCTGGTTTATTGCTGATAAATCTGGAGC<br>CGGTGAGCGTGGTTCTCGCGGTATCATTGCAGCACTGGGGCCAGATGGTAAGCCCTCCCGTATCGTAGTTATCTACACGACGGGGAGT<br>CAGGCAACTATGGATGAACGAAATAGACAGATCGCTGAGATAGGTGCCTCACTGATTAAGCATTGGTAA
kanR	Gene	ATGAGCCATATTCAACGGGAAACGTCTTGCTCGAGGCCGCGATTAAATTCCAACATGGATGCTGATTTATATGGGTATAAATGGGCTC<br>GCGATAATGTCGGGCAATCAGGTGCGACAATCTATCGATTGTATGGGAAGCCCGATGCGCCAGAGTTGTTTCTGAAACATGGCAAAGG<br>TAGCGTTGCCAATGATGTTACAGATGAGATGGTCAGACTAAACTGGCTGACGGAATTTATGCCTCTTCCGACCATCAAGCATTTTATC<br>CGTACTCCTGATGATGCATGGTTACTCACCACTGCGATCCCCGGGAAAACAGCATTCCAGGTATTAGAAGAATATCCTGATTCAGGTG<br>AAAATATTGTTGATGCGCTGGCAGTGTTCCTGCGCCGGTTGCATTCGATTCCTGTTTGTAATTGTCCTTTTAACAGCGATCGCGTATT<br>TCGTCTCGCTCAGGCGCAATCACGAATGAATAACGGTTTGGTTGATGCGAGTGATTTTGATGACGAGCGTAATGGCTGGCCTGTTGAA<br>CAAGTCTGGAAAGAAATGCATAAGCTTTTGCCATTCTCACCGGATTCAGTCGTCACTCATGGTGATTTCTCACTTGATAACCTTATTT<br>TTGACGAGGGGAAATTAATAGGTTGTATTGATGTTGGACGAGTCGGAATCGCAGACCGATACCAGGATCTTGCCATCCTATGGAACTG<br>CCTCGGTGAGTTTTCTCCTTCATTACAGAAACGGCTTTTTCAAAAATATGGTATTGATAATCCTGATATGAATAAATTGCAGTTTCAT<br>TTGATGCTCGATGAGTTTTTCTAA
*kanR**	Gene	ATGATTGAACAAGATGGATTGCACGCAGGTTCTCCGGCCGCTTGGGTGGAGAGGCTATTCGGCTATGACTGGGCACAACAGACAATCG<br>GCTGCTCTGATGCCGCCGTGTTCCGGCTGTCAGCGCAGGGGCGCCCGGTTCTTTTTGTCAAGACCGACCTGTCCGGTGCCCTGAATGA<br>ACTGCAGGACGAGGCAGCGCGGCTATCGTGGCTGGCCACGACGGGCGTTCCTTGCGCAGCTGTGCTCGACGTTGTCACTGAAGCGGGA<br>AGGGACTGGCTGCTATTGGGCGAAGTGCCGGGGCAGGATCTCCTGTCATCTCGCCTTGCTCCTGCCGAGAAAGTATCCATCATGGCTG<br>ATGCAATGCGGCGGCTGCATACGCTTGATCCGGCTACCTGCCCATTCGACCACCAAGCGAAACATCGCATCGAGCGAGCACGTACTCG<br>GATGGAAGCCGGTCTTGTCGATCAGGATGATCTGGACGAAGAGCATCAGGGGCTCGCGCCAGCCGAACTGTTCGCCAGGCTCAAGGCG<br>CGCATGCCCGACGGCGAGGATCTCGTCGTGATCCATGGCGATGCCTGCTTGCCGAATATCATGGTGGAAAATGGCCGCTTTTCTGGAT<br>TCAACGACTGTGGCCGGCTGGGTGTGGCGGACCGCTATCAGGACATAGCGTTGGATACCCGTGATATTGCTGAAGAGCTTGGCGGCGA<br>ATGGGCTGACCGCTTCCTCGTGCTTTACGGTATCGCCGCTCCCGATTCGCAGCGCATCGCCTTCTATCGCCTTCTTGACGAGTTCTTC<br>TGA
L3S3P31	Terminator	CCAATTATTGAACACCCTAACGGGTGTTTTTTTTTTTTTGGTCTCCC
L3S2P55	Terminator	CTCGGTACCAAAGACGAACAATAAGACGCTGAAAAGCGTCTTTTTTCGTTTTGGTCC
L3S3P11	Terminator	CCAATTATTGAACACCCTTCGGGGTGTTTTTTTGTTTCTGGTCTCCC
L3S2P11	Terminator	CTCGGTACCAAATTCCAGAAAAGAGACGCTTTCGAGCGTCTTTTTTCGTTTTGGTCC
L3S2P42	Terminator	CTCGGTACCAAAGAAAAATAAAAAGACGCTGAAAAGCGTCTTTTTATTTTTCGGTCC
ECK120010799	Terminator	GTTATGAGTCAGGAAAAAAGGCGACAGAGTAATCTGTCGCCTTTTTTCTTTGCTTGCTTT
ECK120010876	Terminator	TAAGGTTGAAAAATAAAAACGGCGCTAAAAAGCGCCGTTTTTTTTGACGGTGGTA
ECK120033737	Terminator	GGAAACACAGAAAAAAGCCCGCACCTGACAGTGCGGGCTTTTTTTTTCGACCAAAGG
ECK120029600	Terminator	TTCAGCCAAAAAACTTAAGACCGCCGGTCTTGTCCACTACCTTGCAGTAATGCGGTGGACAGGATCGGCGGTTTTCTTTTCTCTTCTC<br>AA
ECK120033736	Terminator	AACGCATGAGAAAGCCCCCGGAAGATCACCTTCCGGGGGCTTTTTTATTGCGC
L3S2P21	Terminator	CTCGGTACCAAATTCCAGAAAAGAGGCCTCCCGAAAGGGGGGCCTTTTTTCGTTTTGGTCC
L3S3P21	Terminator	CCAATTATTGAAGGCCTCCCTAACGGGGGGCCTTTTTTTGTTTCTGGTCTCCC

A	Scar	GGAG
B	Scar	TACG
B’	Scar	TACT
C	Scar	AATG
D	Scar	AGGT
E	Scar	GCTT
F	Scar	CGCT
U	Scar	GGGC
V	Scar	TCTG
X	Scar	TGTC
p15A	Origin	GACCTCAGCGCTAGCGGAGTGTATACTGGCTTACTATGTTGGCACTGATGAGGGTGTCAGTGAAGTGCTTCATGTGGCAGGAGAAAAA<br>AGGCTGCACCGGTGCGTCAGCAGAATATGTGATACAGGATATATTCCGCTTCCTCGCTCACTGACTCGCTACGCTCGGTCGTTCGACT<br>GCGGCGAGCGGAAATGGCTTACGAACGGGGCGGAGATTTCCTGGAAGATGCCAGGAAGATACTTAACAGGGAAGTGAGAGGGCCGCGG<br>CAAAGCCGTTTTTCCATAGGCTCCGCCCCCCTGACAAGCATCACGAAATCTGACGCTCAAATCAGTGGTGGCGAAACCCGACAGGACT<br>ATAAAGATACCAGGCGTTTCCCCCTGGCGGCTCCCTCGTGCGCTCTCCTGTTCCTGCCTTTCGGTTTACCGGTGTCATTCCGCTGTTA<br>TGGCCGCGTTTGTCTCATTCCACGCCTGACACTCAGTTCCGGGTAGGCAGTTCGCTCCAAGCTGGACTGTATGCACGAACCCCCCGTT<br>CAGTCCGACCGCTGCGCCTTATCCGGTAACTATCGTCTTGAGTCCAACCCGGAAAGACATGCAAAAGCACCACTGGCAGCAGCCACTG<br>GTAATTGATTTAGAGGAGTTAGTCTTGAAGTCATGCGCCGGTTAAGGCTAAACTGAAAGGACAAGTTTTGGTGACTGCGCTCCTCCAA<br>GCCAGTTACCTCGGTTCAAAGAGTTGGTAGCTCAGAGAACCTTCGAAAAACCGCCCTGCAAGGCGGTTTTTTCGTTTTCAGAGCAAGA<br>GATTACGCGCAGACCAAAACGATCTCAAGAAGATCATCTTATTAA
p15A*	Origin	GCTAGCGGAGTGTATACTGGCTTACTATGTTGGCACTGATGAGGGTGTCAGTGAAGTGCTTCATGTGGCAGGAGAAAAAAGGCTGCAC<br>CGGTGCGTCAGCAGAATATGTGATACAGGATATATTCCGCTTCCTCGCTCACTGACTCGCTACGCTCGGTCGTTCGACTGCGGCGAGC<br>GGAAATGGCTTACGAACGGGGCGGAGATTTCCTGGAAGATGCCAGGAAGATACTTAACAGGGAAGTGAGAGGGCCGCGGCAAAGCCGT<br>TTTTCCATAGGCTCCGCCCCCCTGACAAGCATCACGAAATCTGACGCTCAAATCAGTGGTGGCGAAACCCGACAGGACTATAAAGATA<br>CCAGGCGTTTCCCCCTGGCGGCTCCCTCGTGCGCTCTCCTGTTCCTGCCTTTCGGTTTACCGGTGTCATTCCGCTGTTATGGCCGCGT<br>TTGTCTCATTCCACGCCTGACACTCAGTTCCGGGTAGGCAGTTCGCTCCAAGCTGGACTGTATGCACGAACCCCCCGTTCAGTCCGAC<br>CGCTGCGCCTTATCCGGTAACTATCGTCTTGAGTCCAACCCGGAAAGACATGCAAAAGCACCACTGGCAGCAGCCACTGGTAATTGAT<br>TTAGAGGAGTTAGTCTTGAAGTCATGCGCCGGTTACGGCTAAACTGAAAGGACAAGTTTTGGTGACTGCGCTCCTCCAAGCCAGTTAC<br>CTCGGTTCAAAGAGTTGGTAGCTCAGAGAACCTTCGAAAAACCGCCCTGCAAGGCGGTTTTTTCGTTTTCAGAGCAAGAGATTACGCG<br>CAGACCAAAACGATCTCAAGAAGATCATCTTATTAA
pSC101 var2	Origin	AGTAAGACGGGTAAGCCTGTTGATGATACCGCTGCCTTACTGGGTGCATTAGCCAGTCTGAATGACCTGTCACGGGATAATCCGAAGT<br>GGTCAGACTGGAAAATCAGAGGGCAGGAACTGCTGAACAGCAAAAAGTCAGATAGCACCACATAGCAGACCCGCCATAAAACGCCCTG<br>AGAAGCCCGTGACGGGCTTTTCTTGTATTATGGGTAGTTTCCTTGCATGAATCCATAAAAGGCGCCTGTAGTGCCATTTACCCCCATT<br>CACTGCCAGAGCCGTGAGCGCAGCGAACTGAATGTCACGAAAAAGACAGCGACTCAGGTGCCTGATGGTCGGAGACAAAAGGAATATT<br>CAGCGATTTGCCCGAGCTTGCGAGGGTGCTACTTAAGCCTTTAGGGTTTTAAGGTCTGTTTTGTAGAGGAGCAAACAGCGTTTGCGAC<br>ATCCTTTTGTAATACTGCGGAACTGACTAAAGTAGTGAGTTATACACAGGGCTGGGATCTATTCTTTTTATCTTTTTTTATTCTTTCT<br>TTATTCTATAAATTATAACCACTTGAATATAAACAAAAAAAACACACAAAGGTCTAGCGGAATTTACAGAGGGTCTAGCAGAATTTAC<br>AAGTTTTCCAGCAAAGGTCTAGCAGAATTTACAGATACCCACAACTCAAAGGAAAAGGACTAGTAATTATCATTGACTAGCCCATCTC<br>AATTGGTATAGTGATTAAAATCACCTAGACCAATTGAGATGTATGTCTGAATTAGTTGTTTTCAAAGCAAATGAACTAGCGATTAGTC<br>GCTATGACTTAACGGAGCATGAAACCAAGCTAATTTTATGCTGTGTGGCACTACTCAACCCCACGATTGAAAACCCTACAAGGAAAGA<br>ACGGACGGTATCGTTCACTTATAACCAATACGCTCAGATGATGAACATCAGTAGGGAAAATGCTTATGGTGTATTAGCTAAAGCAACC<br>AGAGAGCTGATGACGAGAACTGTGGAAATCAGGAATCCTTTGGTTAAAGGCTTTTGGATTTTCCAGTGGACAAACTATGCCAAGTTCT<br>CAAGCGAAAAATTAGAATTAGTTTTTAGTGAAGAGATATTGCCTTATCTTTTCCAGTTAAAAAAATTCATAAAATATAATCTGGAACA<br>TGTTAAGTCTTTTGAAAACAAATACTCTATGAGGATTTATGAGTGGTTATTAAAAGAACTAACACAAAAGAAAACTCACAAGGCAAAT<br>ATAGAGATTAGCCTTGATGAATTTAAGTTCATGTTAATGCTTGAAAATAACTACCATGAGTTTAAAAGGCTTAACCAATGGGTTTTGA<br>AACCAATAAGTAAAGATTTAAACACTTACAGCAATATGAAATTGGTGGTTGATAAGCGAGGCCGCCCGACTGATACGTTGATTTTCCA<br>AGTTGAACTAGATAGACAAATGGATCTCGTAACCGAACTTGAGAACAACCAGATAAAAATGAATGGTGACAAAATACCAACAACCATT<br>ACATCAGATTCCTACCTACATAACGGACTAAGAAAAACACTACACGATGCTTTAACTGCAAAAATTCAGCTCACCAGTTTTGAGGCAA<br>AATTTTTGAGTGACATGCAAAGTAAGTATGATCTCAATGGTTCGTTCTCATGGCTCACGCAAAAACAACGAACCACACTAGAGAACAT<br>ACTGGCTAAATACGGAAGGATCTGAGGTTCTTATGGCTCTTGTATCTATCAGTGAAGCATCAAGACTAACAAACAAAAGTAGAACAAC<br>TGTTCACCGTTACATATCAAAGGGAAAACTGTCCATATGCACAGATGAAAACGGTGTAAAAAAGATAGATACATCAGAGCTTTTACGA<br>GTTTTTGGTGCATTCAAAGCTGTTCACCATGAACAGATCGACAATGTAACAGATGAACAGCATGTAACACCTAATAGAACAGGTGAAA<br>CCAGTAAAACAAAGCAACTAGAACATGAAATTGAACACCTGAGACAACTTGTTACAGCTCAACAGTCACACATAGACAGCCTGAAACA<br>GGCGATGCTGCTTATCGAATCAAAGCTGCCGACAACACGGGAGCCAGTGACGCCTCCCGTGGGGAAAAAATCATGGCAATTCTGGAAG

AAATAGCGCTTTCAGCCGGCAAACCGGCTGAAGCCGGATCTGCGATTCTGATAACAAACTAGCAACACCAGAACAGCCCGTTTGCGGG
CAGCAAAACCCGTAC

Appendix Table S5: Complete annotated sequences of the 7-segment circuits.

Circuit name	Type	DNA sequence[a]
Segment A	Circuit	GGAGTCGTCACTAGAGGGCGATAGTGACAAACTTGACAACTCATCACTTCCTACGTAGGCTGCTAGCCGCTCATTCACTAGGTCTGATTCGTTACCAATTGA<br>CAGCTGGTGGTCGAATCAAGATAATAGACCAGTCACTATATTTAGCGGTCAACGCATGTGCTTTGCGTTCTGATGAGACAGTGATGTCGAAACCGCCTCTAC<br>AAATAATTTTGTTTAAGGAGCTATGGACTATGTTTGAAAGGCTGAAATACTAGATGGCACGTACCCCGAGCCGTAGCAGCATTGGTAGCCTGCGTAGTCCGC<br>ATACCCATAAAGCAATTCTGACCAGCACCATTGAAATCCTGAAAGAATGTGGTTATAGCGGTCTGAGCATTGAAAGCGTTGCACGTCGTGCCGGTGCAAGCA<br>AACCGACCATTTATCGTTGGTGGACCAATAAAGCAGCACTGATTGCCGAAGTGTATGAAAATGAAAGCGAACAGGTGCGTAAATTTCCGGATCTGGGTAGCT<br>TTAAAGCCGATCTGGATTTTCTGCTGCGTAATCTGTGGAAAGTTTGGCGTGAAACCATTTGTGGTGAAGCATTTCGTTGTGTTATTGCAGAAGCACAGCTGG<br>ACCCTGCAACCCTGACCCAGCTGAAAGATCAGTTTATGGAACGTCGTCGTGAGATGCCGAAAAAACTGGTTGAAAATGCCATTAGCAATGGTGAACTGCCGA<br>AAGATACCAATCGTGAACTGCTGCTGGATATGATTTTTGGTTTTTGTTGGTATCGCCTGCTGACCGAACAGCTGACCGTTGAACAGGATATTGAAGAATTTA<br>CCTTCCTGCTGATTAATGGTGTTTGTCCGGGTACACAGCGTTAAGGAAACACAGAAAAAAGCCCGCACCTGACAGTGCGGGCTTTTTTTTTTCGACCAAAGG<br>TACGAGCGCGGGTGAGAGGGATTCGTTACCAATAGACAATTGATTGGACGTTCAATATAATGCTAGCATAGCTTCTTACCGGACCTGTAGGATCGTACAGGT<br>TTACGCAAGAAAATGGTTTGTTATTTTCGAATAAAAGGGGTCAGTTGATGTGCTTTCAACTCTGATGAGTCAGTGATGACGAAACCCCCTCTACAAATAATT<br>TTGTTTAACTATGGACTATGTTTTCACATACGAGGGGGATTAGATGAACAAAACCATTGATCAGGTGCGTAAAGGTGATCGTAAAAGCGATCTGCCGGTTCG<br>TCGTCGTCCGCGTCGTAGTGCCGAAGAAACCCGTCGTGATATTCTGGCAAAAGCCGAAGAACTGTTTCGTGAACGTGGTTTTAATGCAGTTGCCATTGCAGA<br>TATTGCAAGCGCACTGAATATGAGTCCGGCAAATGTGTTTAAACATTTTAGCAGCAAAAACGCACTGGTTGATGCAATTGGTTTTGGTCAGATTGGTGTTTT<br>TGAACGTCAGATTTGTCCGCTGGATAAAAGCCATGCACCGCTGGATCGTCTGCGTCATCTGGCACGTAATCTGATGGAACAGCATCATCAGGATCATTTCAA<br>ACACATACGGGTTTTTATTCAGATCCTGATGACCGCCAAACAGGATATGAAATGTGGCGATTATTACAAAAGCGTGATTGCAAAACTGCTGGCCGAAATTAT<br>TCGTGATGGTGTTGAAGCAGGTCTGTATATTGCAACCGATATTCCGGTTCTGGCAGAAACCGTTCTGCATGCACTGACCAGCGTTATTCATCCGGTTCTGAT<br>TGCACAAGAAGATATTGGTAATCTGGCAACCCGTTGTGATCAGCTGGTTGATCTGATTGATGCAGGTCTGCGTAATCCGCTGGCAAAATAACCAATTATTGA<br>ACACCCTAACGGGTGTTTTTTTTTTTTTGGTCTCCCAGGTCCCTTTGTGCGTCCAAACGGACGCACGGCGCTCTAAAGCGGGTCGCGATCTTTCAGATTCGC<br>TCCTCGCGCTTTCAGTCTTTGTTTTGGCGCATGTCGTTATCGCAAAACCGCTGCACACTTTTGCGCGACATGCTCTGATCCCCCTCATCTGGGGGGGCCTAT<br>CTGAGGGAATTTCCGATCCGGCTCGCCTGAACCATTCTGCTTTCCACGAACTTGAAAACGCTAGCGCGGGTGAGAGGGATTCGTTACCAATAGACAATTGAT<br>TGGACGTTCAATATAATGCTAGCAGGAGTCAATTAATGTGCTTTTAATTCTGATGAGACGGTGACGTCGAAACTCCCTCTACAAATAATTTTGTTTAATACG<br>CTATGGACTATGTTTTCTGCTATGGACTATGTTTTCACACACGAGATGCCTCGATGAGCTATGGTGATAGCCGTGAAAAAATTCTGAGCGCAGCAACCCGTC<br>TGTTTCAGCTGCAGGGTTATTATGGCACCGGTCTGAATCAGATTATCAAAGAAAGCGGTGCACCGAAAGGTAGCCTGTATTATCATTTTCCGGGTGGTAAAG<br>AACAGCTGGCAATTGAAGCAGTGAACGAAATGAAAGAATATATCCGCCAGAAAATCGCCGATTGTATGGAAGCATGTACCGATCCGGCAGAAGGTATTCAGG<br>CATTTCTGAAAGAACTGAGCTGTCAGTTTAGCTGTACCGAAGATATTGAAGGTCTGCCGGTTGGTCTGCTGGCAGCAGAAACCAGCCTGAAAAGCGAACCGC<br>TGCGTGAAGCATGTCATGAAGCATATAAAGAATGGGCCAGCGTGTATGAAGAAAAACTGCGTCAGACCGGTTGTAGCGAAAGCCGTGCAAAAGAAGCAAGCA<br>CCGTTGTTAATGCAATGATTGAAGGTGGTATTCTGCTGAGCCTGACCGCAAAAAATAGCACACCGCTGCTGCATATTAGCAGCTGTATTCCGGATCTGCTGA<br>AACGTTAATAAGGTTGAAAAATAAAAACGGCGCTAAAAAGCGCCGTTTTTTTTGACGGTGGTAGCTTTCGTGTAAGTAGCGTAACAAACAGACAATCTGGTC<br>TGTTTGTATTATGGAAAATTTTTCTGTATAATAGATTCAACAAACAGACAATCTGGTCTGTTTGTATTATAGTAGTCACCGGCTGTGCTTGCCGGTCTGATG<br>AGCCTGTGAAGGCGAAACTACCTCTACAAATAATTTTGTTTAAACCCCCGAGATGAAATACATCCTGTTTGAGGTGTGCGAAATGGGTAAAAGCCGTGAACA<br>GACCATGGAAAATATTCTGAAAGCAGCCAAAAAGAAATTCGGCGAACGTGGTTATGAAGGCACCAGCATTCAAGAAATTACCAAAGAAGCCAAAGTTAACGT<br>TGCAATGGCCAGCTATTACTTTAATGGCAAAGAGAACCTGTACTACGAGGTGTTCAAAAAATACGGTCTGGCAAATGAACTGCCGAACTTTCTGGAAAAAAA<br>CCAGTTTAATCCGATTAATGCCCTGCGTGAATATCTGACCGTTTTTACCACCCACATTAAAGAAAATCCGGAAATTGGCACCCTGGCCTATGAAGAAATTAT<br>CAAAGAAAGCGCACGCCTGGAAAAAATCAAACCGTATTTTATCGGCAGCTTCGAACAGCTGAAAGAAATTCTGCAAGAGGGTGAAAAACAGGGTGTGTTTCA<br>CTTTTTTAGCATCAACCATACCATCCATTGGATTACCAGCATTGTTCTGTTTCCGAAATTCAAAAAATTCATCGATAGCCTGGGTCCGAATGAAACCAATGA<br>TACCAATCATGAATGGATGCCGGAAGATCTGGTTAGCCGTATTATTAGCGCACTGACCGATAAACCGAACATTTAAGTTATGAGTCAGGAAAAAAGGCGACA<br>GAGTAATCTGTCGCCTTTTTCTTTGCTTGCTTTCGCTCCCTTTGTGCGTCCAAACGGACGCACGGCGCTCTAAAGCGGGTCGCGATCTTTCAGATTCGCTC<br>CTCGCGCTTTCAGTCTTTGTTTTGGCGCATGTCGTTATCGCAAAACCGCTGCACACTTTTGCGCGACATGCTCTGATCCCCCTCATCTGGGGGGGCCTATCT<br>GAGGGAATTTCCGATCCGGCTCGCCTGAACCATTCTGCTTTCCACGAACTTGAAAACGCTATAGCTTCTTACCGGACCTGTAGGATCGTACAGGTTTACGCA<br>AGAAAATGGTTTGTTATTTTCGAATAAAAGAAGTCAATTAATGTGCTTTTAATTCTGATGAGTCGGTGACGACGAAACTTCCTCTACAAATAATTTTGTTTA<br>ACCCCCCGAGGAGTAGCACATGCCGAAACTGGGTATGCAGAGCATTCGTCGTCGTCAGCTGATTGATGCAACCCTGGAAGCAATTAATGAAGTTGGTATGCA<br>TGATGCAACCATTGCACAGATTGCACGTCGTGCCGGTGTTAGCACCGGTATTATTAGCCATTATTTCCGCGATAAAAACGGTCTGCTGGAAGCAACCATGCG<br>TGATATTACCAGCCAGCTGCGTGATGCAGTTCTGAATCGTCTGCATGCACTGCCGCAGGGTAGCGCAGAACAGCGTCTGCAGGCAATTGTTGGTGGTAATTT<br>TGATGAAACCCAGGTTAGCAGCGCAGCAATGAAAGCATGGCTGGCATTTTGGGCAAGCAGCATGCATCAGCCGATGCTGTATCGTCTGCAGCAGGTTAGCAG<br>TCGTCGTCTGCTGAGCAATCTGGTTAGCGAATTTCGTCGTGAACTGCCTCGTGAACAGGCACAAGAGGCAGGTTATGGTCTGGCAGCACTGATTGATGGTCT<br>GTGGCTGCGTGCAGCACTGAGCGGTAAACCGCTGGATAAAACCCGTGCAAATAGCCTGACCCGTCATTTTATCACCCAGCATCTGCCGACCGATTAACCAAT<br>TATTGAACACCCTTCGGGGTGTTTTTTTGTTTCTGGTCTCCCTGTCAACGATCGTTGGCTGTGTTGACAATTAATCATCGGCTCGTATAATGTGTGGAATTG<br>TGAGCGCTCACAATTTACTCCACCGTTGGCTTTTTTCCCTATCAGTGATAGAGATTGACATCCCTATCAGTGATAGAGATAATGAGCACAGTACGTCTGAGC<br>GTGATACCCGCTCACTGAAGATGGCCCGGTAGGGCCGAAACGTACCTCTACAAATAATTTTGTTTAAGACTTAAACGATTGGCCGACCGCATGAGCCCGAAA<br>CGTCGTACCCAGGCAGAACGTGCAATGGAAACCCAGGGTAAACTGATTGCAGCAGCACTGGGTGTTCTGCGTGAAAAAGGTTATGCAGGTTTTCGTATTGCA<br>GATGTTCCGGGTGCAGCCGGTGTTAGCCGTGGTGCACAGAGCCATCATTTTCCGACCAAACTGGAACTGCTGCTGGCAACCTTTGAATGGCTGTATGAGCAG<br>ATTACCGAACGTAGCCGTGCACGTCTGGCAAAACTGAAACCGGAAGATGATGTTATTCAGCAGATGCTGGATGATGCAGCAGAATTTTTTCTGGATGATGAT<br>TTTAGCATCAGCCTGGATCTGATTGTTGCAGCAGATCGTGATCCGGCACTGCGTGAAGGTATTCAGCGTACCGTTGAACGTAATCGTTTTGTTGTTGAAGAT<br>ATGTGGCTGGGTGTGCTGGTGAGCCGTGGTCTGAGCCGTGATGATGCCGAAGATATTCTGTGGCTGATTTTTAACAGCGTTCGTGGTCTGGCAGTTCGTAGC<br>CTGTGGCAGAAAGATAAAGAACGTTTTGAACGTGTGCGTAATAGCACCCTGGAAATTGCACGTGAACGTTATGCAAAATTCAAACGTTGATAACTCGGTACC<br>AAAGAAAAATAAAAAGACGCTGAAAAGCGTCTTTTTATTTTTCGGTCCAATG
Segment B	Circuit	GGAGTCGTCACTAGAGGGCGATAGTGACAAACTTGACAACTCATCACTTCCTACGTAGGCTGCTAGCCTTGTCCAACCAAATGATTCGTTACCCTTTGACAG<br>TTTCTATCGATCTATAGATAATGCTAGCAGCGGTCAACGCATGTGCTTTGCGTTCTGATGAGACAGTGATGTCGAAACCGCCTCTACAAATAATTTTGTTTA<br>AGGAGCTATGGACTATGTTTGAAAGGCTGAAATACTAGATGGCACGTACCCCGAGCCGTAGCAGCATTGGTAGCCTGCGTAGTCCGCATACCCATAAAGCAA<br>TTCTGACCAGCACCATTGAAATCCTGAAAGAATGTGGTTATAGCGGTCTGAGCATTGAAAGCGTTGCACGTCGTGCCGGTGCAAGCAAACCGACCATTTATC<br>GTTGGTGGACCAATAAAGCAGCACTGATTGCCGAAGTGTATGAAAATGAAAGCGAACAGGTGCGTAAATTTCCGGATCTGGGTAGCTTTAAAGCCGATCTGG<br>ATTTTCTGCTGCGTAATCTGTGGAAAGTTTGGCGTGAAACCATTTGTGGTGAAGCATTTCGTTGTGTTATTGCAGAAGCACAGCTGGACCCTGCAACCCTGA<br>CCCAGCTGAAAGATCAGTTTATGGAACGTCGTCGTGAGATGCCGAAAAAACTGGTTGAAAATGCCATTAGCAATGGTGAACTGCCGAAAGATACCAATCGTG<br>AACTGCTGCTGGATATGATTTTTGGTTTTTGTTGGTATCGCCTGCTGACCGAACAGCTGACCGTTGAACAGGATATTGAAGAATTTACCTTCCTGCTGATTA<br>ATGGTGTTTGTCCGGGTACACAGCGTTAAGGAAACACAGAAAAAAGCCCGCACCTGACAGTGCGGGCTTTTTTTTTTCGACCAAAGGTACGTCGTGTAAGTA<br>GCGTAACAAACAGACAATCTGGTCTGTTTGTATTATGGAAAATTTTTCTGTATAATAGATTCAACAAACAGACAATCTGGTCTGTTTGTATTATATAGCTTC<br>TTACCGGACCTGTAGGATCGTACAGGTTTACGCAAGAAAATGGTTTGTTATTTTCGAATAAAAGGGTGTCTCAAGGTGCGTACCTTGACTGATGAGTCCGAA<br>AGGACGAAACACCCCTCTACAAATAATTTTGTTTAAAATGTTCCCTAATAATCAGCAAAGAGGTTACTAGATGGCAGGCGCAGTTGGTCGTCCGCGTCGTAG<br>TGCACCGCGTCGTGCAGGTAAAAATCCGCGTGAAGAAATTCTGGATGCAAGCGCAGAACTGTTTACCCGTCAGGGTTTTGCAACCACCAGTACCCATCAGAT<br>TGCAGATGCAGTTGGTATTCGTCAGGCAAGCCTGTATTATCATTTTCCGAGCAAAACCGAAATCTTTCTGACCCTGCTGAAAAGCACCGTTGAACCGAGCAC<br>CGTTCTGGCAGAAGATCTGAGCACCCTGGATGCAGGTCCGGAAATGCGTCTGTGGGCAATTGTTGCAAGCGAAGTTCGTCTGCTGCTGAGCACCAAATGGAA<br>TGTTGGTCGTCTGTATCAGCTGCCGATTGTTGGTAGCGAAGAATTTGCAGAATATCATAGCCAGCGTGAAGCACTGACCAATGTTTTTCGTGATCTGGCAAC<br>CGAAATTGTTGGTGATGATCCGCGTGCAGAACTGCCGTTTCATATTACCATGAGCGTTATTGAAATGCGTCGCAATGATGGTAAAATTCCGAGTCCGCTGAG<br>CGCAGATAGCCTGCCGGAAACCGCAATTATGCTGGCAGATGCAAGCCTGGCAGTTCTGGGTGCACCGCTGCCTGCAGATCGTGTTGAAAAAACCCTGGAACT<br>GATTAAACAGGCAGATGCAAAATAACTCGGTACCAAAGACGAACAATAAGACGCTGAAAAGCGTCTTTTTTCGTTTTGGTCCAGGTTCGTGTAAGTAGCGTA<br>ACAAACAGACAATCTGGTCTGTTTGTATTATGGAAAATTTTTCTGTATAATAGATTCAACAAACAGACAATCTGGTCTGTTTGTATTATTACTCCACCGTTG<br>GCTTTTTTCCCTATCAGTGATAGAGATTGACATCCCTATCAGTGATAGAGATAATGAGCACAGGGGTCAGTTGATGTGCTTTCAACTCTGATGAGTCAGTGA<br>TGACGAAACCCCCTCTACAAATAATTTTGTTTAACTATGGACTATGTTTTCACATACGAGGGGGATTAGATGAACAAAACCATTGATCAGGTGCGTAAAGGT<br>GATCGTAAAAGCGATCTGCCGGTTCGTCGTCGTCCGCGTCGTAGTGCCGAAGAAACCCGTCGTGATATTCTGGCAAAAGCCGAAGAACTGTTTCGTGAACGT<br>GGTTTTAATGCAGTTGCCATTGCAGATATTGCAAGCGCACTGAATATGAGTCCGGCAAATGTGTTTAAACATTTTAGCAGCAAAAACGCACTGGTTGATGCA<br>ATTGGTTTTGGTCAGATTGGTGTTTTTGAACGTCAGATTTGTCCGCTGGATAAAAGCCATGCACCGCTGGATCGTCTGCGTCATCTGGCACGTAATCTGATG<br>GAACAGCATCATCAGGATCATTTCAAACACATACGGGTTTTTATTCAGATCCTGATGACCGCCAAACAGGATATGAAATGTGGCGATTATTACAAAAGCGTG<br>ATTGCAAAACTGCTGGCCGAAATTATTCGTGATGGTGTTGAAGCAGGTCTGTATATTGCAACCGATATTCCGGTTCTGGCAGAAACCGTTCTGCATGCACTG<br>ACCAGCGTTATTCATCCGGTTCTGATTGCACAAGAAGATATTGGTAATCTGGCAACCCGTTGTGATCAGCTGGTTGATCTGATTGATGCAGGTCTGCGTAAT<br>CCGCTGGCAAAATAACCAATTATTGAACACCCTAACGGGTGTTTTTTTTTTTTTGGTCTCCCGCTTTACTCCACCGTTGGCTTTTTTCCCTATCAGTGATAG<br>AGATTGACATCCCTATCAGTGATAGAGATAATGAGCACATAGCTTCTTACCGGACCTGTAGGATCGTACAGGTTTACGCAAGAAAATGGTTTGTTATTTTCG<br>AATAAAAGTAGTCACCGGCTGTGCTTGCCGGTCTGATGAGCCTGTGAAGGCGAAACTACCTCTACAAATAATTTTGTTTAAACCCCCGAGATGAAATACATC<br>CTGTTTGAGGTGTGCGAAATGGGTAAAAGCCGTGAACAGACCATGGAAAATATTCTGAAAGCAGCCAAAAAGAAATTCGGCGAACGTGGTTATGAAGGCACC<br>AGCATTCAAGAAATTACCAAAGAAGCCAAAGTTAACGTTGCAATGGCCAGCTATTACTTTAATGGCAAAGAGAACCTGTACTACGAGGTGTTCAAAAAATAC

GGTCTGGCAAATGAACTGCCGAACTTTCTGGAAAAAAACCAGTTTAATCCGATTAATGCCCTGCGTGAATATCTGACCGTTTTTACCACCCACATTAAAGAA
AATCCGGAAATTGGCACCCTGGCCTATGAAGAAATTATCAAAGAAAGCGCACGCCTGGAAAAAATCAAACCGTATTTTATCGGCAGCTTCGAACAGCTGAAA
GAAATTCTGCAAGAGGGTGAAAAACAGGGTGTGTTTCACTTTTTTAGCATCAACCATACCATCCATTGGATTACCAGCATTGTTCTGTTTCCGAAATTCAAA
AAATTCATCGATAGCCTGGGTCCGAATGAAACCAATGATACCAATCATGAATGGATGCCGGAAGATCTGGTTAGCCGTATTATTAGCGCACTGACCGATAAA
CCGAACATTTAAGTTATGAGTCAGGAAAAAAAGGCGACAGAGTAATCTGTCGCCTTTTTTCTTTGCTTGCTTTCGCTCCCTTTGTGCGTCCAAACGGACGCAC
GGCGCTCTAAAGCGGGTCGCGATCTTTCAGATTCGCTCCTCGCGCTTTCAGTCTTTGTTTTGGCGCATGTCGTTATCGCAAAACCGCTGCACACTTTTGCGC
GACATGCTCTGATCCCCCTCATCTGGGGGGGCCTATCTGAGGGAATTTCCGATCCGGCTCGCCTGAACCATTCTGCTTTCCACGAACTTGAAAACGCTAGTA
CGTCTGAGCGTGATACCCGCTCACTGAAGATGGCCCGGTAGGGCCGAAACGTACCTCTACAAATAATTTTGTTTAAGACTTAAACGATTGGCCGACCGCATG
AGCCCGAAACGTCGTACCCAGGCAGAACGTGCAATGGAAACCCAGGGTAAACTGATTGCAGCAGCACTGGGTGTTCTGCGTGAAAAAGGTTATGCAGGTTTT
CGTATTGCAGATGTTCCGGGTGCAGCCGGTGTTAGCCGTGGTGCACAGAGCCATCATTTTCCGACCAAACTGGAACTGCTGCTGGCAACCTTTGAATGGCTG
TATGAGCAGATTACCGAACGTAGCCGTGCACGTCTGGCAAAACTGAAACCGGAAGATGATGTTATTCAGCAGATGCTGGATGATGCAGCAGAATTTTTTCTG
GATGATGATTTTAGCATCAGCCTGGATCTGATTGTTGCAGCAGATCGTGATCCGGCACTGCGTGAAGGTATTCAGCGTACCGTTGAACGTAATCGTTTTGTT
GTTGAAGATATGTGGCTGGGTGTGCTGGTGAGCCGTGGTCTGAGCCGTGATGATGCCGAAGATATTCTGTGGCTGATTTTTAACAGCGTTCGTGGTCTGGCA
GTTCGTAGCCTGTGGCAGAAAGATAAAGAACGTTTTGAACGTGTGCGTAATAGCACCCTGGAAATTGCACGTGAACGTTATGCAAAATTCAAACGTTGATAA
CTCGGTACCAAAGAAAAATAAAAAGACGCTGAAAAGCGTCTTTTTATTTTTCGGTCCAATG

GGAGTCTATGATTGGTCCAGATTCGTTACCAATTGACAGCTAGCTCAGTCCTAGGTATATACATACATGCTTGTTTGTTTGTAAACATAGCTTCTTACCGGA
CCTGTAGGATCGTACAGGTTTACGCAAGAAAATGGTTTGTTATTTCGAATAAAAGCGGTCAACGCATGTGCTTTGCGTTCTGATGAGACAGTGATGTCGAA
ACCGCCTCTACAAATAATTTTGTTTAAGGAGCTATGGACTATGTTTGAAAGGCTGAAATACTAGATGGCACGTACCCCGAGCCGTAGCAGCATTGGTAGCCT
GCGTAGTCCGCATACCCATAAAGCAATTCTGACCAGCACCATTGAAATCCTGAAAGAATGTGGTTATAGCGGTCTGAGCATTGAAAGCGTTGCACGTCGTGC
CGGTGCAAGCAAACCGACCATTTATCGTTGGTGGACCAATAAAGCAGCACTGATTGCCGAAGTGTATGAAAATGAAAGCGAACAGGTGCGTAAATTTCCGGA
TCTGGGTAGCTTTAAAGCCGATCTGGATTTTCTGCTGCGTAATCTGTGGAAAGTTTGGCGTGAAACCATTTGTGGTGAAGCATTTCGTTGTGTTATTGCAGA
AGCACAGCTGGACCCTGCAACCCTGACCCAGCTGAAAGATCAGTTTATGGAACGTCGTCGTGAGATGCCGAAAAAACTGGTTGAAAATGCCATTAGCAATGG
TGAACTGCCGAAAGATACCAATCGTGAACTGCTGCTGGATATGATTTTTGGTTTTTGTTGGTATCGCCTGCTGACCGAACAGCTGACCGTTGAACAGGATAT
TGAAGAATTTACCTTCCTGCTGATTAATGGTGTTTGTCCGGGTACACAGCGTTAAGGAAACACAGAAAAAAGCCCGCACCTGACAGTGCGGGCTTTTTTTTT
TCGACCAAAGGTACGTACTCCACCGTTGGCTTTTTTCCCTATCAGTGATAGAGATTGACATCCCTATCAGTGATAGAGATAATGAGCACAGCGCTCAACGGG
TGTGCTTCCCGTTCTGATGAGTCCGTGAGGACGAAAGCGCCTCTACAAATAATTTTGTTTAAGAGTCTATGGACTATGTTTTCACATATGAGATACCAGGAT
GGCACGTAAAACCGCAGCAGAAGCAGAAGAAACCCGTCAGCGTATTATTGATGCAGCACTGGAAGTTTTTGTTGCACAGGGTGTTAGTGATGCAACCCTGGA
TCAGATTGCACGTAAAGCCGGTGTTACCCGTGGTGCAGTTTATTGGCATTTTAATGGTAAACTGGAAGTTCTGCAGGCAGTTCTGGCAAGCCGTCAGCATCC
GCTGGAACTGGATTTTACACCGGATCTGGGTATTGAACGTAGCTGGGAAGCAGTTGTTGTTGCAATGCTGGATGCAGTTCATAGTCCGCAGAGCAAACAGTT

Segment C Circuit

TAGCGAAATTCTGATTTATCAGGGTCTGGATGAAAGCGGTCTGATTCATAATCGTATGGTTCAGGCAAGCGATCGTTTTCTGCAGTATATTCATCAGGTTCT
GCGTCATGCAGTTACCCAGGGTGAACTGCCGATTAATCTGGATCTGCAGACCAGCATTGGTGTTTTTAAAGGTCTGATTACCGGTCTGCTGTATGAAGGTCT
GCGTAGCAAAGATCAGCAGGCACAGATTATCAAAGTTGCACTGGGTAGCTTTTGGGCACTGCTGCGTGAACCGCCTCGTTTTCTGCTGTGTGAAGAAGCACA
GATTAAACAGGTGAAATCCTTCGAATAATTCAGCCAAAAACTTAAGACCGCCGGTCTTGTCCACTACCTTGCAGTAATGCGGTGGACAGGATCGGCGGTTT
TCTTTTCTCTTCTCAAAGGTCGACGTACGGTGGAATCTGATTCGTTACCAATTGACATGATACGAAACGTACCGTATCGTTAAGGTAGTAGTCACCGGCTGT
GCTTGCCGGTCTGATGAGCCTGTGAAGGCGAAACTACCTCTACAAATAATTTTGTTTAAACCCCCGAGATGAAATACATCCTGTTTGAGGTGTGCGAAATGG
GTAAAAGCCGTGAACAGACCATGGAAAATATTCTGAAAGCAGCCAAAAAGAAATTCGGCGAACGTGGTTATGAAGGCACCAGCATTCAAGAAATTACCAAAG
AAGCCAAAGTTAACGTTGCAATGGCCAGCTATTACTTTAATGGCAAAGAGAACCTGTACTACGAGGTGTTCAAAAAATACGGTCTGGCAAATGAACTGCCGA
ACTTTCTGGAAAAAAACCAGTTTAATCCGATTAATGCCCTGCGTGAATATCTGACCGTTTTTACCACCCACATTAAAGAAAATCCGGAAATTGGCACCCTGG
CCTATGAAGAAATTATCAAAGAAAGCGCACGCCTGGAAAAAATCAAACCGTATTTTATCGGCAGCTTCGAACAGCTGAAAGAAATTCTGCAAGAGGGTGAAA
AACAGGGTGTGTTTCACTTTTTTAGCATCAACCATACCATCCATTGGATTACCAGCATTGTTCTGTTTCCGAAATTCAAAAAATTCATCGATAGCCTGGGTC
CGAATGAAACCAATGATACCAATCATGAATGGATGCCGGAAGATCTGGTTAGCCGTATTATTAGCGCACTGACCGATAAACCGAACATTTAAGTTATGAGTC
AGGAAAAAAAGGCGACAGAGTAATCTGTCGCCTTTTTTCTTTGCTTGCTTTAATG

GGAGCCCTTTGTGCGTCCAAACGGACGCACGGCGCTCTAAAGCGGGTCGCGATCTTTCAGATTCGCTCCTCGCGCTTTCAGTCTTTGTTTTGGCGCATGTCG
TTATCGCAAAACCGCTGCACACTTTTGCGCGACATGCTCTGATCCCCCTCATCTGGGGGGGCCTATCTGAGGGAATTTCCGATCCGGCTCGCCTGAACCATT
CTGCTTTCCACGAACTTGAAAACGCTACCAGGAATCTGAACGATTCGTTACCAATTGCCATATTTAAAATTCTTGTTTAAAATGCTAGCAGCGGTCAACGCA
TGTGCTTTGCGTTCTGATGAGACAGTGATGTCGAAACCGCCTCTACAAATAATTTTGTTTAACTATGGACTATGTTTGAAAGGGAGAAATACTAGATGGCAC
GTACCCCGAGCCGTAGCAGCATTGGTAGCCTGCGTAGTCCGCATACCCATAAAGCAATTCTGACCAGCACCATTGAAATCCTGAAAGAATGTGGTTATAGCG
GTCTGAGCATTGAAAGCGTTGCACGTCGTGCCGGTGCAAGCAAACCGACCATTTATCGTTGGTGGACCAATAAAGCAGCACTGATTGCCGAAGTGTATGAAA
ATGAAAGCGAACAGGTGCGTAAATTTCCGGATCTGGGTAGCTTTAAAGCCGATCTGGATTTTCTGCTGCGTAATCTGTGGAAAGTTTGGCGTGAAACCATTT
GTGGTGAAGCATTTCGTTGTGTTATTGCAGAAGCACAGCTGGACCCTGCAACCCTGACCCAGCTGAAAGATCAGTTTATGGAACGTCGTCGTGAGATGCCGA
AAAAACTGGTTGAAAATGCCATTAGCAATGGTGAACTGCCGAAAGATACCAATCGTGAACTGCTGCTGGATATGATTTTTGGTTTTTGTTGGTATCGCCTGC
TGACCGAACAGCTGACCGTTGAACAGGATATTGAAGAATTTACCTTCCTGCTGATTAATGGTGTTTGTCCGGGTACACAGCGTTAAGGAAACACAGAAAAAA
GCCCGCACCTGACAGTGCGGGCTTTTTTTTTTCGACCAAAGGTACGTCGTGTAAGTAGCGTAACAAACAGACAATCTGGTCTGTTTGTATTATGGAAAATTT
TTCTGTATAATAGATTCAACAAACAGACAATCTGGTCTGTTTGTATTATATAGCTTCTTACCGGACCTGTAGGATCGTACAGGTTTACGCAAGAAAATGGTT
TGTTATTTTCGAATAAAAGGGTGTCTCAAGGTGCGTACCTTGACTGATGAGTCCGAAAGGACGAAACACCCCTCTACAAATAATTTTGTTTAAAATGTTCCC
TAATAATCAGCAAAGAGGTTACTAGATGGCAGGCGCAGTTGGTCGTCCGCGTCGTAGTGCACCGCGTCGTGCAGGTAAAAATCCGCGTGAAGAAATTCTGGA
TGCAAGCGCAGAACTGTTTACCCGTCAGGGTTTTGCAACCACCAGTACCCATCAGATTGCAGATGCAGTTGGTATTCGTCAGGCAAGCCTGTATTATCATTT
TCCGAGCAAAACCGAAATCTTTCTGACCCTGCTGAAAAGCACCGTTGAACCGAGCACCGTTCTGGCAGAAGATCTGAGCACCCTGGATGCAGGTCCGGAAAT
GCGTCTGTGGGCAATTGTTGCAAGCGAAGTTCGTCTGCTGCTGAGCACCAAATGGAATGTTGGTCGTCTGTATCAGCTGCCGATTGTTGGTAGCGAAGAATT
TGCAGAATATCATAGCCAGCGTGAAGCACTGACCAATGTTTTTCGTGATCTGGCAACCGAAATTGTTGGTGATGATCCGCGTGCAGAACTGCCGTTTCATAT
TACCATGAGCGTTATTGAAATGCGTCGCAATGATGGTAAAATTCCGAGTCCGCTGAGCGCAGATAGCCTGCCGGAAACCGCAATTATGCTGGCAGATGCAAG
CCTGGCAGTTCTGGGTGCACCGCTGCCTGCAGATCGTGTTGAAAAAACCCTGGAACTGATTAAACAGGCAGATGCAAAATAACTCGGTACCAAAGACGAACA
ATAAGACGCTGAAAAGCGTCTTTTTTCGTTTTGGTCCAGGTCCCTTTGTGCGTCCAAACGGACGCACGGCGCTCTAAAGCGGGTCGCGATCTTTCAGATTCG
CTCCTCGCGCTTTCAGTCTTTGTTTTGGCGCATGTCGTTATCGCAAAACCGCTGCACACTTTTGCGCGACATGCTCTGATCCCCCTCATCTGGGGGGGCCTA
TCTGAGGGAATTTCCGATCCGGCTCGCCTGAACCATTCTGCTTTCCACGAACTTGAAAACGCTAGGGGTCAGTTGATGTGCTTTCAACTCTGATGAGTCAGT
GATGACGAAACCCCCTCTACAAATAATTTTGTTTAATACGCTATGGACTATGTTTCAAACAGGAGCTAATAGATGAACAAAACCATTGATCAGGTGCGTAAA
GGTGATCGTAAAAGCGATCTGCCGGTTCGTCGTCGTCCGCGTCGTAGTGCCGAAGAAACCCGTCGTGATATTCTGGCAAAAGCCGAAGAACTGTTTCGTGAA

Segment D Circuit

CGTGGTTTTAATGCAGTTGCCATTGCAGATATTGCAAGCGCACTGAATATGAGTCCGGCAAATGTGTTTAAACATTTTAGCAGCAAAAACGCACTGGTTGAT
GCAATTGGTTTTGGTCAGATTGGTGTTTTTGAACGTCAGATTTGTCCGCTGGATAAAAGCCATGCACCGCTGGATCGTCTGCGTCATCTGGCACGTAATCTG
ATGGAACAGCATCATCAGGATCATTTCAAACACATACGGGTTTTATTCAGATCCTGATGACCGCCAAACAGGATATGAAATGTGGCGATTATTACAAAAGC
GTGATTGCAAAACTGCTGGCCGAAATTATTCGTGATGGTGTTGAAGCAGGTCTGTATATTGCAACCGATATTCCGGTTCTGGCAGAAACCGTTCTGCATGCA
CTGACCAGCGTTATTCATCCGGTTCTGATTGCACAAGAAGATATTGGTAATCTGGCAACCCGTTGTGATCAGCTGGTTGATCTGATTGATGCAGGTCTGCGT
AATCCGCTGGCAAAATAACCAATTATTGAACACCCTAACGGGTGTTTTTTTTTTTTTGGTCTCCCGCTTTCGTCACTAGAGGGCGATAGTGACAAACTTGAC
AACTCATCACTTCCTACGTAGGCTGCTAGCCGCTCATTCACTAGGTCTGATTCGTTACCAATTGACAGCTGGTGGTCGAATCAAGATAATAGACCAGTCACT
ATATTTAGACTGTCGCCGGATGTGTATCCGACCTGACGATGGCCCAAAAGGGCCGAAACAGTCCTCTACAAATAATTTTGTTTAACCAAACGAGGCCGGGAG
GATGGAAAGCACCCCGACCAAACAGAAAGCAATTTTTAGCGCAAGCCTGCTGCTGTTTGCAGAACGTGGTTTTGATGCAACCACCATGCCGATGATTGCAGA
AAATGCAAAAGTTGGTGCAGGCACCATTTATCGCTATTTCAAAAACAAAGAAAGCCTGGTGAACGAACTGTTTCAGCAGCATGTTAATGAATTTCTGCAGTG
TATTGAAAGCGGTCTGGCAAATGAACGTGATGGTTATCGTGATGGCTTTCATCACATTTTTGAAGGTATGGTGACCTTTACCAAAAATCATCCGCGTGCACT
GGGTTTTATCAAAACCCATAGCCAGGGCACCTTTCTGACCGAAGAAAGCCGTCTGGCATATCAGAAACTGGTTGAATTTGTGTGCACCTTTTTTCGTGAAGG
TCAGAAACAGGGTGTGATTCGTAATCTGCCGGAAAATGCACTGATTGCAATTCTGTTTGGCAGCTTTATGGAAGTGTATGAAATGATCGAGAACGATTATCT
GAGCCTGACCGATGAACTGCTGACCGGTGTTGAAGAAAGCCTGTGGGCAGCACTGAGCCGTCAGAGCTAACTCGGTACCAAATTCCAGAAAAGAGACGCTTT
CGAGCGTCTTTTTTCGTTTTGGTCCCGCTACCAGGAATCTGAACGATTCGTTACCAATTGCCATATTTAAAATTCTTGTTTAAAATGCTAGCCTTGTCCAAC
CAAATGATTCGTTACCCTTTGACAGTTTCTATCGATCTATAGATAATGCTAGCAGGAGTCAATTAATGTGCTTTTAATTCTGATGAGACGGTGACGTCGAAA
CTCCCTCTACAAATAATTTTGTTTAATACGCTATGGACTATGTTTTCTGCTATGGACTATGTTTTCACACACGAGATGCCTCGATGAGCTATGGTGATAGCC
GTGAAAAAATTCTGAGCGCAGCAACCCGTCTGTTTCAGCTGCAGGGTTATTATGGCACCGGTCTGAATCAGATTATCAAAGAAAGCGGTGCACCGAAAGGTA
GCCTGTATTATCATTTTCCGGGTGGTAAAGAACAGCTGGCAATTGAAGCAGTGAACGAAATGAAAGAATATATCCGCCAGAAAATCGCCGATTGTATGGAAG
CATGTACCGATCCGGCAGAAGGTATTCAGGCATTTCTGAAAGAACTGAGCTGTCAGTTTAGCTGTACCGAAGATATTGAAGGTCTGCCGGTTGGTCTGCTGG
CAGCAGAAACCAGCCTGAAAAGCGAACCGCTGCGTGAAGCATGTCATGAAGCATATAAAGAATGGGCCAGCGTGTATGAAGAAAAACTGCGTCAGACCGGTT
GTAGCGAAAGCCGTGCAAAAGAAGCAAGCACCGTTGTTAATGCAATGATTGAAGGTGGTATTCTGCTGAGCCTGACCGCAAAAAATAGCACACCGCTGCTGC
ATATTAGCAGCTGTATTCCGGATCTGCTGAAACGTTAATAAGGTTGAAAAATAAAAACGGCGCTAAAAAGCGCCGTTTTTTTTGACGGTGGTATGTCTCGTG
TAAGTAGCGTAACAAACAGACAATCTGGTCTGTTTGTATTATGGAAAATTTTTCTGTATAATAGATTCAACAAACAGACAATCTGGTCTGTTTGTATTATTA
CTCCACCGTTGGCTTTTTTCCCTATCAGTGATAGAGATTGACATCCCTATCAGTGATAGAGATAATGAGCACAGTAGTCACCGGCTGTGCTTGCCGGTCTGA

TGAGCCTGTGAAGGCGAAACTACCTCTACAAATAATTTTGTTTAAACCCCCGAGATGAAATACATCCTGTTTGAGGTGTGCGAAATGGGTAAAAGCCGTGAA
CAGACCATGGAAAATATTCTGAAAGCAGCCAAAAAGAAATTCGGCGAACGTGGTTATGAAGGCACCAGCATTCAAGAAATTACCAAAGAAGCCAAAGTTAAC
GTTGCAATGGCCAGCTATTACTTTAATGGCAAAGAGAACCTGTACTACGAGGTGTTCAAAAAATACGGTCTGGCAAATGAACTGCCGAACTTTCTGGAAAAA
AACCAGTTTAATCCGATTAATGCCCTGCGTGAATATCTGACCGTTTTACCACCCACATTAAAGAAAATCCGGAAATTGGCACCCTGGCCTATGAAGAAATT
ATCAAAGAAAGCGCACGCCTGGAAAAAATCAAACCGTATTTTATCGGCAGCTTCGAACAGCTGAAAGAAATTCTGCAAGAGGGTGAAAAACAGGGTGTGTTT
CACTTTTTTAGCATCAACCATACCATCCATTGGATTACCAGCATTGTTCTGTTTCCGAAATTCAAAAAATTCATCGATAGCCTGGGTCCGAATGAAACCAAT
GATACCAATCATGAATGGATGCCGGAAGATCTGGTTAGCCGTATTATTAGCGCACTGACCGATAAACCGAACATTTAAGTTATGAGTCAGGAAAAAAGGCGA
CAGAGTAATCTGTCGCCTTTTTTCTTTGCTTGCTTTTCTGATAGCTTCTTACCGGACCTGTAGGATCGTACAGGTTTACGCAAGAAAATGGTTTGTTATTTT
CGAATAAATACTCCACCGTTGGCTTTTTTCCCTATCAGTGATAGAGATTGACATCCCTATCAGTGATAGAGATAATGAGCACAGTACGTCTGAGCGTGATAC
CCGCTCACTGAAGATGGCCCGGTAGGGCCGAAACGTACCTCTACAAATAATTTTGTTTAAGACTTAAACGATTGGCCGACCGCATGAGCCCGAAACGTCGTA
CCCAGGCAGAACGTGCAATGGAAACCCAGGGTAAACTGATTGCAGCAGCACTGGGTGTTCTGCGTGAAAAAGGTTATGCAGGTTTTCGTATTGCAGATGTTC
CGGGTGCAGCCGGTGTTAGCCGTGGTGCACAGAGCCATCATTTTCCGACCAAACTGGAACTGCTGCTGGCAACCTTTGAATGGCTGTATGAGCAGATTACCG
AACGTAGCCGTGCACGTCTGGCAAAACTGAAACCGGAAGATGATGTTATTCAGCAGATGCTGGATGATGCAGCAGAATTTTTTCTGGATGATGATTTTAGCA
TCAGCCTGGATCTGATTGTTGCAGCAGATCGTGATCCGGCACTGCGTGAAGGTATTCAGCGTACCGTTGAACGTAATCGTTTTGTTGTTGAAGATATGTGGC
TGGGTGTGCTGGTGAGCCGTGGTCTGAGCCGTGATGATGCCGAAGATATTCTGTGGCTGATTTTTAACAGCGTTCGTGGTCTGGCAGTTCGTAGCCTGTGGC
AGAAAGATAAAGAACGTTTTGAACGTGTGCGTAATAGCACCCTGGAAATTGCACGTGAACGTTATGCAAAATTCAAACGTTGATAACTCGGTACCAAAGAAA
AATAAAAAGACGCTGAAAAGCGTCTTTTTATTTTTCGGTCCAATG

Segment E   Circuit

GGAGTCTATGATTGGTCCAGATTCGTTACCAATTGACAGCTAGCTCAGTCCTAGGTATATACATACATGCTTGTTTGTTTGTAAACATAGCTTCTTACCGGA
CCTGTAGGATCGTACAGGTTTACGCAAGAAAATGGTTTGTTATTTTCGAATAAAAGCGGTCAACGCATGTGCTTTGCGTTCTGATGAGACAGTGATGTCGAA
ACCGCCTCTACAAATAATTTTGTTTAACTTTACGAGGGCGATCCTATGGCACGTACCCCGAGCCGTAGCAGCATTGGTAGCCTGCGTAGTCCGCATACCCAT
AAAGCAATTCTGACCAGCACCATTGAAATCCTGAAAGAATGTGGTTATAGCGGTCTGAGCATTGAAAGCGTTGCACGTCGTGCCGGTGCAAGCAAACCGACC
ATTTATCGTTGGTGGACCAATAAAGCAGCACTGATTGCCGAAGTGTATGAAAATGAAAGCGAACAGGTGCGTAAATTTCCGGATCTGGGTAGCTTTAAAGCC
GATCTGGATTTTCTGCTGCGTAATCTGTGGAAAGTTTGGCGTGAAACCATTTGTGGTGAAGCATTTCGTTGTGTTATTGCAGAAGCACAGCTGGACCCTGCA
ACCCTGACCCAGCTGAAAGATCAGTTTATGGAACGTCGTCGTGAGATGCCGAAAAAACTGGTTGAAAATGCCATTAGCAATGGTGAACTGCCGAAAGATACC
AATCGTGAACTGCTGCTGGATATGATTTTTGGTTTTTGTTGGTATCGCCTGCTGACCGAACAGCTGACCGTTGAACAGGATATTGAAGAATTTACCTTCCTG
CTGATTAATGGTGTTTGTCCGGGTACACAGCGTTAAGGAAACACAGAAAAAAGCCCGCACCTGACAGTGCGGGCTTTTTTTTTCGACCAAAGGTACGACCA
GGAATCTGAACGATTCGTTACCAATTGCCATATTTAAAATTCTTGTTTAAAATGCTAGCTACTCCACCGTTGGCTTTTTTCCCTATCAGTGATAGAGATTGA
CATCCCTATCAGTGATAGAGATAATGAGCACAGCGCTCAACGGGTGTGCTTCCCGTTCTGATGAGTCCGTGAGGACGAAAGCGCCTCTACAAATAATTTTGT
TTAACTATGGACTATGTTTTCACACAGGAAATACCAGGATGGCACGTAAAACCGCAGCAGAAGCAGAAGAAACCCGTCAGCGTATTATTGATGCAGCACTGG
AAGTTTTTGTTGCACAGGGTGTTAGTGATGCAACCCTGGATCAGATTGCACGTAAAGCCGGTGTTACCCGTGGTGCAGTTTATTGGCATTTTAATGGTAAAC
TGGAAGTTCTGCAGGCAGTTCTGGCAAGCCGTCAGCATCCGCTGGAACTGGATTTTACACCGGATCTGGGTATTGAACGTAGCTGGGAAGCAGTTGTTGTTG
CAATGCTGGATGCAGTTCATAGTCCGCAGAGCAAACAGTTTAGCGAAATTCTGATTTATCAGGGTCTGGATGAAAGCGGTCTGATTCATAATCGTATGGTTC
AGGCAAGCGATCGTTTTCTGCAGTATATTCATCAGGTTCTGCGTCATGCAGTTACCCAGGGTGAACTGCCGATTAATCTGGATCTGCAGACCAGCATTGGTG
TTTTTAAAGGTCTGATTACCGGTCTGCTGTATGAAGGTCTGCGTAGCAAAGATCAGCAGGCACAGATTATCAAAGTTGCACTGGGTAGCTTTTGGGCACTGC
TGCGTGAACCGCCTCGTTTTCTGCTGTGTGAAGAAGCACAGATTAAACAGGTGAAATCCTTCGAATAATTCAGCCAAAAAACTTAAGACCGCCGGTCTTGTC
CACTACCTTGCAGTAATGCGGTGGACAGGATCGGCGGTTTTCTTTTCTCTTCTCAAAGGTCCCTTTGTGCGTCCAAACGGACGCACGGCGCTCTAAAGCGGG
TCGCGATCTTTCAGATTCGCTCCTCGCGCTTTCAGTCTTTGTTTTGGCGCATGTCGTTATCGCAAAACCGCTGCACACTTTTGCGCGACATGCTCTGATCCC
CCTCATCTGGGGGGGCCTATCTGAGGGAATTTCCGATCCGGCTCGCCTGAACCATTCTGCTTTCCACGAACTTGAAAACGCTAGTAGTCACCGGCTGTGCTT
GCCGGTCTGATGAGCCTGTGAAGGCGAAACTACCTCTACAAATAATTTTGTTTAAACCCCCGAGATGAAATACATCCTGTTTGAGGTGTGCGAAATGGGTAA
AAGCCGTGAACAGACCATGGAAAATATTCTGAAAGCAGCCAAAAAGAAATTCGGCGAACGTGGTTATGAAGGCACCAGCATTCAAGAAATTACCAAAGAAGC
CAAAGTTAACGTTGCAATGGCCAGCTATTACTTTAATGGCAAAGAGAACCTGTACTACGAGGTGTTCAAAAAATACGGTCTGGCAAATGAACTGCCGAACTT
TCTGGAAAAAAACCAGTTTAATCCGATTAATGCCCTGCGTGAATATCTGACCGTTTTACCACCCACATTAAAGAAAATCCGGAAATTGGCACCCTGGCCTA
TGAAGAAATTATCAAAGAAAGCGCACGCCTGGAAAAAATCAAACCGTATTTTATCGGCAGCTTCGAACAGCTGAAAGAAATTCTGCAAGAGGGTGAAAAACA
GGGTGTGTTTCACTTTTTTAGCATCAACCATACCATCCATTGGATTACCAGCATTGTTCTGTTTCCGAAATTCAAAAAATTCATCGATAGCCTGGGTCCGAA
TGAAACCAATGATACCAATCATGAATGGATGCCGGAAGATCTGGTTAGCCGTATTATTAGCGCACTGACCGATAAACCGAACATTTAAGTTATGAGTCAGGA
AAAAAGGCGACAGAGTAATCTGTCGCCTTTTTTCTTTGCTTGCTTTAATG

Segment F   Circuit

GGAGAATCCGCGTGATAGGTCTGATTCGTTACCAATTGACGGAATGAACGTTCATTCCGATAATGCTAGCATAGCTTCTTACCGGACCTGTAGGATCGTACA
GGTTTACGCAAGAAAATGGTTTGTTATTTTCGAATAAAAGCGGTCAACGCATGTGCTTTGCGTTCTGATGAGACAGTGATGTCGAAACCGCCTCTACAAATA
ATTTTGTTTAACTATGGACTATGTTTGAAAGGGAGAAATACTAGATGGCACGTACCCCGAGCCGTAGCAGCATTGGTAGCCTGCGTAGTCCGCATACCCATA
AAGCAATTCTGACCAGCACCATTGAAATCCTGAAAGAATGTGGTTATAGCGGTCTGAGCATTGAAAGCGTTGCACGTCGTGCCGGTGCAAGCAAACCGACCA
TTTATCGTTGGTGGACCAATAAAGCAGCACTGATTGCCGAAGTGTATGAAAATGAAAGCGAACAGGTGCGTAAATTTCCGGATCTGGGTAGCTTTAAAGCCG
ATCTGGATTTTCTGCTGCGTAATCTGTGGAAAGTTTGGCGTGAAACCATTTGTGGTGAAGCATTTCGTTGTGTTATTGCAGAAGCACAGCTGGACCCTGCAA
CCCTGACCCAGCTGAAAGATCAGTTTATGGAACGTCGTCGTGAGATGCCGAAAAAACTGGTTGAAAATGCCATTAGCAATGGTGAACTGCCGAAAGATACCA
ATCGTGAACTGCTGCTGGATATGATTTTTGGTTTTTGTTGGTATCGCCTGCTGACCGAACAGCTGACCGTTGAACAGGATATTGAAGAATTTACCTTCCTGC
TGATTAATGGTGTTTGTCCGGGTACACAGCGTTAAGGAAACACAGAAAAAAGCCCGCACCTGACAGTGCGGGCTTTTTTTTTCGACCAAAGGTACGCCCTT
TGTGCGTCCAAACGGACGCACGGCGCTCTAAAGCGGGTCGCGATCTTTCAGATTCGCTCCTCGCGCTTTCAGTCTTTGTTTTGGCGCATGTCGTTATCGCAA
AACCGCTGCACACTTTTGCGCGACATGCTCTGATCCCCCTCATCTGGGGGGGCCTATCTGAGGGAATTTCCGATCCGGCTCGCCTGAACCATTCTGCTTTCC
ACGAACTTGAAAACGCTAGGGTGTCTCAAGGTGCGTACCTTGACTGATGAGTCCGAAAGGACGAAACACCCCTCTACAAATAATTTTGTTTAAAATGTTCCC
TAATAATCAGCAAAGAGGTTACTAGATGGCAGGCGCAGTTGGTCGTCCGCGTCGTAGTGCACCGCGTCGTGCAGGTAAAAATCCGCGTGAAGAAATTCTGGA
TGCAAGCGCAGAACTGTTTACCCGTCAGGGTTTTGCAACCACCAGTACCCATCAGATTGCAGATGCAGTTGGTATTCGTCAGGCAAGCCTGTATTATCATTT
TCCGAGCAAAACCGAAATCTTTCTGACCCTGCTGAAAAGCACCGTTGAACCGAGCACCGTTCTGGCAGAAGATCTGAGCACCCTGGATGCAGGTCCGGAAAT
GCGTCTGTGGGCAATTGTTGCAAGCGAAGTTCGTCTGCTGCTGAGCACCAAATGGAATGTTGGTCGTCTGTATCAGCTGCCGATTGTTGGTAGCGAAGAATT
TGCAGAATATCATAGCCAGCGTGAAGCACTGACCAATGTTTTTCGTGATCTGGCAACCGAAATTGTTGGTGATGATCCGCGTGCAGAACTGCCGTTTCATAT
TACCATGAGCGTTATTGAAATGCGTCGCAATGATGGTAAAATTCCGAGTCCGCTGAGCGCAGATAGCCTGCCGGAAACCGCAATTATGCTGGCAGATGCAAG
CCTGGCAGTTCTGGGTGCACCGCTGCCTGCAGATCGTGTTGAAAAAACCCTGGAACTGATTAAACAGGCAGATGCAAAATAACTCGGTACCAAAGACGAACA
ATAAGACGCTGAAAAGCGTCTTTTTTCGTTTTGGTCCAGGTCCCTTTGTGCGTCCAAACGGACGCACGGCGCTCTAAAGCGGGTCGCGATCTTTCAGATTCG
CTCCTCGCGCTTTCAGTCTTTGTTTTGGCGCATGTCGTTATCGCAAAACCGCTGCACACTTTTGCGCGACATGCTCTGATCCCCCTCATCTGGGGGGGCCTA
TCTGAGGGAATTTCCGATCCGGCTCGCCTGAACCATTCTGCTTTCCACGAACTTGAAAACGCTAGCGCGGGTGAGAGGGATTCGTTACCAATAGACAATTGA
TTGGACGTTCAATATAATGCTAGCAGACTGTCGCCGGATGTGTATCCGACCTGACGATGGCCCAAAAGGGCCGAAACAGTCCTCTACAAATAATTTTGTTTA
ACCAAACGAGGCCGGGAGGATGGAAAGCACCCCGACCAAACAGAAAGCAATTTTTAGCGCAAGCCTGCTGCTGTTTGCAGAACGTGGTTTTGATGCAACCAC
CATGCCGATGATTGCAGAAAATGCAAAAGTTGGTGCAGGCACCATTTATCGCTATTTCAAAAACAAAGAAAGCCTGGTGAACGAACTGTTTCAGCAGCATGT
TAATGAATTTCTGCAGTGTATTGAAAGCGGTCTGGCAAATGAACGTGATGGTTATCGTGATGGCTTTCATCACATTTTTGAAGGTATGGTGACCTTTACCAA
AAATCATCCGCGTGCACTGGGTTTATCAAAACCCATAGCCAGGGCACCTTTCTGACCGAAGAAAGCCGTCTGGCATATCAGAAACTGGTTGAATTTGTGTG
CACCTTTTTTCGTGAAGGTCAGAAACAGGGTGTGATTCGTAATCTGCCGGAAAATGCACTGATTGCAATTCTGTTTGGCAGCTTTATGGAAGTGTATGAAAT
GATCGAGAACGATTATCTGAGCCTGACCGATGAACTGCTGACCGGTGTTGAAGAAAGCCTGTGGGCAGCACTGAGCCGTCAGAGCTAACTCGGTACCAAATT
CCAGAAAAGAGACGCTTTCGAGCGTCTTTTTTCGTTTTGGTCCGCTTCTTGTCCAACCAAATGATTCGTTACCCTTTGACAGTTTCTATCGATCTATAGATA
ATGCTAGCTACTCCACCGTTGGCTTTTTTCCCTATCAGTGATAGAGATTGACATCCCTATCAGTGATAGAGATAATGAGCACAGGAGTCAATTAATGTGCTT
TTAATTCTGATGAGACGGTGACGTCGAAACTCCCTCTACAAATAATTTTGTTTAATACGCTATGGACTATGTTTTCTGCTATGGACTATGTTTTCACACACG
AGATGCCTCGATGAGCTATGGTGATAGCCGTGAAAAAATTCTGAGCGCAGCAACCCGTCTGTTTCAGCTGCAGGGTTATTATGGCACCGGTCTGAATCAGAT
TATCAAAGAAAGCGGTGCACCGAAAGGTAGCCTGTATTATCATTTTCCGGGTGGTAAAGAACAGCTGGCAATTGAAGCAGTGAACGAAATGAAAGAATATAT
CCGCCAGAAAATCGCCGATTGTATGGAAGCATGTACCGATCCGGCAGAAGGTATTCAGGCATTTCTGAAAGAACTGAGCTGTCAGTTTAGCTGTACCGAAGA
TATTGAAGGTCTGCCGGTTGGTCTGCTGGCAGCAGAAACCAGCCTGAAAAGCGAACCGCTGCGTGAAGCATGTCATGAAGCATATAAAGAATGGGCCAGCGT
GTATGAAGAAAAACTGCGTCAGACCGGTTGTAGCGAAAGCCGTGCAAAAGAAGCAAGCACCGTTGTTAATGCAATGATTGAAGGTGGTATTCTGCTGAGCCT
GACCGCAAAAAATAGCACACCGCTGCTGCATATTAGCAGCTGTATTCCGGATCTGCTGAAACGTTAATAAGGTTGAAAAATAAAAACGGCGCTAAAAAGCGC
CGTTTTTTTTGACGGTGGTACGCTTCGTGTAAGTAGCGTAACAAACAGACAATCTGGTCTGTTTGTATTATGGAAAATTTTTCTGTATAATAGATTCAACAA
ACAGACAATCTGGTCTGTTTGTATTATAGTAGTCACCGGCTGTGCTTGCCGGTCTGATGAGCCTGTGAAGGCGAAACTACCTCTACAAATAATTTTGTTTAA
ACCCCCGAGATGAAATACATCCTGTTTGAGGTGTGCGAAATGGGTAAAAGCCGTGAACAGACCATGGAAAATATTCTGAAAGCAGCCAAAAAGAAATTCGGC
GAACGTGGTTATGAAGGCACCAGCATTCAAGAAATTACCAAAGAAGCCAAAGTTAACGTTGCAATGGCCAGCTATTACTTTAATGGCAAAGAGAACCTGTAC
TACGAGGTGTTCAAAAAATACGGTCTGGCAAATGAACTGCCGAACTTTCTGGAAAAAAACCAGTTTAATCCGATTAATGCCCTGCGTGAATATCTGACCGTT
TTTACCACCCACATTAAAGAAAATCCGGAAATTGGCACCCTGGCCTATGAAGAAATTATCAAAGAAAGCGCACGCCTGGAAAAAATCAAACCGTATTTTATC
GGCAGCTTCGAACAGCTGAAAGAAATTCTGCAAGAGGGTGAAAAACAGGGTGTGTTTCACTTTTTTAGCATCAACCATACCATCCATTGGATTACCAGCATT
GTTCTGTTTCCGAAATTCAAAAAATTCATCGATAGCCTGGGTCCGAATGAAACCAATGATACCAATCATGAATGGATGCCGGAAGATCTGGTTAGCCGTATT

Segment G Circuit

ATTAGCGCACTGACCGATAAACCGAACATTTAAGTTATGAGTCAGGAAAAAAGGCGACAGAGTAATCTGTCGCCTTTTTTCTTTGCTTGCTTTTGTCTACTC
CACCGTTGGCTTTTTTCCCTATCAGTGATAGAGATTGACATCCCTATCAGTGATAGAGATAATGAGCACAGAAGTCAATTAATGTGCTTTTAATTCTGATGA
GTCGGTGACGACGAAACTTCCTCTACAAATAATTTTGTTTAACCCCCCGAGGAGTAGCACATGCCGAAACTGGGTATGCAGAGCATTCGTCGTCGTCAGCTG
ATTGATGCAACCCTGGAAGCAATTAATGAAGTTGGTATGCATGATGCAACCATTGCACAGATTGCACGTCGTGCCGGTGTTAGCACCGGTATTATTAGCCAT
TATTTCCGCGATAAAAACGGTCTGCTGGAAGCAACCATGCGTGATATTACCAGCCAGCTGCGTGATGCAGTTCTGAATCGTCTGCATGCACTGCCGCAGGGT
AGCGCAGAACAGCGTCTGCAGGCAATTGTTGGTGGTAATTTTGATGAAACCCAGGTTAGCAGCGCAGCAATGAAAGCATGGCTGGCATTTTGGGCAAGCAGC
ATGCATCAGCCGATGCTGTATCGTCTGCAGCAGGTTAGCAGTCGTCGTCTGCTGAGCAATCTGGTTAGCGAATTTCGTCGTGAACTGCCTCGTGAACAGGCA
CAAGAGGCAGGTTATGGTCTGGCAGCACTGATTGATGGTCTGTGGCTGCGTGCAGCACTGAGCGGTAAACCGCTGGATAAAACCCGTGCAAATAGCCTGACC
CGTCATTTTATCACCCAGCATCTGCCGACCGATTAACCAATTATTGAACACCCTTCGGGGTGTTTTTTTGTTTCTGGTCTCCCTCTGAACGATCGTTGGCTG
TGTTGACAATTAATCATCGGCTCGTATAATGTGTGGAATTGTGAGCGCTCACAATTCGCTCATTCACTAGGTCTGATTCGTTACCAATTGACAGCTGGTGGT
CGAATCAAGATAATAGACCAGTCACTATATTTAGTACGTCTGAGCGTGATACCCGCTCACTGAAGATGGCCCGGTAGGGCCGAAACGTACCTCTACAAATAA
TTTTGTTTAAGACTTAAACGATTGGCCGACCGCATGAGCCCGAAACGTCGTACCCAGGCAGAACGTGCAATGGAAACCCAGGGTAAACTGATTGCAGCAGCA
CTGGGTGTTCTGCGTGAAAAAGGTTATGCAGGTTTTCGTATTGCAGATGTTCCGGGTGCAGCCGGTGTTAGCCGTGGTGCACAGAGCCATCATTTTCCGACC
AAACTGGAACTGCTGCTGGCAACCTTTGAATGGCTGTATGAGCAGATTACCGAACGTAGCCGTGCACGTCTGGCAAAACTGAAACCGGAAGATGATGTTATT
CAGCAGATGCTGGATGATGCAGCAGAATTTTTTCTGGATGATGATTTTAGCATCAGCCTGGATCTGATTGTTGCAGCAGATCGTGATCCGGCACTGCGTGAA
GGTATTCAGCGTACCGTTGAACGTAATCGTTTTGTTGTTGAAGATATGTGGCTGGGTGTGCTGGTGAGCCGTGGTCTGAGCCGTGATGATGCCGAAGATATT
CTGTGGCTGATTTTTAACAGCGTTCGTGGTCTGGCAGTTCGTAGCCTGTGGCAGAAAGATAAAGAACGTTTTGAACGTGTGCGTAATAGCACCCTGGAAATT
GCACGTGAACGTTATGCAAAATTCAAACGTTGATAACTCGGTACCAAAGAAAAATAAAAAGACGCTGAAAAGCGTCTTTTTATTTTTCGGTCCAATG

GGAGTACTCCACCGTTGGCTTTTTTCCCTATCAGTGATAGAGATTGACATCCCTATCAGTGATAGAGATAATGAGCACAGTGGTCGTGATCTGAAACTCGAT
CACCTGATGAGCTCAAGGCAGAGCGAAACCACCTCTACAAATAATTTTGTTTAAAAGACCATTATAAGGTTTGAACTATGGACATGCCTCGTATTAAACCGG
GTCAGCGTGTTATGATGGCACTGCGTAAAATGATTGCAAGCGGTGAAATCAAAAGTGGTGAACGTATTGCAGAAATTCCGACCGCAGCAGCACTGGGTGTTA
GCCGTATGCCGGTTCGTATCGCACTGCGTTCACTGGAACAAGAAGGTCTGGTTGTTCGTCTGGGTGCACGTGGTTATGCAGCCCGTGGTGTTAGCAGCGATC
AGATTCGTGATGCAATTGAAGTTCGTGGTGTTCTGGAAGGTTTTGCAGCACGTCGTCTGGCAGAACGTGGTATGACCGCAGAAACCCATGCACGTTTTGTTG
TACTGATTGCAGAAGGTGAAGCACTGTTTGCAGCCGGTCGCCTGAATGGTGAAGATCTGGATCGTTATGCCGCATATAATCAGGCATTTCATGATACCCTGG
TTAGCGCAGCAGGTAATGGTGCAGTTGAAAGCGCACTGGCACGTAATGGTTTTGAACCGTTTGCAGCAGCCGGTGCACTGGCCCTGGATCTGATGGACCTGT
CTGCCGAATATGAACATCTGCTGGCAGCACATCGTCAGCATCAGGCAGTTCTGGATGCAGTTAGCTGTGGTGATGCCGAAGGTGCAGAACGTATTATGCGTG
ATCATGCACTGGCAGCAATTCGTAATGCAAAAGTTTTTGAAGCAGCAGCAAGCGCAGGCGCACCGCTGGGTGCAGCATGGTCAATTCGTGCAGATTGATAAA
ACGCATGAGAAAGCCCCCGGAAGATCACCTTCCGGGGGCTTTTTTATTGCGCTACGTCGTGTAAGTAGCGTAACAAACAGACAATCTGGTCTGTTTGTATTA
TGGAAAATTTTTCTGTATAATAGATTCAACAAACAGACAATCTGGTCTGTTTGTATTATAGCGGTCAACGCATGTGCTTTGCGTTCTGATGAGACAGTGATG
TCGAAACCGCCTCTACAAATAATTTTGTTTAACTATGGACTATGTTTGAAAGGGAGAAATACTAGATGGCACGTACCCCGAGCCGTAGCAGCATTGGTAGCC
TGCGTAGTCCGCATACCCATAAAGCAATTCTGACCAGCACCATTGAAATCCTGAAAGAATGTGGTTATAGCGGTCTGAGCATTGAAAGCGTTGCACGTCGTG
CCGGTGCAAGCAAACCGACCATTTATCGTTGGTGGACCAATAAAGCAGCACTGATTGCCGAAGTGTATGAAAATGAAAGCGAACAGGTGCGTAAATTTCCGG
ATCTGGGTAGCTTTAAAGCCGATCTGGATTTTCTGCTGCGTAATCTGTGGAAAGTTTGGCGTGAAACCATTTGTGGTGAAGCATTTCGTTGTGTTATTGCAG
AAGCACAGCTGGACCCTGCAACCCTGACCCAGCTGAAAGATCAGTTTATGGAACGTCGTCGTGAGATGCCGAAAAAACTGGTTGAAAATGCCATTAGCAATG
GTGAACTGCCGAAAGATACCAATCGTGAACTGCTGCTGGATATGATTTTTGGTTTTTGTTGGTATCGCCTGCTGACCGAACAGCTGACCGTTGAACAGGATA
TTGAAGAATTTACCTTCCTGCTGATTAATGGTGTTTGTCCGGGTACACAGCGTTAAGGAAACACAGAAAAAAGCCCGCACCTGACAGTGCGGGCTTTTTTTT
TTCGACCAAAGGAGGTATAGCTTCTTACCGGACCTGTAGGATCGTACAGGTTTACGCAAGAAAATGGTTTGTTATTTTCGAATAAAAGGGTGTCTCAAGGTG
CGTACCTTGACTGATGAGTCCGAAAGGACGAAACACCCCTCTACAAATAATTTTGTTTAAAATGTTCCCTAATAATCAGCAAAGAGGTTACTAGATGGCAGG
CGCAGTTGGTCGTCCGCGTCGTAGTGCACCGCGTCGTGCAGGTAAAAATCCGCGTGAAGAAATTCTGGATGCAAGCGCAGAACTGTTTACCCGTCAGGGTTT
TGCAACCACCAGTACCCATCAGATTGCAGATGCAGTTGGTATTCGTCAGGCAAGCCTGTATTATCATTTTCCGAGCAAAACCGAAATCTTTCTGACCCTGCT
GAAAAGCACCGTTGAACCGAGCACCGTTCTGGCAGAAGATCTGAGCACCCTGGATGCAGGTCCGGAAATGCGTCTGTGGGCAATTGTTGCAAGCGAAGTTCG
TCTGCTGCTGAGCACCAAATGGAATGTTGGTCGTCTGTATCAGCTGCCGATTGTTGGTAGCGAAGAATTTGCAGAATATCATAGCCAGCGTGAAGCACTGAC
CAATGTTTTTCGTGATCTGGCAACCGAAATTGTTGGTGATGATCCGCGTGCAGAACTGCCGTTTCATATTACCATGAGCGTTATTGAAATGCGTCGCAATGA
TGGTAAAATTCCGAGTCCGCTGAGCGCAGATAGCCTGCCGGAAACCGCAATTATGCTGGCAGATGCAAGCCTGGCAGTTCTGGGTGCACCGCTGCCTGCAGA
TCGTGTTGAAAAAACCCTGGAACTGATTAAACAGGCAGATGCAAAATAACTCGGTACCAAAGACGAACAATAAGACGCTGAAAAGCGTCTTTTTTCGTTTTG
GTCCGCTTTGTATAAAGTCCGCCATTGGATCCAATTGACAGCTAGCTCAGTCCTAGGTACCATTGGATCCAATCGCTCATTCACTAGGTCTGATTCGTTACC
AATTGACAGCTGGTGGTCGAATCAAGATAATAGACCAGTCACTATATTTAGACTGTCGCCGGATGTGTATCCGACCTGACGATGGCCCAAAGGGCCGAAAC
AGTCCTCTACAAATAATTTTGTTTAACCAAACGAGGCCGGGAGGATGGAAAGCACCCCGACCAAACAGAAAGCAATTTTTAGCGCAAGCCTGCTGCTGTTTG
CAGAACGTGGTTTTGATGCAACCACCATGCCGATGATTGCAGAAAATGCAAAAGTTGGTGCAGGCACCATTTATCGCTATTTCAAAAACAAAGAAAGCCTGG
TGAACGAACTGTTTCAGCAGCATGTTAATGAATTTCTGCAGTGTATTGAAAGCGGTCTGGCAAATGAACGTGATGGTTATCGTGATGGCTTTCATCACATTT
TTGAAGGTATGGTGACCTTTACCAAAAATCATCCGCGTGCACTGGGTTTTATCAAAACCCATAGCCAGGGCACCTTTCTGACCGAAGAAAGCCGTCTGGCAT
ATCAGAAACTGGTTGAATTTGTGTGCACCTTTTTTCGTGAAGGTCAGAAACAGGGTGTGATTCGTAATCTGCCGGAAAATGCACTGATTGCAATTCTGTTTG
GCAGCTTTATGGAAGTGTATGAAATGATCGAGAACGATTATCTGAGCCTGACCGATGAACTGCTGACCGGTGTTGAAGAAAGCCTGTGGGCAGCACTGAGCC
GTCAGAGCTAACTCGGTACCAAATTCCAGAAAAGAGACGCTTTCGAGCGTCTTTTTTCGTTTTGGTCCCGCTACCAGGAATCTGAACGATTCGTTACCAATT
GCCATATTTAAAATTCTTGTTTAAAATGCTAGCCTTGTCCAACCAAATGATTCGTTACCCTTTGACAGTTTCTATCGATCTATAGATAATGCTAGCAGGAGT
CAATTAATGTGCTTTTAATTCTGATGAGACGGTGACGTCGAAACTCCCTCTACAAATAATTTTGTTTAATACGCTATGGACTATGTTTTCTGCTATGGACTA
TGTTTTCACACACGAGATGCCTCGATGAGCTATGGTGATAGCCGTGAAAAAATTCTGAGCGCAGCAACCCGTCTGTTTCAGCTGCAGGGTTATTATGGCACC
GGTCTGAATCAGATTATCAAAGAAAGCGGTGCACCGAAAGGTAGCCTGTATTATCATTTTCCGGGTGGTAAAGAACAGCTGGCAATTGAAGCAGTGAACGAA
ATGAAAGAATATATCCGCCAGAAAATCGCCGATTGTATGGAAGCATGTACCGATCCGGCAGAAGGTATTCAGGCATTTCTGAAAGAACTGAGCTGTCAGTTT
AGCTGTACCGAAGATATTGAAGGTCTGCCGGTTGGTCTGCTGGCAGCAGAAACCAGCCTGAAAAGCGAACCGCTGCGTGAAGCATGTCATGAAGCATATAAA
GAATGGGCCAGCGTGTATGAAGAAAAACTGCGTCAGACCGGTTGTAGCGAAAGCCGTGCAAAAGAAGCAAGCACCGTTGTTAATGCAATGATTGAAGGTGGT
ATTCTGCTGAGCCTGACCGCAAAAAATAGCACACCGCTGCTGCATATTAGCAGCTGTATTCCGGATCTGCTGAAACGTTAATAAGGTTGAAAAATAAAAACG
GCGCTAAAAAGCGCCGTTTTTTTTGACGGTGGTATGTCCCCTTTGTGCGTCCAAACGGACGCACGGCGCTCTAAAGCGGGTCGCGATCTTTCAGATTCGCTC
CTCGCGCTTTCAGTCTTTGTTTTGGCGCATGTCGTTATCGCAAAACCGCTGCACACTTTTGCGCGACATGCTCTGATCCCCCTCATCTGGGGGGGCCTATCT
GAGGGAATTTCCGATCCGGCTCGCCTGAACCATTCTGCTTTCCACGAACTTGAAAACGCTAGTAGTCACCGGCTGTGCTTGCCGGTCTGATGAGCCTGTGAA
GGCGAAACTACCTCTACAAATAATTTTGTTTAAACCCCCGAGATGAAATACATCCTGTTTGAGGTGTGCGAAATGGGTAAAAGCCGTGAACAGACCATGGAA
AATATTCTGAAAGCAGCCAAAAAGAAATTCGGCGAACGTGGTTATGAAGGCACCAGCATTCAAGAAATTACCAAAGAAGCCAAAGTTAACGTTGCAATGGCC
AGCTATTACTTTAATGGCAAAGAGAACCTGTACTACGAGGTGTTCAAAAAATACGGTCTGGCAAATGAACTGCCGAACTTTCTGGAAAAAAACCAGTTTAAT
CCGATTAATGCCCTGCGTGAATATCTGACCGTTTTTACCACCCACATTAAAGAAAATCCGGAAATTGGCACCCTGGCCTATGAAGAAATTATCAAAGAAAGC
GCACGCCTGGAAAAAATCAAACCGTATTTTATCGGCAGCTTCGAACAGCTGAAAGAAATTCTGCAAGAGGGTGAAAAACAGGGTGTGTTTCACTTTTTTAGC
ATCAACCATACCATCCATTGGATTACCAGCATTGTTCTGTTTCCGAAATTCAAAAAATTCATCGATAGCCTGGGTCCGAATGAAACCAATGATACCAATCAT
GAATGGATGCCGGAAGATCTGGTTAGCCGTATTATTAGCGCACTGACCGATAAACCGAACATTTAAGTTATGAGTCAGGAAAAAAGGCGACAGAGTAATCTG
TCGCCTTTTTTCTTTGCTTGCTTTTCTGACCAGGAATCTGAACGATTCGTTACCAATTGCCATATTTAAAATTCTTGTTTAAAATGCTAGCTACTCCACCGT
TGGCTTTTTTCCCTATCAGTGATAGAGATTGACATCCCTATCAGTGATAGAGATAATGAGCACAGAAGTCAATTAATGTGCTTTTAATTCTGATGAGTCGGT
GACGACGAAACTTCCTCTACAAATAATTTTGTTTAACCCCCCGAGGAGTAGCACATGCCGAAACTGGGTATGCAGAGCATTCGTCGTCGTCAGCTGATTGAT
GCAACCCTGGAAGCAATTAATGAAGTTGGTATGCATGATGCAACCATTGCACAGATTGCACGTCGTGCCGGTGTTAGCACCGGTATTATTAGCCATTATTTC
CGCGATAAAAACGGTCTGCTGGAAGCAACCATGCGTGATATTACCAGCCAGCTGCGTGATGCAGTTCTGAATCGTCTGCATGCACTGCCGCAGGGTAGCGCA
GAACAGCGTCTGCAGGCAATTGTTGGTGGTAATTTTGATGAAACCCAGGTTAGCAGCGCAGCAATGAAAGCATGGCTGGCATTTTGGGCAAGCAGCATGCAT
CAGCCGATGCTGTATCGTCTGCAGCAGGTTAGCAGTCGTCGTCTGCTGAGCAATCTGGTTAGCGAATTTCGTCGTGAACTGCCTCGTGAACAGGCACAAGAG
GCAGGTTATGGTCTGGCAGCACTGATTGATGGTCTGTGGCTGCGTGCAGCACTGAGCGGTAAACCGCTGGATAAAACCCGTGCAAATAGCCTGACCCGTCAT
TTTATCACCCAGCATCTGCCGACCGATTAACCAATTATTGAACACCCTTCGGGGTGTTTTTTTGTTTCTGGTCTCCCGGGCAACGATCGTTGGCTGTGTTGA
CAATTAATCATCGGCTCGTATAATGTGTGGAATTGTGAGCGCTCACAATTAGCGCGGGTGAGAGGGATTCGTTACCAATAGACAATTGATTGGACGTTCAAT
ATAATGCTAGCAGTACGTCTGAGCGTGATACCCGCTCACTGAAGATGGCCCGGTAGGGCCGAAACGTACCTCTACAAATAATTTTGTTTAAGACTTAAACGA
TTGGCCGACCGCATGAGCCCGAAACGTCGTACCCAGGCAGAACGTGCAATGGAAACCCAGGGTAAACTGATTGCAGCAGCACTGGGTGTTCTGCGTGAAAAA
GGTTATGCAGGTTTTCGTATTGCAGATGTTCCGGGTGCAGCCGGTGTTAGCCGTGGTGCACAGAGCCATCATTTTCCGACCAAACTGGAACTGCTGCTGGCA
ACCTTTGAATGGCTGTATGAGCAGATTACCGAACGTAGCCGTGCACGTCTGGCAAAACTGAAACCGGAAGATGATGTTATTCAGCAGATGCTGGATGATGCA
GCAGAATTTTTTCTGGATGATGATTTTAGCATCAGCCTGGATCTGATTGTTGCAGCAGATCGTGATCCGGCACTGCGTGAAGGTATTCAGCGTACCGTTGAA
CGTAATCGTTTTGTTGTTGAAGATATGTGGCTGGGTGTGCTGGTGAGCCGTGGTCTGAGCCGTGATGATGCCGAAGATATTCTGTGGCTGATTTTTAACAGC
GTTCGTGGTCTGGCAGTTCGTAGCCTGTGGCAGAAAGATAAAGAACGTTTTGAACGTGTGCGTAATAGCACCCTGGAAATTGCACGTGAACGTTATGCAAAA
TTCAAACGTTGATAACTCGGTACCAAAGAAAAATAAAAAGACGCTGAAAAGCGTCTTTTTATTTTTCGGTCCAATG

a. DNA sequence colors: AmeR (light blue), AmtR (cyan), BetI (blue), BM3R1 (red), CymR (dark blue), HlyIIR (green), LmrA (orange red), PhlF (orange), SrpR (dark green), and VanR (light green).

References

Ackers GK, Johnson AD, Shea MA (1982) Quantitative model for gene regulation by lambda phage repressor. *Proceedings of the National Academy of Sciences* **79:** 1129

Brophy JAN, Voigt CA (2016) Antisense transcription as a tool to tune gene expression. *Molecular Systems Biology* **12:** 854

Dahirel V, Paillusson F, Jardat M, Barbi M, Victor J-M (2009) Nonspecific DNA-Protein Interaction: Why Proteins Can Diffuse along DNA. *Physical Review Letters* **102:** 228101

Roberts JW (2014) Molecular basis of transcription pausing. *Science* **344:** 1226

Nielsen AAK, Der BS, Shin J, Vaidyanathan P, Paralanov V, Strychalski EA, Ross D, Densmore D, Voigt CA (2016) Genetic circuit design automation. *Science* **352:** aac7341
